# Human Milk Oligosaccharides Multivalently Presented on Defined Synthetic Neo-Glycoproteins Are Nanomolar Ligands of Tandem-Repeat Galectins

**DOI:** 10.1021/acs.biomac.5c00377

**Published:** 2025-07-07

**Authors:** Jakub Červený, Viktoria Heine, Michaela Hovorková, Petr Brož, Eliška Filipová, Natalia Kulik, Martin Hubálek, Josef Cvačka, Lucie Petrásková, Mirane Florencio-Zabaleta, Sandra Delgado, Helena Pelantová, Zuzana Bosáková, Lothar Elling, Jesus Jiménez-Barbero, Ana Ardá, Vladimír Křen, Pavla Bojarová

**Affiliations:** † 86863 Institute of Microbiology of the Czech Academy of Sciences, Vídeňská 1083, CZ-142 00 Prague 4, Czech Republic; ‡ Department of Analytical Chemistry, Faculty of Science, Charles University, Hlavova 8, CZ-128 43 Prague 2, Czech Republic; § Department of Genetics and Microbiology, Faculty of Science, 112302 Charles University, Viničná 5, CZ-128 43 Prague 2, Czech Republic; ∥ Department of Biochemistry and Microbiology, University of Chemistry and Technology Prague, Technická 3, CZ-166 28 Prague 6, Czech Republic; ⊥ 89220 Institute of Organic Chemistry and Biochemistry of the Czech Academy of Sciences, Flemingovo Náměstí 2, CZ-166 10 Prague 6, Czech Republic; # Laboratory for Biomaterials, Institute of Biotechnology and Helmholtz Institute for Biomedical Engineering, 9165 RWTH Aachen, Pauwelstr. 20, D-52079 Aachen, Germany; g CICbioGUNE, Basque Research and Technology Alliance, Parque Científico Tecnológico de Bizkaia Building 801A, E-48160 Derio, Spain; h Ikerbasque, Basque Foundation for Science, Plaza Euskadi 5, E-48009 Bilbao, Spain; i Department of Inorganic & Organic Chemistry, Faculty of Science and Technology, University of the Basque Country, UPV/EHU, E-48940 Leioa, Spain; j Centro de Investigación Biomedica En Red de Enfermedades Respiratorias, C/ Monforte de Lemos, 3-5. Pabellón 11, Planta 0, Fuencarral-El Pardo, E-28029 Madrid, Spain

## Abstract

Galectins are small human proteins participating in inflammation processes, immune response, and cancerogenesis. Tandem-repeat galectins comprising Gal-4, Gal-8, and Gal-9 are a vital yet less studied part of the galectin fingerprint in cancer-related processes. The present work studies a library of prepared multivalent neo-glycoproteins decorated with poly-*N*-acetyllactosamine and human-milk-type oligosaccharides as ligands of this underexplored family of tandem-repeat galectins. A thorough binding evaluation by ELISA and biolayer interferometry was complemented with a detailed epitope mapping both from the galectin and the glycoconjugate viewpoints by nuclear magnetic resonance. The found interactions in the galectin binding site were correlated to *in silico* data from molecular modeling. The present work reveals pioneer information on the binding of tandem-repeat galectins to multivalent glycoconjugates carrying complex carbohydrate ligands and represents an invaluable starting point for the development of new high-affinity tailored ligands of tandem-repeat galectins, needed both for diagnosis and therapy.

## Graphical abstract

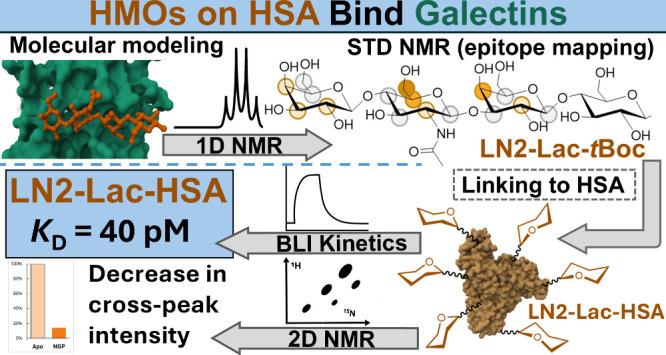

## 1 Introduction

Galectins (Gal-) play a crucial role in various disease-related processes in humans, such as inflammation processes and immune response, cancer processes, or metabolic disorders.^1,2^ Different types of galectins, out of the 12 representatives in humans, seem to interact by specific mechanisms, *e.g*., in the downregulation of genes mediating insulin uptake, showing a combined effect.^3^ Thereby the individual impacts of single galectins in complex processes may not be easy to distinguish. While Gal-3 (chimera-type galectin) and Gal-1 (prototype galectin) are thoroughly investigated, the family of tandem-repeat galectins comprising Gal-4, Gal-8, and Gal-9 is by far less explored. Tandem-repeat galectins consist of an N-terminal and a C-terminal carbohydrate recognition domain (CRD) interconnected with a peptide linker.^4^ Both domains share a certain degree of identity, *e.g*., 40% in Gal-8,^5^ but they differ in the affinity to glycan ligands: Gal-8C binds to nonsialylated glycans, such as poly-LacNAc oligosaccharides, while Gal-8N binds to α­(2-3)-sialylated and sulfated glycans.^6^ Gal-9 subunits bind poly-LacNAc glycans, with a higher preference of the N-subunit for nonsialylated patterns.^7^ Interestingly, Gal-4N demonstrated a significantly higher affinity for sulfated glycans,^8,9^ and galactosyl-capped tetrasaccharides^10^ compared to Gal-4C. While the roles of Gal-1 and Gal-3 in cancer progression, inflammation, fibrosis, heart disease, stroke, and some metabolic disorders are well established, the contributions and pathophysiological roles of tandem repeat galectins, including the preferences of their respective subunits, remain less understood. One of the reasons is the unavailability of well-defined ligands with submicromolar affinity.

To increase the affinity of galectins to their ligands for future therapeutic applications, multivalency in various arrangements was studied in the past. Since even single-domain galectins commonly cluster into lattices or oligomers with multiple CRDs, simultaneous binding of several glycan residues is possible. Solid particles,^11,12^ polymeric structures,^13–16^ proteins or peptides^17–20^ or dendrimers^21^ served as scaffolds and facilitated the multivalent presentation of carbohydrate residues on their surface. The binding affinity of galectins was thus increased manifold due to multivalency effects. For example, neo-glycoproteins carrying LacdiNAc (GalNAcβ4GlcNAc)^18,22^ or its glycomimetics^19^ increased the inhibitory potency toward Gal-3 more than 10^3^-fold compared to free parent LacdiNAc. These neo-glycoproteins were also able to reduce Gal-3-mediated apoptosis of lymphocytes and served as extracellular Gal-3 inhibitors.^19^ In contrast to the multitude of works on the most studied Gal-1 and Gal-3, targeted inhibition of tandem-repeat galectins is vastly underexplored. Quintana et al.^23^ and Slámová et al.^24^ focused on the interactions with naturally occurring (monovalent) inhibitors, such as blood group antigens and human milk oligosaccharides. In contrast, Konvalinková et al.,^25^ Müllerová et al.,^26^ Vrbata et al.,^27^ and Pal et al.^28^ explored synthetic and semisynthetic ligands. Vrbata’s work concentrated on synthetic small-molecule inhibitors with various electron-deficient and electron-rich substituents. Pal extensively screened the inhibitory effects of galactose-based compounds modified with quinoline, indolizines, or coumarins. The aspect of multivalency was partially touched upon in the studies with glycodendrimers and glycocalixarenes^25,26^ although the observed multivalency effects were rather insignificant. Obviously,^23–27^ none of the inhibitors of the state of the art has broken the nanomolar affinity border for tandem-repeat galectins. Therefore, the identification of prospective high-affinity ligands of tandem-repeat galectins is enormously attractive for biomedical, and pharmaceutical research.

This study investigates the binding behavior of tandem-repeat galectins (Gal-4, Gal-8, and Gal-9) to mono- and multivalent functionalized tetrasaccharide ligands presented on a human serum albumin carrier. We prepared a library of functionalized tetrasaccharides structurally related to poly-LacNAc and human milk oligosaccharides, which are highly biologically relevant for infant metabolism^29^ and play a key role in immune defense,^30^ and turned them into human-serum-albumin-based neo-glycoproteins. We elucidated the binding behavior of tandem-repeat galectins Gal-4, Gal-8, and Gal-9 toward both mono- and multivalent forms of these tetrasaccharides. Affinities to Gal-1 and Gal-3 benchmarks were assessed for comparison. While many studies focus only on ligands for single selected domains of tandem-repeat galectins,^31,32^ in this work we present the data for full-length tandem-repeat galectins, which better reflect the actual applicability *in vivo*. To complete the picture, selected ligands were also tested with individual galectin subunits. Besides ELISA-type assays to monitor galectin affinity, we have employed nuclear magnetic resonance (NMR) spectroscopy and biolayer interferometry (BLI) using monobiotinylated galectin constructs^18^ to further characterize the galectin-ligand interactions. NMR, both from the perspective of the lectin (by employing ^15^N-labeled galectins) and of the ligand (through STD-NMR experiments) has provided atomic-level details about the binding epitopes in the ligand-lectin system.^33^ In parallel, BLI, a label-free optical technique, enabled real-time measurement of binding kinetics and affinities under near-physiological conditions. The collected data were aligned with the conclusions of molecular modeling. Molecular dynamics simulation and docking allowed us to identify the amino acid residues involved in ligand binding and to characterize the structural differences defining ligand affinities. These complementary techniques provide a comprehensive understanding of galectin-ligand interactions, giving crucial insights for the development of multivalent therapeutic inhibitors.

## 2 Materials and Methods

### 2.1 General Procedure of Glycan Synthesis

The enzymatic glycosylation cascade reactions affording lactose-based tetrasaccharides **3** (**LN1-Lac**-*t*Boc; Galβ3­GlcNAc­β3Gal­β4Glc-*t*Boc), **4** (**LN2-Lac**-*t*Boc; Galβ4­GlcNAc­β3Gal­β4Glc-*t*Boc), **5** (**LDN-Lac**-*t*Boc; GalNAc­β4Glc­NAcβ3­Gal­β4Glc-*t*Boc) and LacNAc-based tetrasaccharides **9** (**LN1-LN2**-*t*Boc; Galβ3­Glc­NAc­β3Gal­β4Glc­NAc-*t*Boc), **10** (**LN2-LN2**-*t*Boc; Galβ4­GlcNAc­β3Gal­β4Glc­NAc-*t*Boc), **11** (**LDN-LN2**-*t*Boc; GalNAc­β4Glc­NAcβ3­Galβ4­GlcNAc-*t*Boc) started from the respective synthetic precursors: (*tert*-butoxycarbonylamino)­ethylthioureidyl β-d-galactopyranosyl-(1→4)-β-d-glucopyranoside (Lac-*t*Boc; Galβ4Glc-*t*Boc; **1**), and (*tert*-butoxycarbonylamino)­ethylthioureidyl 2-acetamido-2-deoxy-β-d-glucopyranoside (GlcNAc-*t*Boc; **6**). The *t*Boc-protected precursor Lac-*t*Boc **1** ^18,24–26,34^ (for HRMS and HPLC analysis see Supporting Information, Section 5) was prepared analogously to the established procedure for *t*Boc-protected precursor GlcNAc-*t*Boc **6** ^35^ (for HRMS and HPLC analysis see Supporting Information, Section 5). LN2-*t*Boc (**7**; Galβ4GlcNAc-*t*Boc) was prepared as described previously from GlcNAc-*t*Boc (**6**) under the catalysis by β-galactosidase from *B. circulans.* ^14^ The overview of enzymes used for glycosylations and respective reaction conditions are summarized in Supporting Information, Table S1. In all enzymatic steps with glycosyltransferases, the respective glycosyl acceptor (**1**, **2**, **7**, or **8**; 5 mM) was mixed with the glycosyl donor (UDP-GlcNAc or UDP-Gal or UDP-GalNAc; 6.5 mM) and suspended in the respective buffer. Then, the respective glycosyltransferase was added, and the reactions were incubated overnight at 37 °C under shaking at 350–450 rpm. For the synthesis of tetrasaccharides **3** (**LN1-Lac**-*t*Boc) and **9** (**LN1-LN2**-*t*Boc), trisaccharide acceptors (10 mM) of **GlcNAc-Lac**-*t*Boc (**2**; GlcNAcβ3Galβ4Glc-*t*Boc) or **GlcNAc-LN2**-*t*Boc (**8**; GlcNAcβ3Galβ4GlcNAc-*t*Boc), respectively, were glycosylated using α-d-galactosyl fluoride (α-Gal-F) as a donor under the catalysis by mutant β-galactosidase BgaC-E233G. All enzymatic reactions were stopped by thermal enzyme inactivation at 99 °C for 5 min, and the enzyme-free reaction mixture was first purified by solid phase extraction using C18 ec Chromabond SPE column (Macherey-Nagel, Germany) conditioned with 15 mL of methanol and 15 mL of water. The sample was applied in the respective buffer, washed with 5 mL of 5% methanol, eluted with 5 mL of 100% methanol, and subsequently purified by gel permeation chromatography (GPC; Biogel P2, 1000 × 30 mm, H_2_O mobile phase; 6 mL/h flow rate). In case the final products were not completely pure after size exclusion chromatography, they were repurified by HPLC (reversed-phase analytic MultoKrom 100–5 C18 column; 250 × 4.6 mm; CS Chromatographie, Langerwehe, Germany) with 85/15, *v*/*v*, H_2_O/acetonitrile as a mobile phase, at a flow rate of 1 mL/min, with detection at 220 nm. The fractions containing the pure product were pooled and lyophilized.

### 2.2 Synthesis of Neo-Glycoproteins

In the first step, *t*Boc-capped oligosaccharides **1**, **3**, **4**, **5**, **7**, **9**, **10**, and **11** (10 mM) were deprotected in 1 M HCl to obtain the respective free amines. The reaction mixtures were incubated for 48 h at 4 °C and the complete conversion to the deprotected sugars was confirmed by TLC (Merck silica gel DC-Alufolien Kieselgel 60 F_254_ plates; mobile phase: isopropyl alcohol/H_2_O/NH_4_OH aq; 7/2/1, *v*/*v*/*v*; visualization by charring with 5% H_2_SO_4_ in EtOH). The reactions were neutralized by dilution with water and the addition of Dowex 66 base-free. Deprotected glycans were freeze-dried and directly used. In the next step, the compounds were coupled to squaric acid diethyl ester (10 mM deprotected glycan, 40 mM squaric acid diethyl ester, 40 mM triethylamine, 35 mM HEPES pH 7.0, 50%, *v*/*v*, ethanol) *via* the free amine. The compounds were separated from unreacted squaric acid diethyl ester and deprotected sugar by HPLC (Section 2.1) and analyzed by HPLC and MS (Supporting Information, Figures S12a–S19d). In the third step, pure squarate monoamide esters **1a**, **3a**, **4a**, **5a**, **7a**, **9a**, **10a**, or **11a** were coupled to HSA (4 mg/mL HSA in 50 mM tetraborate buffer pH 9.0) for 72 h (shaking at 500 rpm at ambient temperature). The squarate monoamide esters **1a**, **3a**, **4a**, **5a**, **7a**, **9a**, **10a**, or **11a** were added to HSA in a concentration depending on the intended occupation per molecule HSA (for an occupation of approximately 6 sugar residues per molecule HSA we applied a 2.5-fold excess). The neo-glycoproteins were then washed with water by ultracentrifugation, and the glycan occupancy was determined using matrix-assisted laser desorption/ionization time-of-flight mass spectrometry (MALDI-TOF). The molecular weight of HSA (control) was subtracted from the total molecular weight of NGPs, and subsequently divided by the molecular weight of conjugated glycan, yielding the number of bound glycan epitopes (Supporting Information, Figures S21–S28, Table S7). The neo-glycoprotein purity was confirmed by SDS-PAGE (10% gel; Supporting Information, Figure S20).

### 2.3 Galectin Constructs

Full-length recombinant human galectins (Gal-1, Gal-3, Gal-4, Gal-8, and Gal-9) for ELISA assays were produced as N-terminal His-tagged constructs in the pET-Duet1 vector, prepared as previously described.^18,24–26,34^ Selectively monobiotinylated galectin constructs for biolayer interferometry (BLI) carried an AVI-tag sequence (GLND­IFEA­Q**K**IE­WHE), which was biotinylated under the action of a biotin ligase, coexpressed during heterologous production. In all galectin constructs, the AVI-tag is located at the N-terminus, and, except for Gal-3, a 15-residue linker was introduced between the AVI-tag and the galectin CRD gene to preserve their lectin activity by maintaining the flexibility and accessibility of the CRD domain after protein immobilization. In Gal-3, no linker was necessary as this protein contains a flexible N-terminal domain preceding the C-terminal CRD. The AVI-tagged galectin constructs were shown not to differ in affinities from the original His-tagged galectin constructs with a series of positive control ligands (ASF – direct binding, Lac, LacNAc, **NGP1**). Productions of both unlabeled and ^15^N-labeled galectin constructs for NMR studies were done as previously described.^23^ More details can be found in Supporting Information, Section 6.

### 2.4 Enzyme-Linked Immunosorbent Assay (ELISA)

The inhibitory potential of the prepared glycan library and the respective multivalent neo-glycoproteins with Gal-1, -3, -4, -8, and -9 was evaluated using a competitive ELISA assay as described by Vašíček et al.^34^ Asialofetuin (ASF; 0.1 μM in PBS, 50 μL/well) was immobilized onto microtiter plates (Nunc Immuno Sorb, ThermoFisher Scientific, USA) overnight at 4 °C. Wells were subsequently washed three times with PBS containing 0.05%, *v*/*v*, Tween-20 (250 μL/wash). Each assay step was followed by a washing step to ensure the thorough removal of unbound reagents. Blocking was performed using 2%, *w*/*v*, BSA in PBS (250 μL/well) for 1 h at room temperature. Serial dilutions of the inhibitors prepared in EPBS buffer (PBS containing 2 mM EDTA) were incubated with a constant concentration of galectin (2.5 μM Gal-1, Gal-3, and Gal-4; 0.5 μM Gal-8 and Gal-9) for 2 h. Galectin concentrations used in the inhibition assay were determined based on their binding affinity to ASF (Supporting Information, Table S8). Residual galectin bound to ASF was detected using the anti-His-tag antibody conjugated to horseradish peroxidase (Santa Cruz Biotechnology, 1:999 dilution in PBS). Detection was achieved by adding 50 μL of TMB One substrate solution (Kem-En-Tech, Denmark), which developed a blue color that turned yellow upon the addition of 50 μL of 3 M HCl. Absorbance at 450 nm was measured using a microplate reader (Sunrise, Tecan Group Ltd., Switzerland) reflecting the amount of galectin bound to the wells. Half-maximal inhibitory concentration (IC_50_) values were calculated by nonlinear regression (dose–response inhibition with variable slope) of sigmoidal curves using GraphPad Prism version 8.4.3 (GraphPad Software, USA).

### 2.5 Affinity Studies by Nuclear Magnetic Resonance (NMR)

#### General Information

Precision NMR tubes with 3 mm outer diameter (New Era Enterprises, Vineland, USA) were used with a total sample volume of 180 μL in all NMR experiments of this section. Buffer pH was measured with pH-meter Crison Basic 20 (Crison Instruments SA, Barcelona, Spain) and adjusted with the required amount of NaOH and HCl.

#### ^1^H,^15^N-Heteronuclear Single Quantum Coherence Experiments (HSQC)

^15^N-HSQC experiments were acquired using Bruker AVANCE 2 800 MHz spectrometer equipped with a cryoprobe. Samples containing 50 μM ^15^N labeled Gal-4 or Gal-8 or 14 μM Gal-9 in 90% phosphate buffer (50 mM sodium phosphate, 150 mM NaCl, pH 7.4) with 10% D_2_O supplemented with 1 mM dithiothreitol (DTT) and 0.1% NaN_3_. All experiments were acquired at 308 K. The neo-glycoproteins were titrated in the protein samples (0.6, 1.1, and 1.6 equiv of carbohydrate epitope) and ^1^H–^15^N-HSQC experiments were acquired at each intermediate point. The backbone assignments were done using CcpNmr Analysis software based on the previously published procedures.^36,37^

#### ^1^H-Saturation-Transfer Difference Nuclear Magnetic Resonance Experiment (STD-NMR)

The experiments were performed using a Bruker AVANCE 2800 MHz spectrometer equipped with a cryoprobe. Samples were prepared in deuterated phosphate-buffered saline (50 mM sodium phosphate/150 mM NaCl pH 7.4) supplemented with 1 mM dithiothreitol-*d*_10_ (DTT-*d*_10_) and 0.1% NaN_3_. All measurements were conducted at 308 K. The ligand-to-galectin ratio was set to 50:1, with protein concentrations of 40 μM for Gal-4 and Gal-8, and 14 μM for Gal-9. STD experiments utilized a custom 1D-STD pulse sequence with 90° PC9 pulses for protein saturation and a 100 ms T1ρ protein filter. Spectra were acquired with 4608 scans, a total saturation time of 2 s, and a relaxation delay of 3 s. On- and off-resonance spectra were collected in an interleaved manner with equal numbers of scans. On-resonance saturation frequencies were set at 0.61–0.91 ppm for the aliphatic region and 6.99 ppm for the aromatic region, while the off-resonance saturation frequency was set at 30 ppm. STD NMR spectra were generated by subtracting the on-resonance spectra from the off-resonance spectra. Analysis of the spectra was based on the proton signal exhibiting the strongest STD effect, which was assigned as the reference (100% STD effect). Relative STD intensities for other protons within the molecule were calculated accordingly.

### 2.6 Biolayer Interferometry (BLI)

The binding kinetics of the best-performing neo-glycoprotein **NGP5** and, for comparison, lactose-loaded neo-glycoprotein **NGP1** to galectin constructs (Gal-4, Gal-8, Gal-9) and their respective subunits were assessed using biolayer interferometry (BLI). Experiments were conducted under controlled conditions (25 ± 0.1 °C, 850 rpm) with an Octet Red96e instrument (FortéBio, Fremont, CA, USA). Before kinetic measurements, scout experiments were performed with serial dilutions of full-length galectin constructs or galectin subunits (0.625–10 μg/mL) in the presence of 100 nM **NGP1** or **NGP5**. The optimal galectin concentration of 2 μg/mL, providing the best fit and response-to-noise ratio, was used in subsequent experiments. Biotinylated galectin constructs were diluted to 2 μg/mL in PBS containing 0.05% Tween-20 and immobilized on a streptavidin-coated biosensor (Octet SA Biosensors, Sartorius, Göttingen, Germany) *via* biotin–streptavidin coupling. Following immobilization (180 s), the interactions between the immobilized constructs and serially diluted neo-glycoproteins (1.56–100 nM) were monitored over a total of 1050 s, including an association phase (450 s) and a dissociation phase (600 s). It was confirmed that immobilization did not affect galectin activity. BLI data were analyzed using Octet Analysis Studio (Sartorius, Göttingen, Germany). Background from nonspecific interactions (<5% of the total response) and the sensor drift were corrected using a double-reference subtraction method. Kinetic data for individual galectin subunits were analyzed by a 1:2 bivalent binding model and subsequently evaluated with the Langmuir 1:1 kinetic model. Data for full-length galectin constructs were analyzed by a 2:1 heterogeneous binding model and evaluated with the Langmuir 1:1 model. All data sets were also validated using steady-state analysis (results not shown).

### 2.7 Molecular Modeling

Galectin structures for molecular dynamics simulation and docking were downloaded from the Protein Data Bank:^38^ Gal-4N from 5dux,^9^ Gal-4C from 4ylz,^8^ Gal-8C and Gal-8N from 3vkl,^39^ Gal-9N from 2zhl,^40^ and Gal-9C for 3nv1.^41^

Ligands were modeled in YASARA based on the **LN2-LN2** ligand in Gal-9N (pdb ID: 2zhl). Docking prior to molecular dynamics simulation was done with the Lamarkian Genetic Algorithm in AutoDock4.^42^ The lowest energy poses of ligands with low RMSD to the reference structure (from 2zhl) were selected and used for molecular dynamics simulation, which was run in YASARA according to the protocol described previously.^27^ The simulations were run for 100 ns and analyzed with YASARA tools, and ProLIF.^43^ Binding scores after molecular dynamics simulation were calculated by AutoDock Vina^44^ by scoring in place of 15 snapshots from the last 20 ns of molecular dynamics simulation acquired at 1.3 ns time intervals.

## 3 Results and Discussion

### 3.1 Production of Glycans

In the first step, monovalent glycans were prepared by sequential chemo-enzymatic synthesis as detailed in Supporting Information, Section 4. The synthetic precursors Lac-*t*Boc (**1**, Figures S2a and S2b) and GlcNAc-*t*Boc (**6**) were chemically synthesized in high yields according to the previously described method.^35^ The architecture of the *t*Boc-protected thiourea-based linker has been selected due to its positive influence on the efficiency of enzymatic glycosylation, probably through nonspecific interactions with the enzyme active site.^45^ Furthermore, the free amino group originated after deprotection enables direct conjugation to HSA-contained lysines. We observed no significant influence of the thiourea linker on galectin binding. For LacNAc-based oligosaccharides, we started with GlcNAc-*t*Boc (**6**) and applied β-galactosidase from *B. circulans,* or, alternatively, human β4GalT,^46^ to obtain LN2-*t*Boc (**7**, Supporting Information, Figures S7a and S7b) as described previously.^14^ In the next step, Lac-*t*Boc (**1**) and LN2-*t*Boc (**7**) were treated in the same way (Scheme 1, Supporting Information, Figure S1). After elongation with β3GlcNAcT from *Helicobacter pylori,* ^47^ both trisaccharides, **GlcNAc-Lac**-*t*Boc (**2**; Figures S3a–S3d, Table S2), or **GlcNAc-LN2**-*t*Boc (**8**; Figures S8a–S8d, Table S6), were used as acceptors for the synthesis of tetrasaccharides. LacNAc type 1 terminated tetrasaccharides **LN1-Lac**-*t*Boc (**3**; Figures S4a–S4d, Table S3), and **LN1-LN2**-*t*Boc (**9**; Figures S9a and S9b) were prepared using mutant E233G β3-galactosidase (BgaC-E233G) from *B. circulans.* ^48^ LacNAc type 2 terminated tetrasaccharides **LN2-Lac**-*t*Boc (**4**; Figures S5a–S5d, Table S4), and **LN2-LN2**-*t*Boc (**10**; Figures S10a and S10b) were produced using human placental β4-galactosyltransferase β4GalT.^46^ LacdiNAc capped tetrasaccharides **LDN-Lac**-*t*Boc (**5**; Figures S6a–S6d, Table S5), and **LDN-LN2**-*t*Boc (**11**; Figures S11a and S11b) were afforded by the action of mutant human placental β4-galactosyltransferase with β4-*N*-acetylgalactosaminyltransferase activity (β4GalT-Y284L = β4GalNAcT).^49^ Between each step, glycans were purified by solid-phase extraction using the C18 ec column (SPE) followed by gel permeation chromatography (GPC). If the final products were not completely pure, they were purified by reversed-phase HPLC. The analysis of all prepared structures and their purity (NMR, HPLC, HRMS) is documented in Supporting Information, Section 5.

**Scheme 1:**
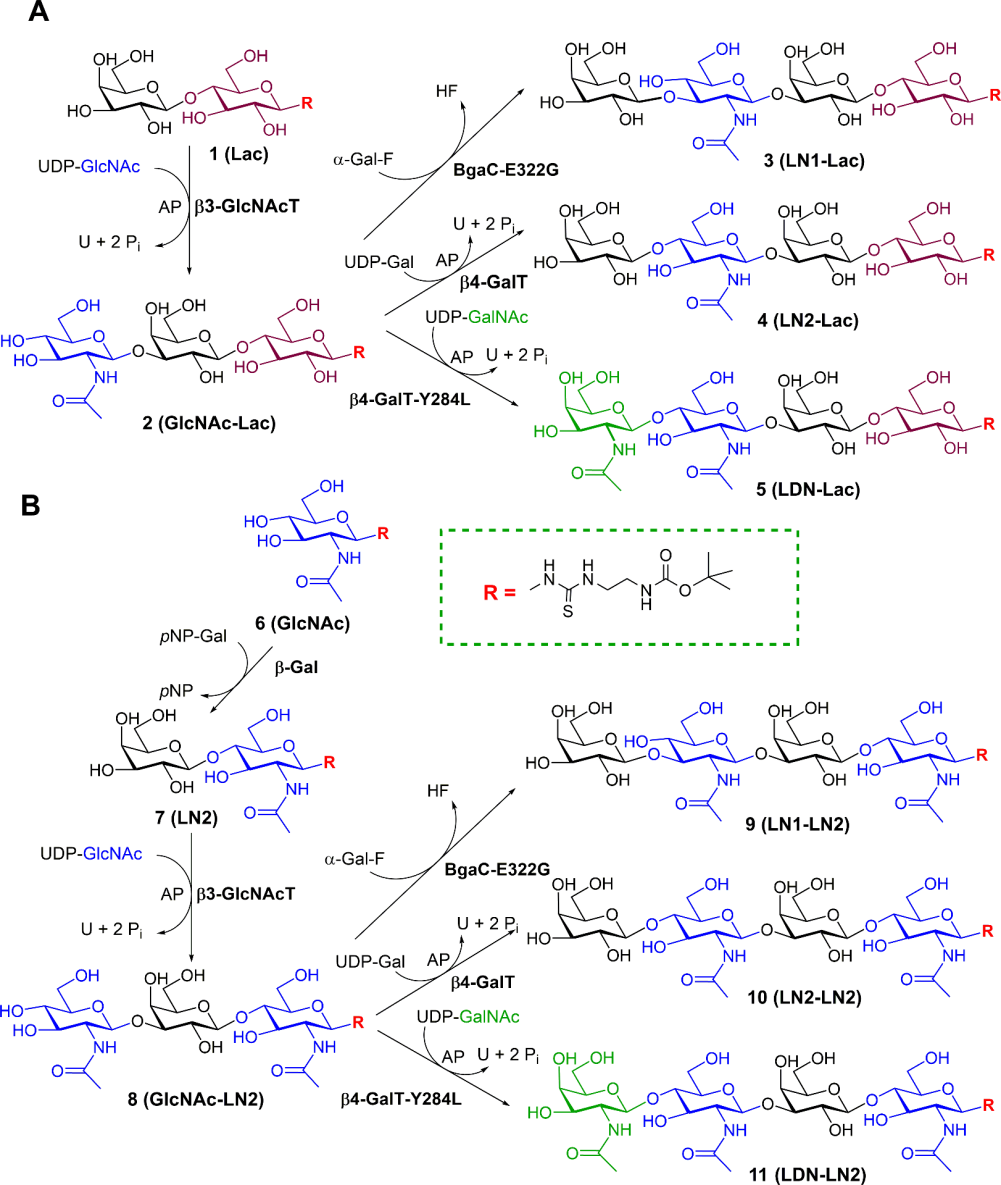
Glycan Synthesis: (A) Synthesis of Lactose-Based Tetrasaccharides; (B) Synthesis of LacNAc-Based Tetrasaccharides^a^

a Glc is depicted in burgundy, Gal in black, GlcNAc in blue, and GalNAc in green.

### 3.2 Production of Neo-Glycoproteins

To achieve an increased affinity through multivalency, functionalized glycans were attached to human serum albumin (HSA) as a carrier. HSA exhibits several favorable properties, including excellent solubility, high stability, and a well-characterized structure. Its numerous surface-exposed lysine residues enable high-density site-accessible glycan attachment through primary amines. In our previous studies, HSA showed superior performance over other scaffolds in terms of maximum cluster-glycoside effect.^24–26^ The coupling procedure started with the deprotection of the *t*Boc group to afford free amines at the reducing end of the sugar. The free amines were coupled with squaric acid diethyl ester, which served as a linker for the attachment to the protein (Scheme 2). The resulting tetrasaccharide squarate monoamides **1a**, **3a**, **4a**, **5a**, **7a**, **9a**, **10a**, or **11a** were purified by reverse phase HPLC, and the respective mass was confirmed by ESI-MS (Supporting Information, Figures S12a–S19b). Since the reactivity of squaric acid diethyl ester is tunable by pH, successive binding to the deprotected glycan and then to HSA is possible. This feature enabled a customized occupation of HSA with modified glycans. HSA was incubated for 72 h with a defined amount of tetrasaccharide squarate monoamide to obtain a defined neo-glycoprotein with an occupancy of approximately 5 to 9 glycans per one HSA molecule, which proved to be optimal to achieve a high affinity to target galectins.^17,19^ Although the concentrations were kept constant in all samples, HSA occupancy was not the same for every neo-glycoprotein. Some glycans probably sterically hindered the attachment to HSA, so that their attachment was reduced. The purity and homogeneity of all neo-glycoproteins were confirmed by SDS-PAGE (Supporting Information, Figure S20).

**Scheme 2:**
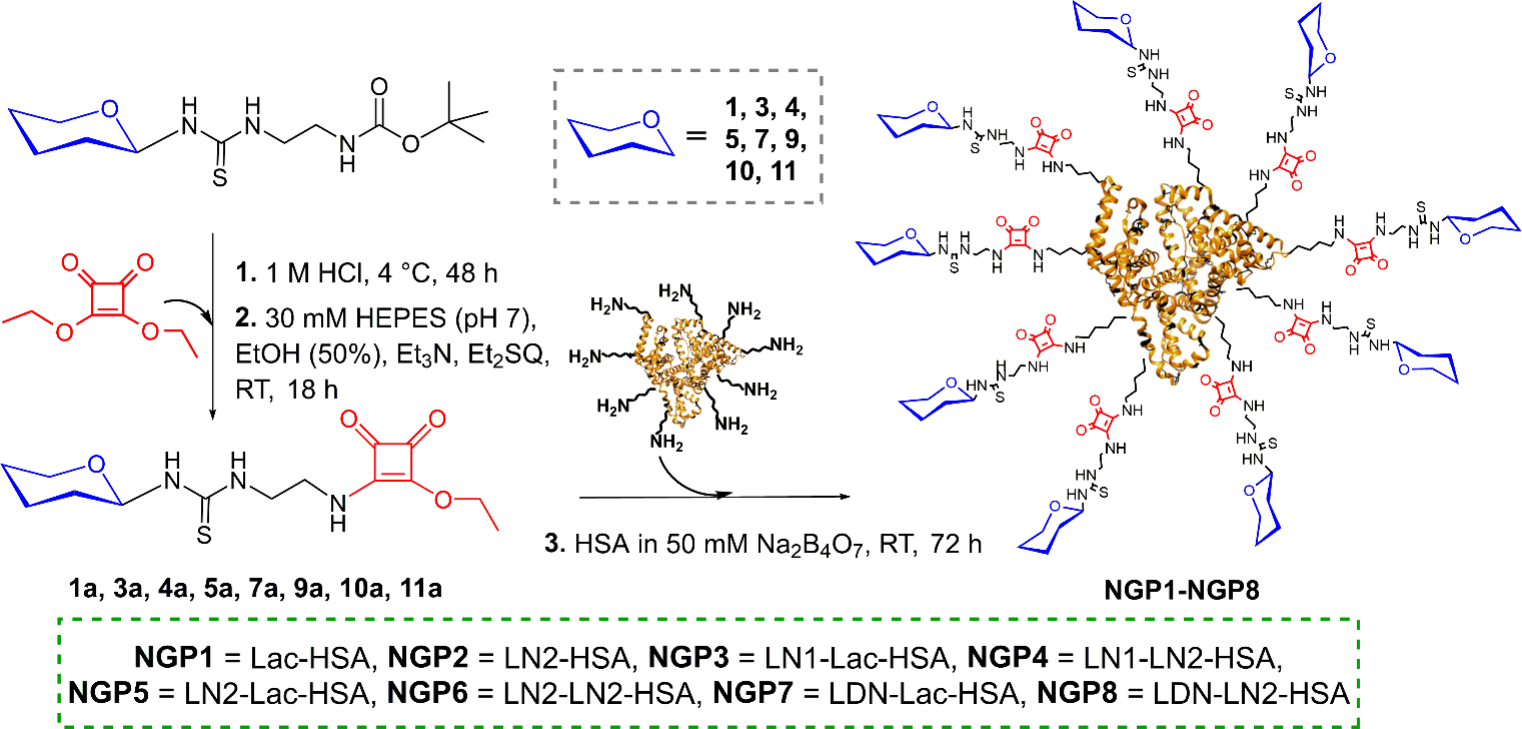
Synthesis of Neo-Glycoproteins **NGP1**–**NGP8** ^a^

a The structures of the carbohydrate moieties are schematized for clarity.

### 3.3 Production and Purification of Galectins

For the affinity determination by competitive ELISA (Enzyme-Linked ImmunoSorbent Assay), we produced His-tagged constructs of Gal-1, Gal-3, Gal-4, Gal-8, and Gal-9 in *E. coli* Rosetta 2­(DE3) pLysS, and purified them as described previously^18,24–26,34^ (Supporting Information, Section 6). For biolayer interferometry (BLI) measurements requiring galectin immobilization, N-terminal AVI-tagged galectin constructs were used. They were expressed in *E. coli* BL21­(λDE3) cells cotransformed with the *birA* gene encoding for biotin ligase, which *in vivo* biotinylates the AVI-tag sequence (GLNDIFEAQ**K**IEWHE) of the galectin construct with a single lysine residue. Whereas Gal-3-AVI could conveniently be AVI-tagged at the flexible N-terminal domain, the introduction of an AVI-tag directly at either terminus of the other (globular) galectins resulted in a substantial loss of lectin activity when immobilized on a biosensor.^34^ This loss of binding affinity was probably caused by steric hindrance as the carbohydrate-binding site of Gal-1 is in proximity to the biosensor surface and interferes with glycan recognition. In our previous study, we designed a Gal-1 construct (Gal-1-AVI-link), in which the AVI-tag was separated from the protein by a soluble neutral 15-amino-acid peptide linker consisting of five GGS repeats - (GGS)_5_. Its design was based on the work by Chen et al., who investigated the effect of the linker type and length on the protein function.^50^ Glycine as the smallest and neutral amino acid contributes to minimal steric hindrance while serine adds hydrophilicity. The GGS (Gly-Gly-Ser) repeat thus acts as a flexible hydrophilic linker that preserves lectin activity and conformational freedom.^20^ For the tandem-repeat galectins, we used the same design of the gene constructs, and prepared Gal-8-AVI-link and Gal-9-AVI-link by molecular biology methods as described previously.^27^ In the frame of this work, we used this strategy to prepare the AVI-tagged constructs of tandem-repeat galectin Gal-4 (Gal-4-AVI-link), and all tandem repeat galectin subunits (Gal-8N-AVI-link, Gal-8C-AVI-link, Gal-9N-AVI-link, Gal-9C-AVI-link, Gal-4N-AVI-link, and Gal-4C-AVI-link link; see Supporting Information, Section 6, for details). The gene constructs of AVI-galectins and their subunits are depicted in Supporting Information, Figure S29. The lectin activity of the AVI-tagged constructs was verified by ELISA and matched the respective His-tagged controls (Supporting Information, Table S8).

### 3.4 Affinity Studies with Full-Length Galectins by ELISA

The initial screening of the prepared carbohydrate library and their multivalent conjugates, neo-glycoproteins (NGPs) based on human serum albumin, was performed to evaluate their interactions with cancer-related tandem-repeat galectins using an ELISA assay.^27,34^ A competitive inhibition assay was used to simulate physiological conditions and the competitive binding environment of the cell surface glycocalyx. Serially diluted ligands and NGPs were incubated with individual galectin species in the wells coated with asialofetuin (ASF). The remaining galectin bound to the immobilized ASF was detected using an anti-His_6_ antibody. Monovalent carbohydrate compounds and NGPs containing 5–9 carbohydrate units (see Supporting Information, Table S7, for detailed compositions) were evaluated for their binding efficiency to tandem-repeat galectins Gal-4, Gal-8, and Gal-9 (Table 1), and, for comparison, to the most studied prototypic Gal-1, and chimeric Gal-3 (Supporting Information, Table S9). The results showed a clear difference in the inhibitory potential between monovalent carbohydrates and their multivalent counterparts. Overall, the highest multivalency effect was achieved for Gal-4, where the binding of monovalent ligands was relatively weak compared with the other two tandem-repeat galectins. In contrast, for Gal-8 and Gal-9, the affinity to monovalent ligands (**4**, **5**, **10**, **11**) was higher, with a slightly smaller contribution of the multivalency effect. Notably, the most striking enhancement was observed for the **LN1-LN2** (**9**) motif and its multivalent **NGP** conjugate. Multivalency resulted in a remarkable 10 800-fold increase in affinity to Gal-4 (1800-fold per one bound **LN1-LN2** epitope), as evidenced by a decrease in IC_50_ from 2380 μM for **LN1-LN2** (**9**) to 0.22 μM for **NGP4** and more than 8000-fold increase in affinity to Gal-8 (1300-fold per one **LN1-LN2** epitope), reducing IC_50_ from over 5000 μM for monovalent **LN1**-**LN2** (**9**) motif to 0.6 μM for respective **NGP4**. Similarly, highly scoring multivalency was observed in the case of **Lac** (**1**) and its **NGP1** conjugate with a 13 200-fold increase in affinity to Gal-4 (1400-fold per **Lac** epitope), with IC_50_ values decreasing from 2520 μM to 0.19 μM. A strong multivalent effect in Gal-8 binding was also observed for **LDN-LN2** (**11**) motif and its **NGP8** conjugate, exhibiting a 1600-fold improvement in affinity (180-fold per one bound **LDN-LN2** epitope), reducing IC_50_ from 270 μM (**11**) to 0.17 μM (**NGP8**). Gal-9 showed a comparable response to multivalency, with **Lac** motif and **NGP1** achieving a 2000-fold increase in affinity (210-fold per **Lac** epitope) as IC_50_ values shifted from 2180 μM (monovalent **Lac**) to 1.1 μM (**NGP1**). Similarly, **LN2** (**7**) motif and **NGP2** displayed a 1700-fold improvement (270-fold per one bound **LN2** epitope), with IC_50_ values of 2840 μM and 1.7 μM, respectively. In addition to the dramatic increases in affinity, several NGPs also demonstrated robust binding across multiple galectin types, achieving submicromolar affinities (especially **NGP5**, **NGP6**, and **NGP7**). The ultimately best binders of each galectin in the mono- and multivalent presentation can be identified from Table 1; however, the declared differences between several strongly binding ligands were generally not huge and were often within the error of measurement. Therefore, based on the data in Table 1, we identified the **LN2-Lac** (**4**) motif and its multivalent conjugate **NGP5** as the most robust strong binders, which displayed the best overall affinity across all galectins among the tested compounds. This pair of ligands was selected for further investigation of binding patterns and structural differences using NMR and biolayer interferometry (BLI) and compared to the standard ligand **Lac** and its multivalent conjugate **NGP1**.

**Table 1:** Affinity (IC_50_) of Prepared Compounds to Gal-4, Gal-8, and Gal-9 Determined by Competitive ELISA Assay

		IC_50_ ^a^ [μM]					
Compound	Sample	Gal-4	rp^c^	Gal-8	rp^c^	Gal-9	rp^c^
**1**	Lac	2520 ± 180^b^	-	2870 ± 470	-	2180 ± 670	-
**NGP1**	Lac-HSA	0.19 ± 0.02^b^	13,158	>20	<143	1.1 ± 0.2	1985
**7**	LN2	8630 ± 530	-	18,700 ± 7100	-	2840 ± 650	-
**NGP2**	LN2-HSA	1.7 ± 0.2	5075	>20	<435	1.7 ± 0.3	1671
**3**	LN1-Lac	108 ± 16	-	820 ± 140	-	230 ± 43	-
**NGP3**	LN1-Lac-HSA	0.30 ± 0.07	360	2.9 ± 0.2	183	0.28 ± 0.02	920
**9**	LN1-LN2	2380 ± 670	-	>5000	-	>5000	-
**NGP4**	LN1-LN2-HSA	0.22 ± 0.05	10,814	0.6 ± 0.2	>8000	4.9 ± 1.9	>1020
**4**	LN2-Lac	620 ± 100^b^	-	130 ± 30	-	168 ± 55	-
**NGP5**	LN2-Lac-HSA	0.19 ± 0.05	3158	0.20 ± 0.01	650	0.16 ± 0.03	1050
**10**	LN2-LN2	2390 ± 490	-	406 ± 46	-	52 ± 23	-
**NGP6**	LN2-LN2-HSA	0.48 ± 0.16	4975	0.42 ± 0.12	967	0.17 ± 0.03	306
**5**	LDN-Lac	1620 ± 260	-	75 ± 9	-	326 ± 26	-
**NGP7**	LDN-Lac-HSA	0.38 ± 0.11	4268	0.40 ± 0.14	134	0.58 ± 0.08	627
**11**	LDN-LN2	2010 ± 1000	-	270 ± 85	-	260 ± 75	-
**NGP8**	LDN-LN2-HSA	1.6 ± 0.5	1258	0.17 ± 0.02	1588	0.20 ± 0.01	1300

a IC_50_ (half maximal inhibitory potency) is the concentration of compound required to inhibit galectin binding to immobilized ASF by 50%. Each value was determined in at least quadruplicate.

b Previously published by us.^24^

c Relative potency (rp) was calculated as the ratio of the affinity of monovalent ligand and its respective neo-glycoprotein.

### 3.5 Structural Insights into the Binding Events by Nuclear Magnetic Resonance (NMR)

Nuclear magnetic resonance (NMR) is widely used to investigate protein-carbohydrate interactions.^51^ Different NMR methods provide critical insights into the involvement of specific epitopes in these interactions^52^ that can be employed to deduce the three-dimensional structures of proteins, glycans, and their complexes. Given the unique chemical and dynamic properties of glycans, NMR is often the preferred technique for analyzing their structures, conformations, and interactions with biomolecular receptors.^51,52^ NMR methods used to analyze interactions are typically classified into two categories. In ligand-based approaches, the changes in the NMR parameters of the ligand (in this case, the glycan or neo-glycoprotein) upon binding to the receptor (lectin) are exploited by diverse NMR strategies. Alternatively, in the receptor-based approaches, alterations in the NMR signals of galectin are monitored.^33^ Herein we have employed both approaches to unravel specific features of the molecular recognition event. Using saturation-transfer difference (STD-NMR) experiments, we have mapped the binding epitopes for the best-binding ligand motif **LN2-Lac** (**4**). Additionally, two-dimensional ^1^H–^15^N heteronuclear single quantum coherence (HSQC) experiments have been employed to monitor the chemical shift perturbations in the backbone amide signals of the galectin at the amino acid level upon its interaction with the **NGP5**, which present the **LN2-Lac** (**4**) motif in a multivalent arrangement. Therefore, information on the binding epitope at the oligosaccharide level as well as at the particular binding site of the galectin that drives the interaction with the multivalent ligand was deduced. Moreover, the analysis of the results provided by these methods enabled us to experimentally prove the existence of different ligand/lectin complexes.

#### 3.5.1 Saturation-Transfer Difference (STD) Experiment

Saturation-transfer difference (STD-NMR) is a well-established methodology widely used for analyzing protein–ligand interactions,^53^ frequently in combination with computational methods, such as molecular dynamics simulations.^37^ The analysis of the STD-NMR spectra provides atomic-level information on the ligand protons that are in the proximity to the protein receptor within the binding complex, thus defining the ligand epitope. STD-NMR is best applicable to small to medium-sized ligands that display a relatively fast dissociation rate constant (*k*_off_)^54^ in the relaxation time scale. Thus, in our case, the glycan only, rather than the whole neo-glycoprotein, was used in the STD-NMR experiments, to define the epitope for the interaction of **LN2-Lac** (**4**) with Gal-4, Gal-8, and Gal-9. Before the STD-NMR analysis, 2D NMR homo- (^1^H,^1^H-TOSCY, ^1^H,^1^H-NOESY) and heteronuclear (^1^H,^13^C-HSQC) correlation spectra of **LN2-Lac**-*t*Boc ligand (**4**) were acquired and analyzed to provide the full ^1^H assignment (see Supporting Information, Figure S31).

Interestingly, in the ^1^H NMR spectrum of **LN2-Lac** (**4**), the signals of the protons of the Glc residue were significantly broadened, probably due to a dynamic equilibrium of conformers of the thiourea linker at position C1. Therefore, this Glc residue was not considered for a detailed STD-NMR analysis. The relative STD values for the respective proton signals were calculated as described previously^33^ and expressed as an epitope map (Figures 2B, D, E). For Gal-8 and Gal-9, the resulting STD-NMR spectra were similar (Figures 2C and S32), suggesting the existence of comparable binding epitopes. In both cases, the strongest STD value corresponded to the protons at the C-6 position (H-6, H-6′) of the *N*-acetylglucosamine (GlcNAc) unit together with H-4 and H-6 of the internal galactoside (Gal^INT^) unit, therefore defining the internal -GlcNAcβ3Galβ- moiety as the key epitope for these galectins. Additional STD intensities were also observed for protons of Gal^TER^, although of weaker intensity compared to those of Gal^INT^, indicating that this terminal residue is an additional binding epitope. The protons of the *N*-acetyl groups exhibited a weak STD effect in both cases, consistently with the previously published crystal structures of Gal-8N^55^ and Gal-9N,^40^ where the *N*-acetyl groups were positioned outside the binding pocket. This fact was also confirmed by *in silico* docking of ligands and by molecular dynamics simulation (Section 3.7) where the *N*-acetyl group of the **LN2-Lac** (**4**) motif docked in the binding sites of Gal-4, -8, or -9 was exposed to water and its methyl group did not form any direct interactions with the galectin during molecular dynamics simulation, forming only mediated interactions. The oxygen of the *N*-acetyl group can rarely form hydrogen bonds with Arg221 (Gal-9C), Arg44 (Gal-9N), Arg45 (Gal-8N), or Arg45 (Gal-4N) as seen in Supporting Information, Tables S10–S12. A similar correlation with the published crystal structures was observed for the protons of the reducing-end glucose residue, particularly H-3. While H-3 is in the proximity to the binding cavity, H-6 engages in hydrogen bonding mediated by water molecules. Similarly, in the reducing-end GlcNAc residue, its H-3 and H-4 protons are exposed to water.

**1:**
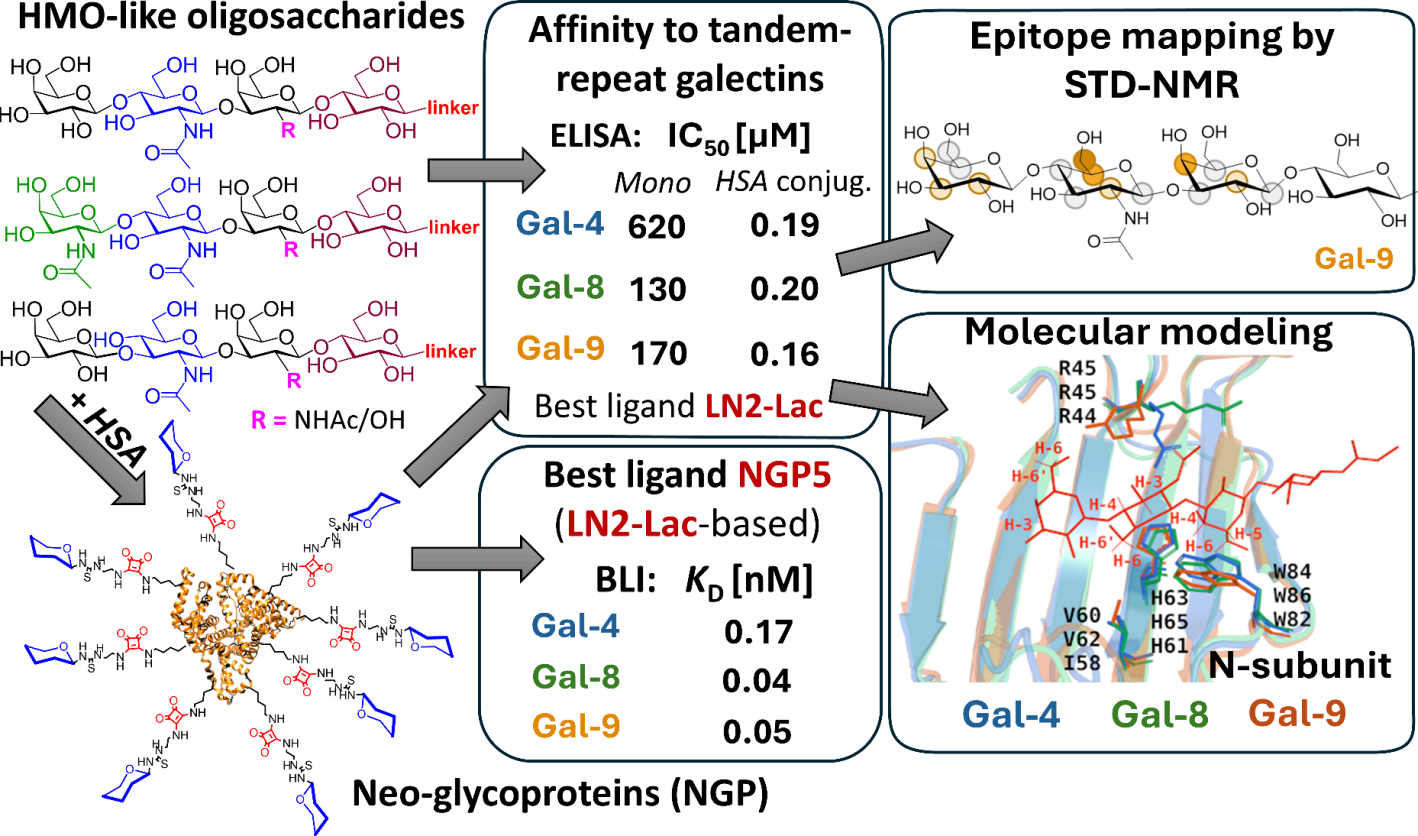
Overall strategy of the work. The best-binding tetrasaccharide motif **LN2-Lac** had micromolar affinity in monovalent and submicromolar affinity in multivalent presentation on HSA. Important ligand epitopes (middle panel) are predicted to interact with crucial amino acid residues (right panel) in each galectin.

Interestingly, in the case of the interaction with Gal-4, the ^1^H NMR spectrum of **LN2-Lac** (**4**) in the presence of galectin (50:1 ratio), showed a severe signal line broadening, selective for protons of the internal Gal moiety, as well as H-6 and H-6′ of the linked GlcNAc moiety (Figure 2A). These specific line-broadening effects resulted from an enhanced transverse relaxation of these protons in the bound state, a phenomenon that is probably caused by specific ligand–receptor exchange processes that mainly involve those residues.^56,57^ This experimental observation points out that this Gal residue is an important binding epitope for this galectin-ligand system. At the same time, the STD-NMR spectrum (Figure 2A) unambiguously showed a very strong STD effect for protons H-4, H-3, and H-2 of the terminal galactose residue (Gal^TER^). Obviously, no STD effect was observed for protons of Gal^INT^, since they are too weak due to the severe line broadening. Under these circumstances, in which the ligand signal intensities are biased due to line broadening, STD cannot be used for epitope mapping. In any case, all together, the line broadening and the STD observations strongly suggest that both the internal and terminal Gal moieties are involved in the interaction with the two Gal-4 CRDs, generating multiple ligand-galectin complexes in dynamic exchange.

**Figure 2:**
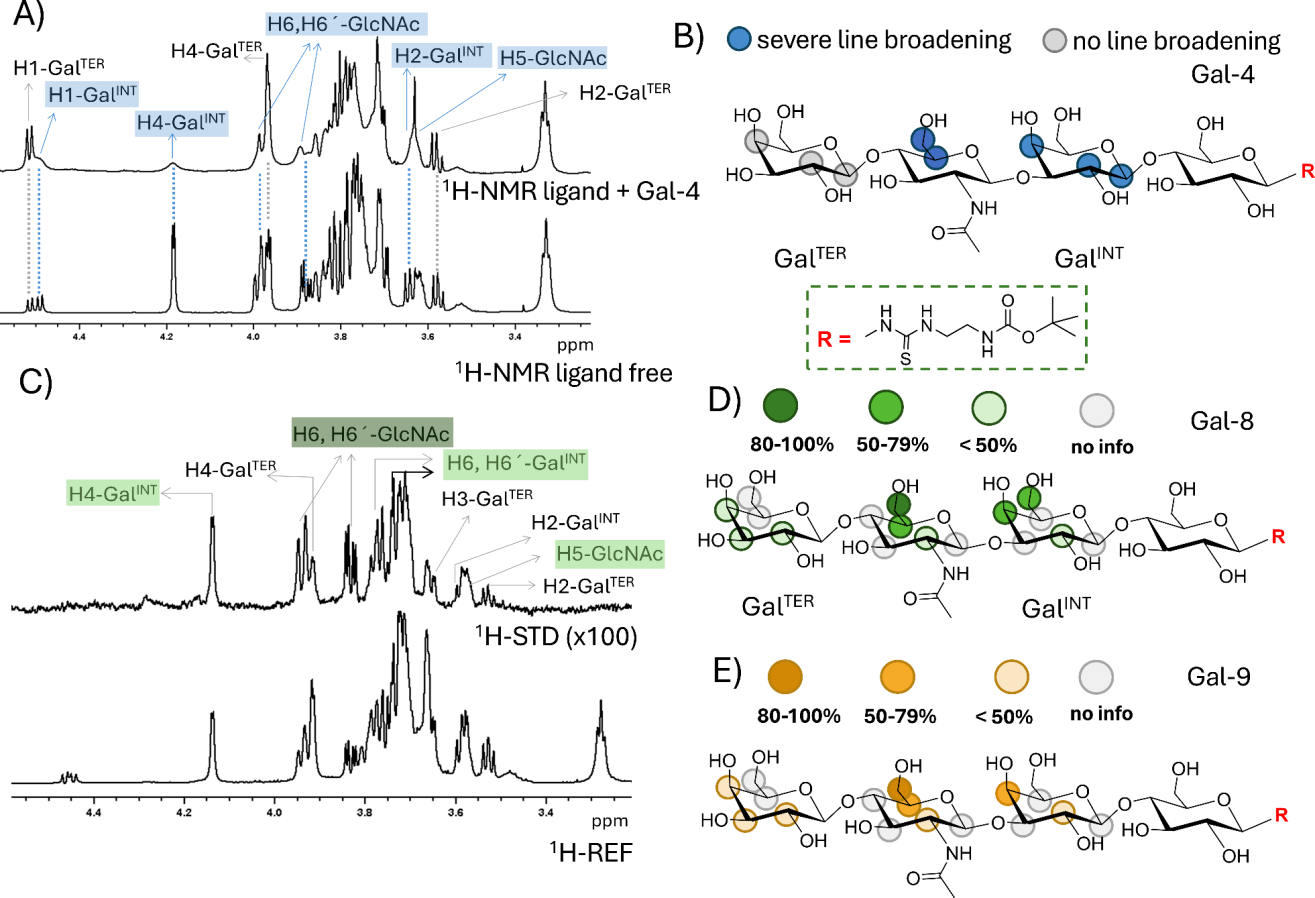
(A) Saturation-transfer difference (STD-NMR) experiment used for epitope mapping with Gal-4, acquired on a sample of Gal-4 (40 μM) + **LN2-Lac**-*t*Boc (2 mM) solution (50:1 ligand-to-galectin ratio). Upper spectrum: STD-NMR spectrum, derived from the subtraction of the off-resonance and on-resonance spectra. Lower spectrum: off-resonance spectrum (reference). Some ^1^H-signals are annotated: those experiencing strong line broadening are indicated in blue, while those not affected have colorless labels. (B) Epitope map of the **LN2-Lac** motif with Gal-4 according to selective line broadening. (C) Saturation-transfer difference (STD-NMR) experiment used for epitope mapping with Gal-8, acquired on a sample of Gal-8 (40 μM) + **LN2-Lac**-*t*Boc (2 mM) solution (50:1 ligand-to-galectin ratio). Upper spectrum: STD-NMR spectrum, derived from subtraction of the off-resonance and on-resonance spectra. Lower spectrum: off-resonance spectrum (reference). Some ^1^H-signals showing significant STD effects are annotated depending on their STD intensity: dark green, strongest STD; middle green, strong STD; colorless, weak STD. (D, E) Epitope maps of the **LN2-Lac** motif based on relative STD values in interaction with (D) Gal-8 or (E) Gal-9.

#### 3.5.2 ^1^H–^15^N Heteronuclear Single Quantum Coherence (HSQC) Experiment

To assess the molecular recognition events from the protein perspective, we opted for monitoring the perturbations of the amide backbone NMR signals by recording ^1^H–^15^N heteronuclear single quantum coherence (HSQC) experiments. The production of ^15^N-labeled proteins, essential for conducting these HSQC experiments, is described in Supporting Information, Section 6. In this case, the synthetic neo-glycoproteins were employed as titrating ligands.

Titrations of the ^15^N-labeled full-length galectins Gal-4, Gal-8, and Gal-9 with 0.6, 1.1, or 1.6 molar equiv (per oligosaccharide) of neo-glycoproteins **NGP1** and **NGP5** were performed. The ^1^H–^15^N cross peaks corresponding to the protein backbone were initially assigned based on the previously published backbone assignments for the individual subunits.^36,37^ The NMR assignment of Gal-9, which has not yet been reported, is available from the authors upon request. Due to the high molecular weight of the multivalent NGPs and, obviously, of the supramolecular complexes generated, no chemical shift perturbations (CSPs) were observed. Instead, a dramatic loss of signal intensity was detected, as expected for the enhanced transverse relaxation rates due to the combination of the large size of the complexes and the chemical exchange events. Therefore, our focus was concentrated on monitoring the cross-peak signal intensity decrease in the HSQC spectra of the lectin upon adding **NGP1** or **NGP5**, using them as a proxy to identify the ligand-binding sites on the dimeric proteins. A comparative analysis of the spectra acquired in the presence of **NGP1** and **NGP5** neo-glycoproteins revealed that the **LN2** motif showed a strong preference for the N-subunits of all tested galectins (see Supporting Information, Figures S33B, S34B, S35B). In contrast, the **Lac** motif exhibited a more balanced quenching of the cross-peak signals across subunits (see Figures S33A, S34A, S35A). For Gal-4, a substantial decrease in the signal intensity was observed for both **NGP1** and **NGP5** upon the addition of 1.6 equiv of glycan epitopes relative to the protein concentration. Notably, a partial decrease in the signal intensities was already apparent upon the addition of 0.6 equiv of **NGP5**, particularly for those amino acids at the N-domain, with a nearly complete signal loss at 1.6 equiv of these N-domain signals. Fittingly, these results align closely with the affinities determined by ELISA (Table 1). Titration experiments of Gal-9 exhibited strong cross-peak quenching and a slight preference for the N-subunit after the initial titration with both **NGP1** and **NGP5**. However, due to the high affinity of the neo-glycoproteins, subsequent titrations led to a complete loss of detectable cross-peak intensities, reducing them to nearly zero. In contrast, for Gal-8, the decrease in signal intensity was significantly less pronounced following the addition of 1.6 equiv of **NGP1** whereas **NGP5** induced a substantial decrease in signal intensity. To demonstrate the overall binding potency of the neo-glycoproteins, the total relative intensities were computed to express their affinity to the entire protein (Figure 3). These findings underscore the differential binding patterns and affinities of galectins to multivalent ligands and provide further validation of the trends following from the ELISA measurement at a high resolution.

**Figure 3:**
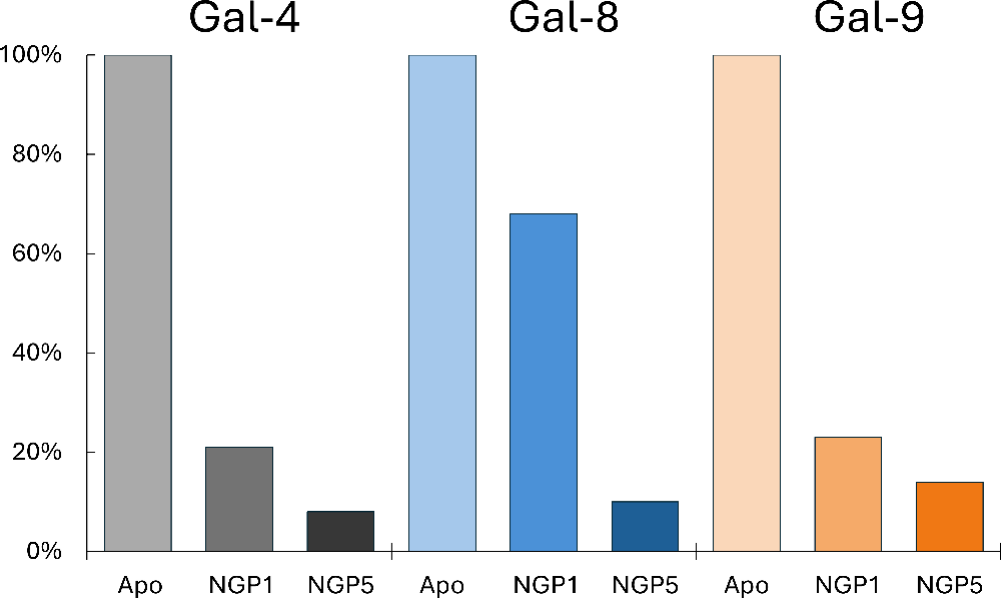
Total relative intensities of ^1^H–^15^N HSQC experiments of galectins with **NGP1** or **NGP5**. Relative intensities are computed as an average volume of cross-peaks after neo-glycoprotein addition (1.6 equiv of carbohydrate epitope) divided by an average volume of cross-peaks of galectins in the apo state.

Therefore, the NMR results provide information on the key epitope at the glycan level as well as demonstrate the existence of multiple complexes in equilibrium, although with specific preferences at the domain level, depending on the galectin, which are also able to discriminate between the two neo-glycoproteins.

### 3.6 Affinity Studies by Biolayer Interferometry (BLI)

Biolayer interferometry (BLI) was employed as a complementary method to confirm the high affinities and gain deeper insights into the kinetics of the interactions between NGPs and galectins—both full-length and their respective subunits. Analogously to the NMR experiments (Section 2.5), the overall best-performing ligand motif **LN2-Lac** (**4**) and its neo-glycoprotein **NGP5** were analyzed along with the reference ligand **Lac** (**1**) and its multivalent conjugate **NGP1**. Monobiotinylated galectin constructs were immobilized on streptavidin-coated biosensors using the Octet RED96e device. Scout experiments were performed prior to each measurement to ensure accuracy and to determine the lowest loading concentration that elicited a sufficient response. The binding curves were analyzed using a partially fitted 1:1 Langmuir model. To further investigate potential secondary phenomena, such as rebinding or the docking of one NGP molecule to two or more immobilized galectin constructs, kinetic interactions were re-evaluated using alternative models. A 1:2 kinetic model was applied for all subunits, while a 2:1 kinetic model was used for full-length proteins. These adjustments ensured that the data were not overestimated to avoid artificially inflated affinities that could misrepresent the kinetic parameters and the described interaction.

Notable trends emerged from the data sets obtained (Table 2). For **Lac**-decorated **NGP1**, the Lac epitope exhibited similar binding affinities to both the N-subunit and the C-subunit of all galectins except for Gal-9, where a marked difference was observed (*K*_D_ = 40.7 nM for Gal-9N vs. *K*_D_ = 237 nM for Gal-9C). This difference was largely driven by the dissociation rate, which was almost 7-fold slower for the N-subunit, suggesting that the **NGP1**-Gal-9C complex decayed significantly faster than the **NGP1**-Gal-9N complex. Despite these differences, *K*_D_ values for all tested galectins remained in the subnanomolar range. For **NGP5** decorated with **LN2-Lac** (**4**), a clear preference for the N-subunit was observed, especially in Gal-8 and Gal-9; the dissociation rates played a crucial role in this behavior. The clearest difference was observed between Gal-8N and Gal-8C, where the dissociation rates were 0.53 × 10^–3^ 1/s and 22 × 10^–3^ 1/s respectively, resulting in a 42-fold faster decay of the complex and a corresponding 42-fold higher *K*_D_ value for Gal-8C (*K*_D,Gal‑8N_ = 2.4 nM vs. *K*_D,Gal‑8C_ = 102 nM).

**Table 2:** Kinetics of Interaction of **NGP1** or **NGP5** with Gal-4, Gal-8, and Gal-9 and Their Respective Subunits Determined by BLI

Neo-glycoprotein			Gal-4	Gal-8	Gal-9
**NGP1** (**Lac**-HSA)	N-subunit	*k*_a_ ^a^	2.23 ± 0.05	3.27 ± 0.08	0.96 ± 0.04
		*k*_d_ ^b^	11.3 ± 0.9	12.7 ± 0.9	3.9 ± 0.2
		***K***_**D**_ ^c^	**50.5 ± 6.3**	**39.9 ± 2.3**	**40.7 ± 1.9**
	C-subunit	*k*_a_ ^a^	2.16 ± 0.02	2.07 ± 0.06	1.13 ± 0.07
		*k*_d_ ^b^	4.62 ± 0.05	5.94 ± 0.53	26.8 ± 4.9
		***K***_**D**_ ^c^	**21.4 ± 0.5**	**28.7 ± 0.9**	**237 ± 43**
	full-length galectin	*k*_a_ ^a^	4.83 ± 0.11	6.11 ± 0.02	7.16 ± 0.07
		*k*_d_ ^b^	0.3 ± 0.01	0.18 ± 0.07	0.24 ± 0.05
		***K***_**D**_ ^c^	**0.62 ± 0.01**	**0.29 ± 0.01**	**0.34 ± 0.01**
**NGP5** (**LN2-Lac**-HSA)	N-subunit	*k*_a_ ^a^	2.73 ± 0.02	2.22 ± 0.04	1.36 ± 0.16
		*k*_d_ ^b^	17.5 ± 3.5	0.53 ± 0.04	0.94 ± 0.05
		***K***_**D**_ ^c^	**64.1 ± 1.9**	**2.4 ± 0.3**	**6.9 ± 0.3**
	C-subunit	*k*_a_ ^a^	1.41 ± 0.01	2.15 ± 0.02	1.6 ± 0.1
		*k*_d_ ^b^	11.4 ± 0.1	22.1 ± 3.4	32.2 ± 1.4
		***K***_**D**_ ^c^	**80.8 ± 4.6**	**102 ± 16**	**202 ± 45**
	full-length galectin	*k*_a_ ^a^	9.39 ± 0.21	21.7 ± 0.7	31.6 ± 0.8
		*k*_d_ ^b^	0.16 ± 0.02	0.09 ± 0.01	0.14 ± 0.01
		***K***_**D**_ ^c^	**0.17 ± 0.01**	**0.04 ± 0.01**	**0.05 ± 0.01**

a *k*_a_ × 10^5^ [L/mol/s].

b *k*_d_ × 10^–3^ [1/s].

c *K*_D_ [nM].

Similar trends were observed when comparing **LN2-Lac**-decorated **NGP5** with **Lac**-decorated **NGP1**; a 16-fold improvement in affinity was found in Gal-8N (*cf. K*_D_ = 39.9 nM to *K*_D_ = 2.4 nM, respectively) due to a 24-fold decrease in the dissociation rate of the complex. In contrast, the affinity of Gal-8C decreased more than 3-fold between **NGP1** and **NGP5**, shifting from *K*_D_ = 28.7 nM to *K*_D_ = 102 nM. Even greater differences were observed between Gal-9 subunits. The dissociation rate for Gal-9N decreased from *k*_d_ = 3.9 × 10^–3^ L/mol/s for **NGP1** to 0.94 × 10^–3^ L/mol/s for **NGP5**; in contrast, no significant difference between the kinetic parameters of **NGP1** and **NGP5** was observed in Gal-9C. These data emphasize the leading affinity of the N-subunit for the **LN2-Lac**-based motif.

### 3.7 Molecular Modeling

The interactions of all monovalent carbohydrate ligands used in this work (**1**, **3**, **4**, **5**, **7**, **9**, **10**, **11**) with both subunits of the studied tandem-repeat galectins (Gal-4N, Gal-4C, Gal-8N, Gal-8C, Gal-9N, Gal-9C) were analyzed *in silico* based on docking and molecular dynamics simulation.

The free energies of binding for the respective carbohydrate-galectin complexes were calculated by Autodock Vina for selected snapshots of molecular dynamics simulation and are shown in Table 3. For better visualization, a graphical presentation of the free energy data is included in Supporting Information, Figure S39. Tetrasaccharide ligands clearly show higher affinities than disaccharide ligands in all subunits, which is in good agreement with the measured IC_50_ values. In Gal-4 there is a clear preference for ligand binding in the N-subunit whereas for Gal-8 and -9 this difference is more ligand-dependent.

**Table 3:** Free Energies of Binding of Ligands to Galectin Subunits (a Lower Value Indicates Better Binding)^a^

	Free energy of binding [kcal/mol]					
Ligand	Gal-4N	Gal-4C	Gal-8N	Gal-8C	Gal-9N	Gal-9C
**Lac** (**1**)	–3.98	–3.60	–3.94	–4.08	–3.68	–4.03
**LN1-Lac** (**3**)	–5.14	–4.10	–4.23	–3.45	–4.94	–5.33
**LN1-LN2** (**9**)	–4.47	–3.77^b^	–5.12	–4.15	–4.74	–4.95
**LN2** (**7**)	–4.19	–3.58	–4.10	–4.03	–4.27	–2.98
**LN2-Lac** (**4**)	–5.38	–3.87	–5.34	–5.08	–4.75	–4.36
**LN2-LN2** (**10**)	–5.62	–3.54	–4.97	–4.97	–5.44	–4.83
**LDN-Lac** (**5**)	–5.18	–3.70	–6.08	–4.83	–5.69	–5.17
**LDN-LN2** (**11**)	–5.09	–3.82	–5.91	–3.39	–5.49	–5.17

a Data averaged from 15 snapshots of a stable period of molecular dynamics simulation (80–100 ns).

b The ligand got reoriented during molecular dynamics simulation into a distinct position from other ligands (data for 80–100 ns; Supporting Information, Figure S40).

The frequencies of hydrogen bonding interactions formed by individual carbohydrate ligands are listed in Supporting Information, Tables S10–S12. Active site interactions are visualized in Supporting Information, Figures S40–S42. The interactions of the best-binding universal ligand, **LN2-Lac** (**4**), are detailed in Figure 4.

**Figure 4:**
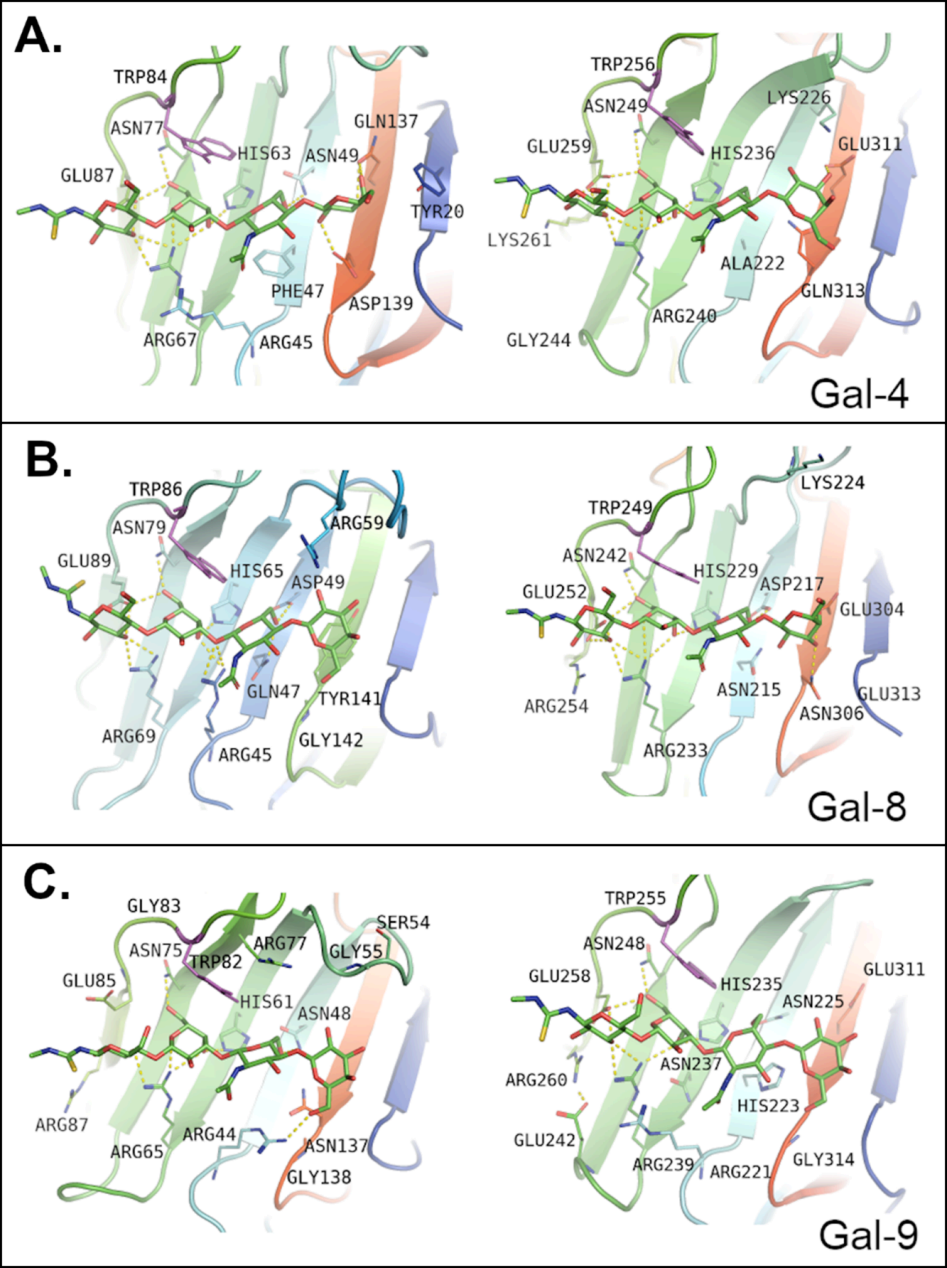
Interactions formed by **LN2-Lac** in the active sites of galectins. Amino acid residues able to form hydrogen bonds with carbohydrates are shown and labeled; nonpolar hydrogens are hidden. Trp residues forming CH−π stacking interactions are shown and colored in magenta. The top figure depicts the binding into the N-subunit, and the bottom figure, into the C-subunit of (A) Gal-4, (B) Gal-8, and (C) Gal-9. A slight rotation occurred in the non-reducing-end units of the ligand in Gal-8C as a result of a stronger interaction with the active site residues; otherwise, the ligand orientations are generally similar. Slightly different angles of view may have been selected to better depict respective hydrogen bonds.

A detailed analysis of the results of docking and molecular dynamics simulations (Supporting Information, Section 9) revealed distinct preferences of the respective galectin CRDs for the structural characteristics of each ligand, which could explain varying ligand affinities from the structural point of view.

In Gal-4N, the β(1→4)-linked **LN2** motif binds stronger in the A–B site than the β(1→3)-linked **LN1** motif due to its stabilization by hydrogen bonding with Arg45 and stacking CH−π with Phe47. The CH−π stacking interaction is by far not a negligible force; rather, it plays a crucial role in saccharide recognition and is essential for the design of putative ligands.^58,59^ Furthermore, Gal-4C binds *N*-acetylated carbohydrates in the C–D site weaker than **Lac** because the C-2 *N*-acetyl group shows a weaker interaction with Lys261. In general, Gal-4N has a stronger ligand affinity. A more detailed description of the structure-affinity relations is shown in Supporting Information, Section 9.

In Gal-8, hydrophobic contacts play a key role in the stabilization of the *N*-acetylated ligands in the A–B site. Again, **LN1** is bound to Gal-8 more weakly than **LN2**, with a slightly better binding score for Gal-8N due to the interaction with Arg59 of the B–C loop. This residue also improves interactions of Gal-8N with **LDN**-capped substrates. Arg254 forms weak van der Waals interaction with the *N*-acetyl group of **LN2** in the C–D site (Supporting Information, Figure S45). The position of **LN2** in the A–B site of Gal-8C is improved by hydrophobic interactions, and in the A–B site of Gal-8N by CH−π stacking with Tyr141.

In Gal-9 we observed more straightforward ligand-dependent preferences. Gal-9N prefers **LN2** substrates (both in the A–B and C–D sites) while Gal-9C prefers **Lac** in the C–D site and **LN1** in the A–B site. **LN2** interacts with Arg87 in Gal-9N, but not with analogous Arg260 in Gal-9C (Supporting Information, Figure S45). The analysis of the complexes allowed us to conclude that both arginines have different orientations, resulting from the interaction with other amino acid residues (namely Glu242 of Gal-9C). This highlights the fact that the mere presence of a similar residue does not always provide similar interactions and affinities. Similarly to Gal-8N, hydrophobic contacts play an important role in the binding of **LDN**-capped substrates in Gal-9.

The best-binding universal ligand, **LN2-Lac** (**4**), exhibits additional interaction between the H-6 of its reducing-end GlcNAc unit with Gal-8: here, the H-6/H-6′ atom does not form a hydrogen bond with any residue of Gal-8, but they form hydrophobic contacts with Trp86 and Val62 in the N-subunit, or with Trp249 and Ile226 in the C-subunit. The H-4 proton of the internal galactose unit is placed close to the galectin binding groove and C-4 hydroxyl forms hydrogen bonding with the conserved His residues in the N- and C-subunits of galectin CRDs (His63 or His236, respectively, in Gal-4; His65 or His229, respectively, in Gal-8; His61 or His235, respectively, in Gal-9).^27^ Furthermore, in the interaction with Gal-4, the H-5 and H-4 proton of the internal galactose unit is pointed toward Trp84 (Gal-4N) or Trp256 (Gal-4C) and forms hydrophobic interactions with these residues. This residue is conserved across all studied galectins (Trp86 or Trp249 in Gal-8; Trp82, or Trp255 in Gal-9).

## 4 Conclusion

The present work pioneers the development of complex multivalent ligands of tandem-repeat galectins, which are crucial for further studies on the pharmacological inhibition of this still poorly understood galectin family as well as for the development of new diagnostic tools. In addition to poly-LacNAc ligands, a selection of neutral human milk oligosaccharides was synthesized and analyzed. Structure-affinity relationships were formulated with respect to the selectivity and/or the versatility of the prepared neo-glycoprotein ligands for this galectin family. The synergy of the detailed interaction data by ELISA, biolayer interferometry, and nuclear magnetic resonance was supported by the conclusions of molecular modeling experiments and revealed a complex picture of the binding parameters of this important group of nature-derived ligands to tandem-repeat galectins. Therapeutic inhibition of tandem-repeat galectins is important for future fight against cancerogenesis, autoimmune disorders, or development of allergies.

## Supplementary Material



## Notes

Additional data on materials, analytical methods, enzymes for glycan synthesis, chemoenzymatic synthesis of functionalized glycans, structural analysis of prepared glycans and neo-glycoproteins, cloning and production of galectins, and molecular modeling (PDF)
