# Supplementary material for: Human Milk Oligosaccharides Multivalently Presented on Defined Synthetic Neo-Glycoproteins Are Nanomolar Ligands of Tandem-Repeat Galectins
Source: Biomacromolecules. 2025 Jul 7;26(8):4995–5009. doi: 10.1021/acs.biomac.5c00377 (PMC12344693; doi:10.1021/acs.biomac.5c00377)
Supplement: Supplementary file 1 [file bm5c00377_si_001.pdf]

Supporting Information

Human milk oligosaccharides multivalently presented on defined synthetic neo-glycoproteins are nanomolar ligands of tandem-repeat galectins

*Jakub Červený,[a,b,] Viktoria Heine,[a] Michaela Hovorková,[a,c] Petr Brož,[a] Eliška Filipová,[a,d] Natalia Kulik,[a] Martin Hubálek,[e] Josef Cvačka,[e] Lucie Petrásková,[a] Mirane Florencio-Zabaleta,[g] Sandra Delgado,[g] Helena Pelantová,[a] Zuzana Bosáková,[b] Lothar Elling,[f] Jesus Jiménez-Barbero,[g,h,i,j] Ana Ardá,[g,h] Vladimír Křen,[a] Pavla Bojarová[a]**

[a] Institute of Microbiology of the Czech Academy of Sciences, Vídeňská 1083, CZ-142 00, Prague 4, Czech Republic

[b] Department of Analytical Chemistry, Faculty of Science, Charles University, Hlavova 8, CZ-128 43, Prague 2, Czech Republic

[c] Department of Genetics and Microbiology, Faculty of Science, Charles University, Viničná 5, CZ-128 43, Prague 2, Czech Republic

[d] Department of Biochemistry and Microbiology, University of Chemistry and Technology Prague, Technická 3, CZ-166 28 Prague 6, Czech Republic

[e] Institute of Organic Chemistry and Biochemistry of the Czech Academy of Sciences, Flemingovo náměstí 2, CZ-166 10 Prague 6, Czech Republic

[f] Institute of Biotechnology and Helmholtz Institute for Biomedical Engineering, RWTH Aachen, Pauwelstr. 20, D-52079 Aachen, Germany

[g] CICbioGUNE, Basque Research and Technology Alliance, Parque Científico Tecnológico de Bizkaia building 801A, E-48160 Derio, Spain

[h] Ikerbasque, Basque Foundation for Science, Plaza Euskadi 5, Abando. E-48009 Bilbao, Spain

[i] Department of Inorganic & Organic Chemistry, Faculty of Science and Technology, University of the Basque Country, UPV/EHU, E- 48940 Leioa, Spain

[j] Centro de Investigación Biomedica En Red de Enfermedades Respiratorias, C/ Monforte de Lemos, 3-5. Pabellón 11, Planta 0, Fuencarral-El Pardo, E-28029 Madrid, Spain

* Corresponding author

Contents

1. Materials
2. Analytical Methods
3. Enzymes for Glycan Synthesis
4. Chemoenzymatic Synthesis of Functionalized Glycans
5. Structural Analysis of Prepared Glycans and Neo-Glycoproteins
6. Cloning and Production of Galectins
7. Structural Insights into the Binding Events by Nuclear Magnetic Resonance (NMR)
8. Biolayer Interferometry
9. Molecular Modeling
10. References

1. Materials

Purification columns (HisTrap™, MBPTrap™) were purchased from GE Healthcare (Chicago, USA). Nucleotide sugars UDP-Gal, UDP-GalNAc, and UDP-GlcNAc were prepared according to a previously published procedure.[1] Dowex base-free 66 was obtained from Sigma Aldrich (St. Luis, USA). Competent *E. coli* cells were purchased from New England Biolabs (Ipswitch, USA) or Merck (Prague, Czech Republic). Chemicals for the production of enzymes and galectins were purchased from Carl Roth GmbH (Karlsruhe, Germany). Unless stated otherwise, all other chemicals and solvents were from VWR International or Sigma and were of analytical grade.

2. Analytical Methods

2.1. High-Performance Liquid Chromatography (HPLC)

HPLC analyses were performed on a Shimadzu Prominence LC analytical system consisting of a LC-20AD binary HPLC pump, DGU-20A$_3$ degasser, SIL-20ACHT cooling auto sampler, a CTO-10AS column oven, a CBM-20A system controller, and SPD-20MA diode array detector (Shimadzu, Japan). Analyses of all functionalized glycans were performed on a TSKgel Amide-80 HILIC (hydrophilic interaction chromatography) column (250 × 4.6 mm, 5 μm) preceded by a TSKgel Amide-80 Guardgel (3.2 × 15 mm, Tosoh Corp., Japan) in acetonitrile/water (4/1, *v*/*v*), with gradient elution as follows (A= acetonitrile, B = water): 22% B for 0-7 min, 22-31% B for 7-16 min, 31-22% B for 16-17 min, and 22% B for 17-22 min for column equilibration. The flow rate was 1 mL/min, temperature 25 °C, injection volume 1 μL, the detection was performed at 200 nm. All squarate monoamides were analyzed by a reversed-phase analytic MultoKrom 100-5 C18 column (250 × 4.6 mm; CS Chromatographie, Langerwehe, Germany) with H_2O/acetonitrile (85/15, *v*/*v*) as mobile phase and a flow rate of 1 mL/min.

If final purification of glycans was necessary, we used a reversed-phase analytic MultoKrom 100-5 C18 column (250 × 4.6 mm; CS Chromatographie, Langerwehe, Germany) with H_2O/acetonitrile (85/15, *v/v*) as mobile phase and a flow rate of 1 mL/min, and collected the product. Squarate monoamide reaction mixtures were purified by the same method.

2.2. Mass Spectrometry (MS) Methods

The mass of *t*Boc-modified glycans **1-11** was analyzed by high-resolution mass spectrometry (HRMS) using an LTQ Orbitrap XL hybrid mass spectrometer (Thermo Fisher Scientific, Waltham, MA, USA) equipped with an electrospray ion source. Samples were dissolved in methanol or methanol/water into the mobile phase (methanol/water 4:1, *v/v*, 100 µL/min), measured in negative (spray voltage: 5.0 kV; capillary voltage: -25 V; tube lens voltage: -125 V; capillary temperature: 275 °C) and positive (spray voltage: 5.0 kV; capillary voltage: 9 V; tube lens voltage: 150 V; capillary temperature: 275 °C) ion mode, and recorded at a resolution of 100 000.

Purified squarate monoamide esters **1a**, **3a-5a**, **7a-11a** were analyzed by ESI-MS (LCMS 2020, Shimadzu, Japan). Parameters were as follows: positive and negative mode; ESI interface voltage, 4.5 kV, - 3.5 kV; detector voltage, 1.15 kV; nebulizing gas flow, 1.5 mL/min; drying gas flow, 15 ml/min; heat block temperature, 200 °C; the temperature of desolvation line pipe, 250 °C, SCAN mode 400-1200 *m*/*z*; all spectra were analyzed using software LabSolutions ver. 5.75 SP2 (Shimadzu, Kyoto, Japan).

MALDI-TOF was used to determine the occupation of HSA with sugar residues. Neo-glycoproteins were diluted in 50% acetonitrile/ 0.1% TFA (10 pmol/µL); the samples were complemented by an equal volume of the same solution saturated with sinapinic acid. The samples were then prepared by the dried droplet method by spotting the mixture onto the MALDI target and drying them at room temperature. The spectra were recorded on an

UltrafleXtreme MALDI-TOF/ TOF mass spectrometer (Bruker Daltonics, Germany) with a 1 kHz smartbeam II laser, accumulating 10,000 shots; samples were measured in a positive linear mode (mass range 20~200 kDa) and at an accelerating voltage of 25kV.

2.3. Nuclear Magnetic Resonance (NMR)

NMR analysis confirmed the structure of the produced *t*Boc-modified glycans **2-5** and **8**. NMR spectra of compounds **2**, **3**, and **4** were acquired on a Bruker Avance III 600 MHz and compounds **5** and **8** on a Bruker Avance III 700 MHz spectrometer (Bruker BioSpin, Rheinstetten, Germany) in D_2O at 30 °C. ^{1}H NMR, ^{13}C NMR, COSY, HSQC, HMBC, HSQC-TOCSY, and 1D-TOCSY experiments were performed using standard manufacturer's software (TopSpin 3.5, Bruker BioSpin, Rheinstetten, Germany). ^{1}H NMR spectra were referenced to the residual signal of D_2O (δ_H 4.732 ppm), ^{13}C NMR to the signal of acetone (δ_C 30.50 ppm). Spin systems of individual monosaccharide units were assigned using COSY and 1D-TOCSY experiments and transferred to carbons via HSQC and HSQC-TOCSY. The glycosidic linkage was proved by HMBC correlations between carbons C3 or C4 with the anomeric protons of the subsequent carbohydrate units.

3. Enzymes for Glycan Synthesis

3.1. Production and Purification of Enzymes

For the synthesis of glycans **2-5** and **7-11**, four enzymes were produced as N-terminal His-tagged constructs: β4-galactosyltransferase (β4GalT) from human placenta (pET16b; *Nco*I/*Xho*HI),[2] β3-*N*-acetylglucosaminyltransferase (β3GlcNAcT) from *Helicobacter pylori* (pCWori; *Nco*I/*Xho*I),[3] mutant human placental β4-galactosyltransferase with β4-*N*-acetylgalactosaminyltransferase activity (β4GalT-Y284L = β4GalNAcT; pET16b; *Nco*I/*Xho*HI),[4] and mutant β-galactosidase from *Bacillus circulans,* acting as β3-

galactosynthase (BgaC-E233G; pET-Duet-1; *Bam*HI/*Pst*I).[5] The β4-GalT and β4-GalNAcT protein constructs contained an N-terminal lipase pre-propeptide from *Staphylococcus hyicus* (202 aa; aa 39-240 of the original sequence) followed by the C-terminal part of GalT catalytic domain (323 aa; aa 75-397 of the original sequence). The GalT construct contained one silent mutation in the nucleotide sequence, namely I210V. Two more silent mutations besides the target mutation Y284L are present in the GalNAcT construct: E75Q and T114A. These silent mutations were located outside the catalytic domain and did not affect catalytic abilities.

The production and purification of all enzymes were performed as previously described. Specific *Escherichia coli* strains were utilized for enzyme production: BL21(DE3) for β4GalT, β4GalNAcT, and β3GlcNAcT, and BL21 Gold(DE3) for BgaC-E233G. Transformed cells were initially cultured in 60 mL of Luria-Bertani (LB) medium (10 g/L tryptone, 5 g/L yeast extract, 5 g/L NaCl) supplemented with appropriate antibiotics depending on the strain and plasmid, in 0.5 L flasks at 37 °C and 220 rpm overnight. This pre-culture was used to inoculate 600 mL of Terrific Broth (TB) medium (24 g/L yeast extract, 12 g/L tryptone, 4 mL/L glycerol, 17 mM KH_2PO_4, 72 mM K_2HPO_4, pH 7.5) containing respective antibiotics in 3 L flasks. Main cultures were grown at 37 °C and 150 rpm until the optical density at 600 nm (OD600) reached 0.6–0.8. Enzyme expression was induced with 0.5 mM IPTG, and the cultures were incubated for further 24 h at 25 °C and 100 rpm. Cells were harvested by centrifugation at 8,880 × g for 20 min at 4 °C and stored at −20 °C.

For purification, the harvested cells were sonicated (1 min pulse, 2 min pause, six cycles) in the respective equilibration buffer. The resulting cell-free extract was loaded onto an equilibrated affinity chromatography column. N-terminal His-tagged protein constructs (β4GalT, β4GalNAcT) were purified using a HisTrap™ column with an equilibration buffer of 100 mM HEPES, 500 mM NaCl, 20 mM imidazole pH 7.4, and eluted with an increased imidazole concentration (100 mM HEPES, 500 mM NaCl, 500 mM imidazole, pH 7-7.4). The

N-terminal His-tagged protein construct BgaC-E233G, was purified using a HisTrap™ column with an equilibration buffer of 20 mM phosphate, 500 mM NaCl, 20 mM imidazole pH 7.4, and eluted with an increased imidazole concentration (20 mM phosphate, 500 mM NaCl, 500 mM imidazole, pH 7-7.4). The maltose-binding-protein (MBP)-fusion construct β3GlcNAcT was purified using an MBPTrap™ column with an equilibration buffer of 20 mM Tris-HCl, 200 mM NaCl, 1 mM EDTA, 1 mM DTT pH 7.4, and eluted with 10 mM maltose in the equilibration buffer. In certain reactions as specified further, crude β4GalT without purification was employed to increase the enzyme yield. All enzymes were dialyzed overnight against PBS buffer, and their concentrations were determined using the Bradford assay[6] calibrated for bovine plasma γ-globulin (IgG, Bio-Rad, UK).

3.2. Standard Enzyme Activity Assay

The catalytic activity of recombinant glycosyltransferases was determined as follows: The respective UDP-sugar glycosyl donor (UDP-GlcNAc for β3GlcNAcT, UDP-Gal for β4GalT, UDP-GalNAc for β4GalNAcT; 6.5 mM) was mixed with the respective glycosyl acceptor (LN2-*t*Boc or Lac-*t*Boc for β3GlcNAcT, GlcNAc-LN2-*t*Boc or GlcNAc-Lac-*t*Boc for β4GalNAcT and β4GalT; 5 mM), lactate dehydrogenase (20 U/mL), pyruvate kinase (20 U/mL), NADH (0.25 mM), and phosphoenolpyruvate (1 mM) in 100 mM HEPES/25 mM KCl buffer pH 7 with $MgCl_2$ (5 mM) for β3GlcNAcT or $MnCl_2$ (4 mM) for β4GalT and β4GalNAcT. After pre-incubation (5 min) at 37 °C, the appropriately diluted enzyme was added (final reaction volume 100 μL). The linear decline in NADH concentration from the coupled reaction was monitored for 15 min at 340 nm.

The catalytic activity of the glycosynthase BgaC-E233G was determined based on the rescue of hydrolytic activity in the presence of a small external nucleophile.[7] The rescue of the hydrolytic activity was accomplished by adding the exogenous nucleophile (sodium formate) in a concentration of 0.5–7.5 M to the glycosidase enzyme activity assay, using 2 mM *p*NP-β-

Gal substrate in a sodium phosphate buffer (50 mM NaH_2PO_4/50 mM Na_2HPO_4, pH 6.5). The reaction mixture was incubated at 35 °C in a thermoshaker and the reaction was started by adding 10 µL of not diluted BgaC-E233G. The activity assay ran for 10 min at 850 rpm and was stopped by adding 1 mL of 0.1 M sodium carbonate. The liberated *p*-nitrophenol in the form of *p*-nitrophenolate formed under basic conditions was then detected spectrophotometrically at 420 nm.

4. Chemoenzymatic Synthesis of Functionalized Glycans

4.1. Synthesis of Functionalized Glycans

(*tert*-Butoxycarbonylamino)ethylthioureidyl 2-acetamido-2-deoxy-β-D-glucopyranosyl-(1→3)-β-D-galactopyranosyl-(1→4)-2-acetamido-2-deoxy-β-D-glucopyranoside (2; GlcNAc-Lac-*t*Boc): Trisaccharide **2** was produced from Lac-*t*Boc (**1**; for HRMS and HPLC analyses see Figures S2a-S3c)[8–11] in a reaction catalyzed by *H. pylori* β3GlcNAcT.[4] The acceptor Lac-*t*Boc **1** (140 mg, 5 mM) was mixed with the donor UDP-GlcNAc (197 mg, 6.5 mM) in 100 mM sodium phosphate/25 mM KCl/5 mM $MgCl_2$/1 mM DTT buffer, pH 7 (total reaction volume 50 mL) and the enzyme β3GlcNAcT (0.02 U/mL in the reaction) was added. After confirming complete conversion by TLC, product **2** was purified by SPE and GPC as stated in the main text, resulting in 160 mg of pure **2** (85% yield). NMR, HRMS and HPLC analyses are shown in Table S2, Figures S3a-S3e.

(*tert*-Butoxycarbonylamino)ethylthioureidyl β-D-galactopyranosyl-(1→3)-2-acetamido-2-deoxy-β-D-glucopyranosyl-(1→3)-β-D-galactopyranosyl-(1→4)-β-D-glucopyranoside (3; LN1-Lac-*t*Boc): Galactosyl donor α-Gal-F (4.5 mg, 10 mM), prepared as described previously,[9] and GlcNAc-Lac-*t*Boc acceptor (**2**; 19 mg, 10 mM) were dissolved in 50 mM sodium phosphate buffer pH 6.5, and β3-galactosynthase BgaC-E233G (18 mg, 271 µM, 2.0

mL) was added (total reaction volume 2.5 mL). The reaction was incubated at 30 °C and 850 rpm, and monitored by TLC. When the donor was nearly consumed (ca after 20-24 h), another portion of α-Gal-F was added. Then, after 48 h the reaction was stopped by enzyme denaturation and purified by SPE and gel chromatography as described in the General procedure in the main text, affording 16 mg of **3** (70% yield), and analyzed by NMR, HRMS and HPLC (Table S3, Figures S4a-S4e).

(*tert*-Butoxycarbonylamino)ethylthioureidyl β-D-galactopyranosyl-(1→4)-2-acetamido-2-deoxy-β-D-glucopyranosyl-(1→3)-β-D-galactopyranosyl-(1→4)-β-D-glucopyranoside (4; LN2-Lac-*t*Boc): UDP-Gal (74 mg, 6.5 mM) donor and acceptor trisaccharide **2** (79 mg, 5 mM) were combined with crude β4GalT (0.03 U/mL in the reaction) in 100 mM sodium phosphate/25 mM KCl/2 mM $MnCl_2$ buffer, pH 7 (total reaction volume 20 mL). The conversion as monitored by TLC was 100%. Purification by GPC (see the General procedure in the main text) yielded 90 mg of product **4** (94% yield), which was optionally additionally purified by HPLC, and analyzed by NMR, HRMS and HPLC (Table S4, Figures S5a-S5e).

(*tert*-Butoxycarbonylamino)ethylthioureidyl 2-acetamido-2-deoxy-β-D-galactopyranosyl-(1→4)-2-acetamido-2-deoxy-β-D-glucopyranosyl-(1→3)-β-D-galactopyranosyl-(1→4)-2-β-D-glucopyranoside (5; LDN-Lac-*t*Boc): UDP-GalNAc (54 mg, 6.5 mM) donor was combined with acceptor trisaccharide **2** (50 mg, 5 mM) and β4GalNAcT (0.03 U/mL of the reaction mixture) in 100 mM HEPES/25 mM KCl/2 mM $MnCl_2$ buffer, pH 6.8 (total reaction volume 13 mL). The reaction was re-fed with the same amount of enzyme and UDP-GalNAc as used in the initial reaction. The final conversion of tetrasaccharide **5** was ca. 40%. After purification by SPE and GPC (see the General procedure in the main text) the

final yield of tetrasaccharide **5** was 14 mg, 22%. Analyses by NMR, HRMS and HPLC are shown in Table S5, Figures S6a-S6e.

(*tert*-Butoxycarbonylamino)ethylthioureidyl 2-acetamido-2-deoxy-β-D-glucopyranosyl-(1→3)-β-D-galactopyranosyl-(1→4)-2-acetamido-2-deoxy-β-D-glucopyranoside (8; GlcNAc-LN2-*t*Boc): Trisaccharide **8** was produced from LacNAc-*t*Boc (**7**; for HRMS and HPLC analyses see Figures S7a-S7c),[12] which was prepared under the catalysis by β-galactosidase from *Bacillus circulans* Biolacta FN5 (Daiwa Kasei K.K., Japan).[12] β-galactosidase (0.06 U/mL of the reaction mixture) was incubated with *p*-nitrophenyl β-D-galactopyranoside (*p*NP-Gal; 30 mM) donor and GlcNAc-*t*Boc (**6**; 150 mM) acceptor in sodium acetate buffer pH 5 at 35 °C, under monitoring by TLC (isopropyl alcohol/H_2O/NH_4OH aq.; 7/2/1 *v*/*v*/*v*). After 4-5 hours, the reaction was terminated by heating (99 °C, 2 min), and LN2-*t*Boc **7** was purified by GPC as described in the General procedure in the main text, resulting in 160 mg of pure **7** (40% yield). In the next step, *H. pylori* β3GlcNAcT (0.03 U/mL in the reaction mixture) catalyzed the glycosylation of LN2-*t*Boc **7** (140 mg, 5 mM) acceptor using UDP-GlcNAc (197 mg, 6.5 mM) as a donor in 100 mM sodium phosphate/25 mM KCl/5 mM $MgCl_2$/1 mM DTT buffer pH 7 (total reaction volume 50 mL) overnight at 37 °C. To reach complete conversion, the reaction was re-fed with the same amount of β3GlcNAcT and UDP-GlcNAc as used in the initial reaction. The reaction was monitored by TLC and was stopped after 24-48 hours when practically complete conversion was achieved. Product **8** was purified only by SPE as described in the General procedure, affording GlcNAc-LN2-*t*Boc **8** (235 mg, 97% yield). For NMR, HRMS and HPLC analyses see Table S6, Figures S8a-S8e.

(*tert*-Butoxycarbonylamino)ethylthioureidyl β-D-galactopyranosyl-(1→3)-2-acetamido-2-deoxy-β-D-glucopyranosyl-(1→3)-β-D-galactopyranosyl-(1→4)-2-acetamido-2-deoxy-β-

D-glucopyranoside (9; LN1-LN2-*t*Boc): Galactosyl donor α-Gal-F (18.2 mg, 10 mM), prepared as described previously,[9] and acceptor GlcNAc-LN2-*t*Boc (**8**; 79 mg, 10 mM) were dissolved in 50 mM sodium phosphate buffer pH 6.5, and β3-galactosynthase BgaC-E233G (74 mg, 559 µM, 5270 µL) was added (total reaction volume 10 mL). The reaction was incubated at 30 °C and 850 rpm and monitored by TLC. When all donor was consumed (ca after 20-24 h), the reaction was stopped by enzyme denaturation (99 °C, 2 min), and purified by SPE and GPC as described in the General procedure, affording 80 mg of **9** (84% yield). The structure of **9** was confirmed by NMR and was in accord with the published data.[13] HRMS and HPLC analyses are shown in Figures S9a-S9c.

(*tert*-Butoxycarbonylamino)ethylthioureidyl β-D-galactopyranosyl-(1→4)-2-acetamido-2-deoxy-β-D-glucopyranosyl-(1→3)-β-D-galactopyranosyl-(1→4)-2-acetamido-2-deoxy-β-D-glucopyranoside (10; LN2-LN2-*t*Boc): UDP-Gal (74 mg, 6.5 mM) as a donor and trisaccharide **6** (79 mg, 5 mM) as an acceptor were combined with crude β4GalT (0.03 U/mL in the reaction mixture) in 100 mM sodium phosphate/25 mM KCl/2 mM $MnCl_2$ buffer, pH 7 (total reaction volume 20 mL). After complete conversion was detected by TLC, purification by GPC (see the General procedure) yielded 90 mg of product **10** (94% yield), which was optionally additionally purified by HPLC. The structure of **10** was confirmed by NMR and was in accord with the published data.[13] HRMS and HPLC characterizations are shown in Figures S10a-S10c.

(*tert*-Butoxycarbonylamino)ethylthioureidyl 2-acetamido-2-deoxy-β-D-galactopyranosyl-(1→4)-2-acetamido-2-deoxy-β-D-glucopyranosyl-(1→3)-β-D-galactopyranosyl-(1→4)-2-acetamido-2-deoxy-β-D-glucopyranoside (11; LDN-LN2-*t*Boc): UDP-GalNAc (55 mg, 6.5 mM) as a donor was combined with trisaccharide **6** (52 mg,

5 mM) as an acceptor and β4GalNAcT (0.03 U/mL of the reaction mixture) in 100 mM HEPES/25 mM KCl/2 mM $MnCl_2$ buffer pH 6.8 (total reaction volume 13 mL). The reaction was re-fed with the same amount of enzyme and UDP-GalNAc as used in the initial reaction. The final conversion of tetrasaccharide **11** was ca. 44%. After purification by SPE and GPC (see the General procedure) the isolated yield was 16 mg, 25%. The structure of **11** was confirmed by NMR and was in accord with the published data.[13] HRMS and HPLC characterizations are shown in the Figures S11a-S11c.

Table S1. Overview of enzymes used in this study and their reaction conditions.

Enzyme	Glycosyl formed	Glycosyl donor and reaction conditions	Reference
β4GalT	Galβ1,4	6.5 mM UDP-Gal in 100 mM HEPES/25 mM KCl/2 mM $MnCl_2$ pH 7	Sauerzapfe et al.[2]
β4GalTY284L (β4GalNAcT)	GalNAcβ1,4	6.5 mM UDP-GalNAc in 100 mM HEPES/25 mM KCl/2 mM $MnCl_2$ pH 6.8	Kupper et al.[4]
β3GlcNAcT	GlcNAcβ1,3	6.5 mM UDP-GlcNAc in 100 mM sodium phosphate/25 mM KCl/5 mM $MgCl_2$/1 mM DTT pH 7	Sauerzapfe et al.[3]
BgaC-E233G	Galα1,3	10 mM α-Gal-F in 50 mM sodium phosphate pH 6.5	Henze et al.[5]

Figure S1 shows an overview of the enzymatic reactions affording the desired oligosaccharides.

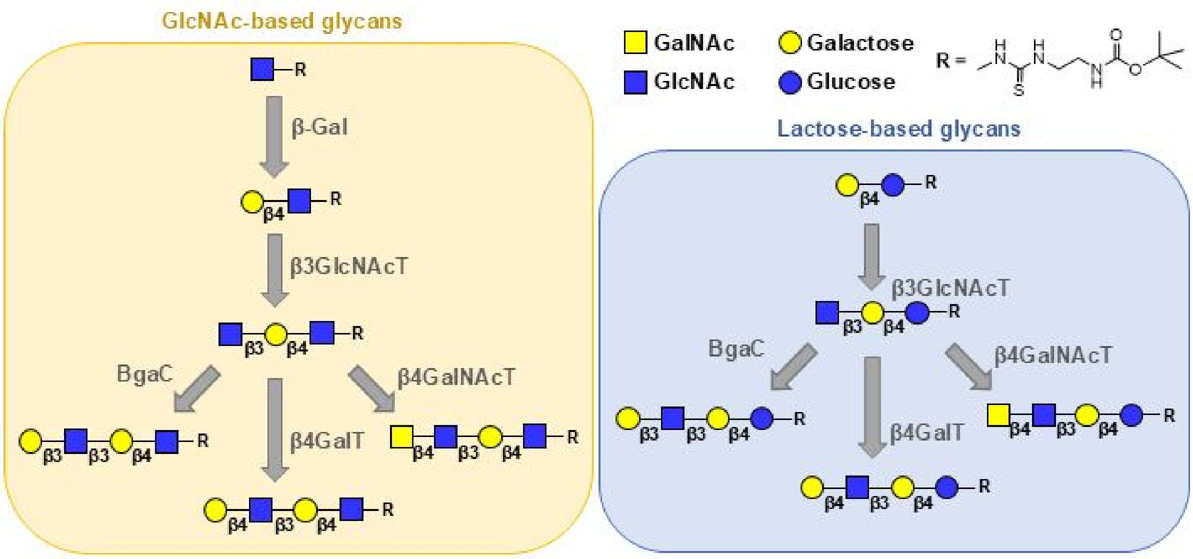

Figure S1. Enzymatic glycosylations affording target oligosaccharides. The yellow box represents glycans produced from GlcNAc-*t*Boc, the blue box shows glycans produced from Lac-*t*Boc.

5. Structural Analysis of Prepared Glycans and Neo-Glycoproteins

5.1. Glycans

NMR data are shown for unpublished compounds only. The data for the already published compounds were agreed with the literature.

Lac-*t*Boc (1)

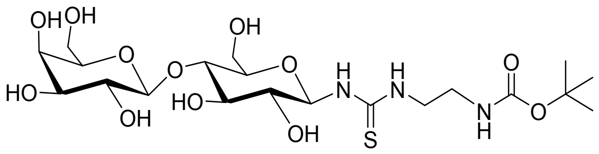

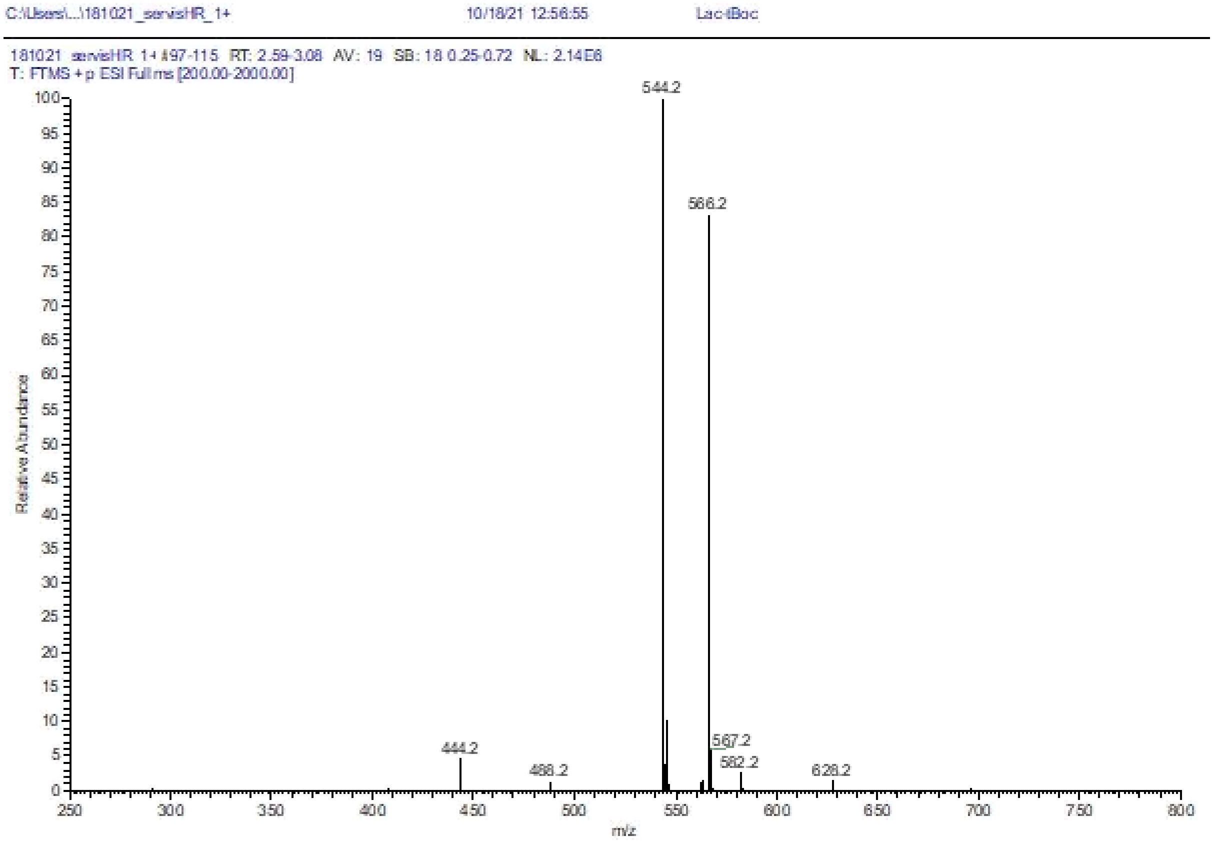

Figure S2a. ESI-MS(+) spectrum of compound **1**. $[M + H]^+$, *m/z* 544.2; $[M + Na]^+$, *m/z* 566.2; $[M + K]^+$, *m/z* 582.2.

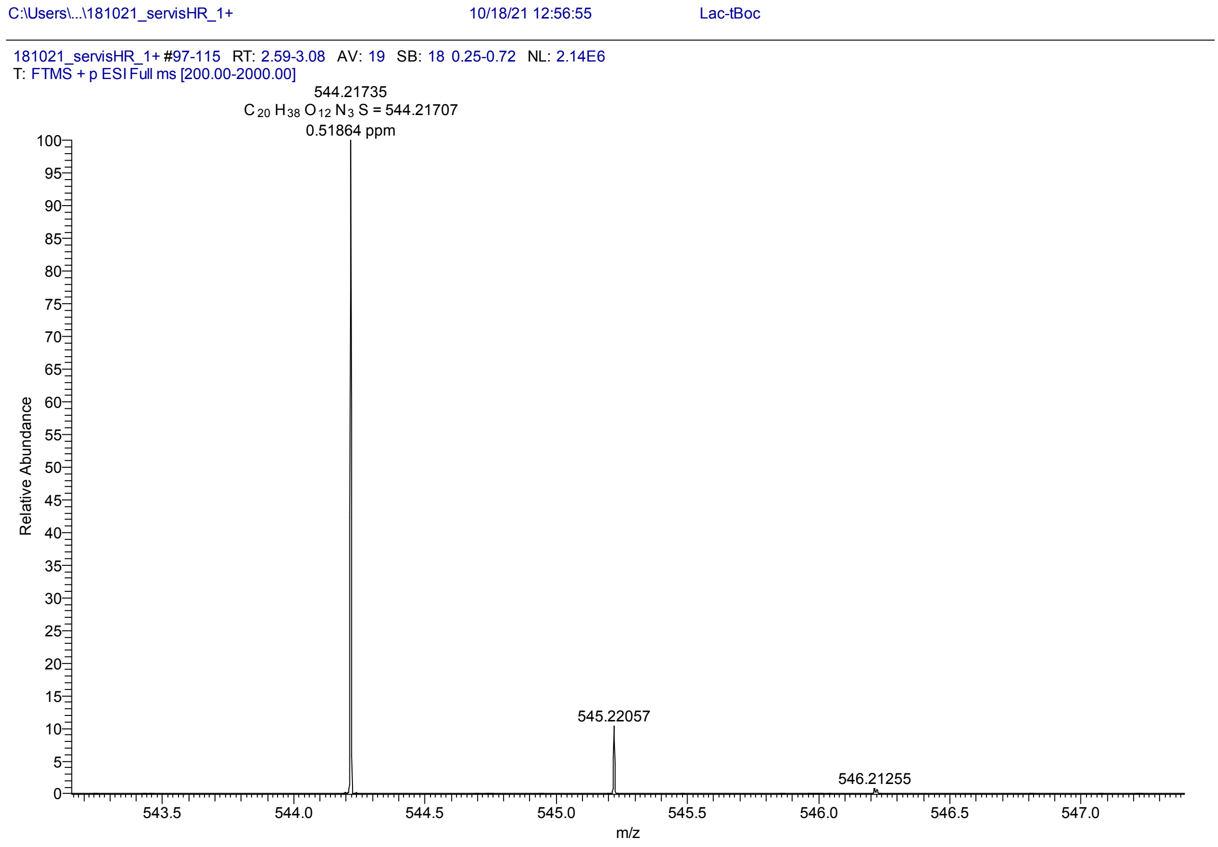

Figure S2b. High-resolution ESI-MS(+) of compound **1**. Calculated for $[M + H]^+$ ($C_{20}H_{38}N_3O_{12}S^+$) 544.21707, measured 544.21735 (0.52 ppm).

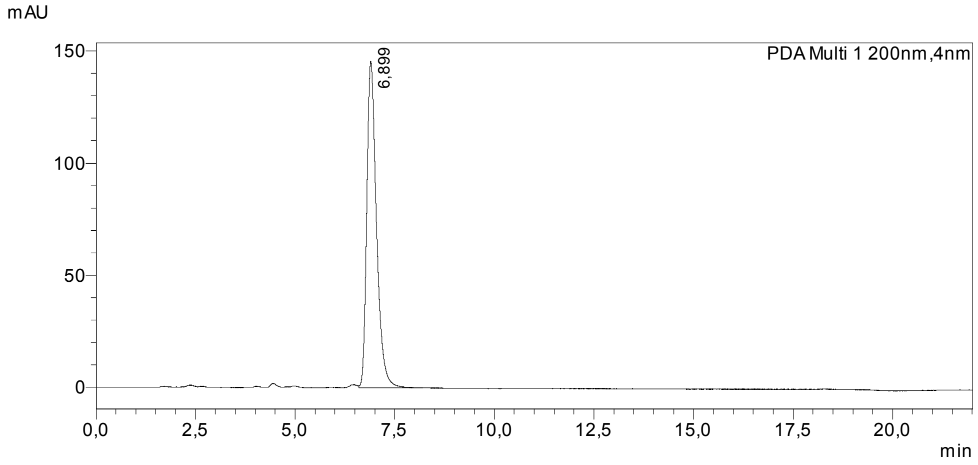

Figure S2c. HPLC chromatogram of compound **1** (retention time 6.899 min, purity 99%). Measured on HILIC column.

GlcNAc-Lac-*t*Boc (2)

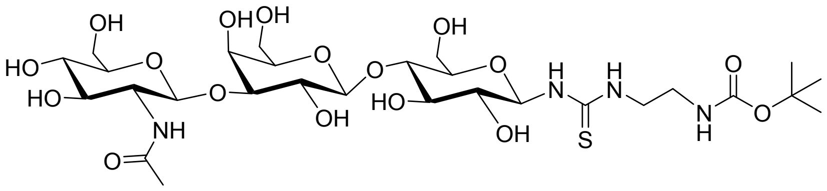

Table S2. ^{1}H and ^{13}C data of compound **2** (600.23 MHz for ^{1}H, 150.94 MHz for ^{13}C, D_2O, 30 °C).

	Atom	δ_C	m.	δ_H	n_H	m.	*J* [Hz]	Diagnostic HMBC
**Boc**	**CO**	158.52	S	-	0	-		2'
	**C**	81.39	S	-	0	-		$(CH_3)_3$
	**$(CH_3)_3$**	27.92	Q	1.473	9	s		$(CH_3)_3$
**spacer**	**1'**	44.55	T	3.732	2	br s		
	**2'**	39.50	T	3.336	2	br t	5.8	
	**CS**	n.d.	S	-	0	-		
**Glc^{C}**	**1**	83.26^{x}	D	5.63, 5.37^{x}	1	br s		
	**2**	71.92	D	3.54^{H}	1	m		
	**3**	75.31	D	3.75^{H}	1	m		
	**4**	78.07	D	3.72^{H}	1	m		1^{B}
	**5**	76.21	D	3.72^{H}	1	m		
	**6**	60.18	T	3.986	1	br d		
				3.86^{H}	1	m		
**Gal^{B}**	**1**	103.11	D	4.496	1	d	7.8	
	**2**	70.26	D	3.646	1	dd	9.9, 7.8	
	**3**	82.12	D	3.765	1	dd	9.9, 3.3	1^{A}
	**4**	68.60	D	4.187	1	dd	3.3, 0.7	
	**5**	75.13	D	3.76^{H}	1	m		
	**6**	61.19	T	3.81^{H}	2	m		
**GlcNAc^{A}**	**1**	103.01	D	4.736	1	d	8.5	3^{B}
	**2**	55.92	D	3.792	1	dd	10.4, 8.5	Ac
	**3**	73.82	D	3.610	1	dd	10.4, 8.5	
	**4**	69.97	D	3.512	1	dd	9.8, 8.5	
	**5**	75.91	D	3.481	1	ddd	9.8, 5.2, 2.2	
	**6**	60.77	T	3.938	1	dd	12.4, 2.2	
				3.800	1	dd	12.4, 5.2	
	**2-CO**	175.18	S	-	0	-		2, Ac
	**Ac**	22.41	Q	2.080	3	s		

^{x} … tentative assignment; ^{H} … HSQC readout; n.d. … not detected; The sample contains *ca.* 23% of parent compound **1**.

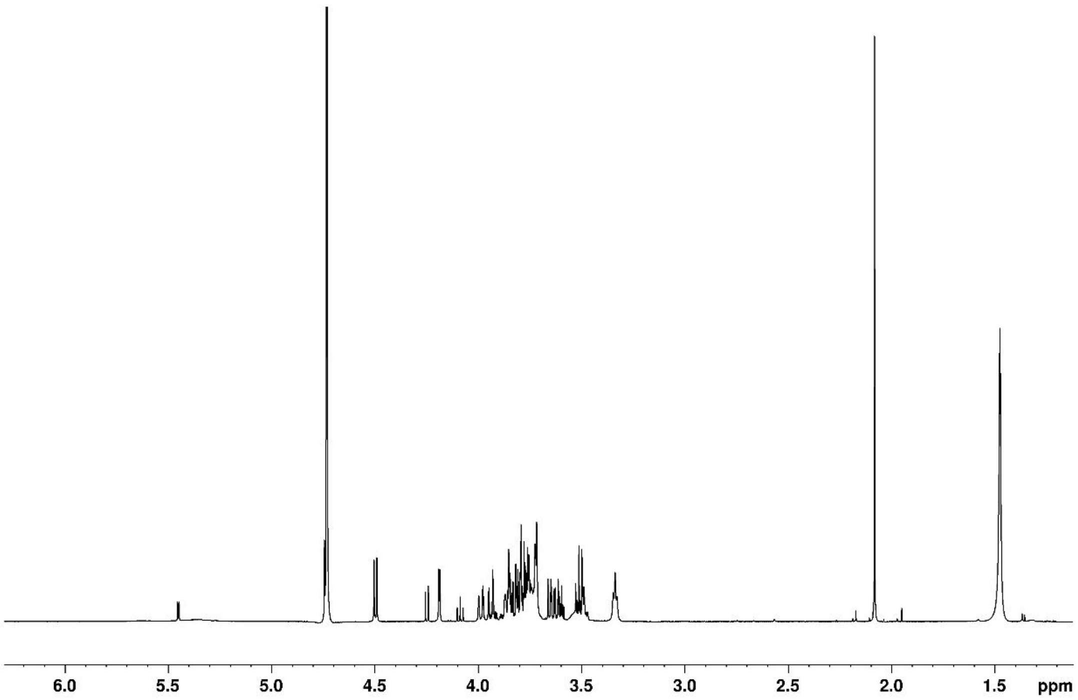

Figure S3a. ^{1}H NMR spectrum of compound **2** (600.23 MHz, CD_3OD, 30 °C).

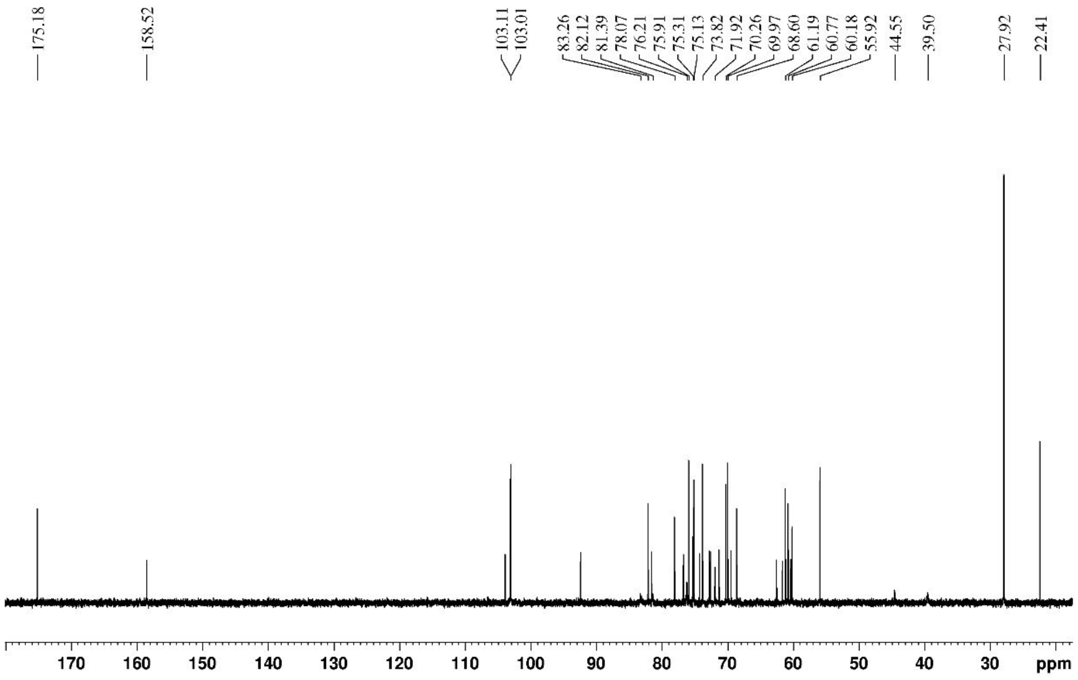

Figure S3b. ^{13}C NMR spectrum of compound **2** (150.94 MHz, CD_3OD, 30 °C). Signals of the impurity (parent compound **1**) are not labeled.

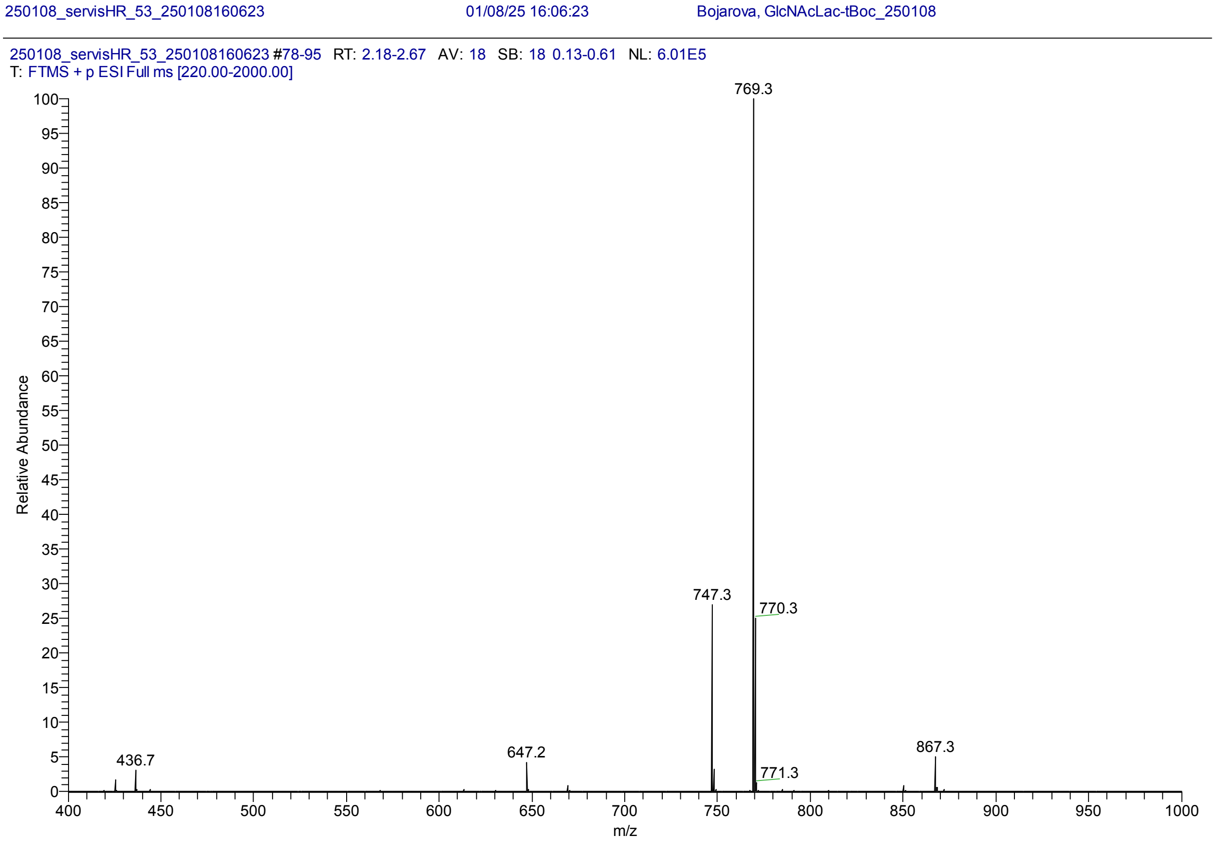

Figure S3c. ESI-MS(+) spectrum of compound **2**. $[M + H]^+$, *m/z* 747.3; $[M + Na]^+$, *m/z* 769.3.

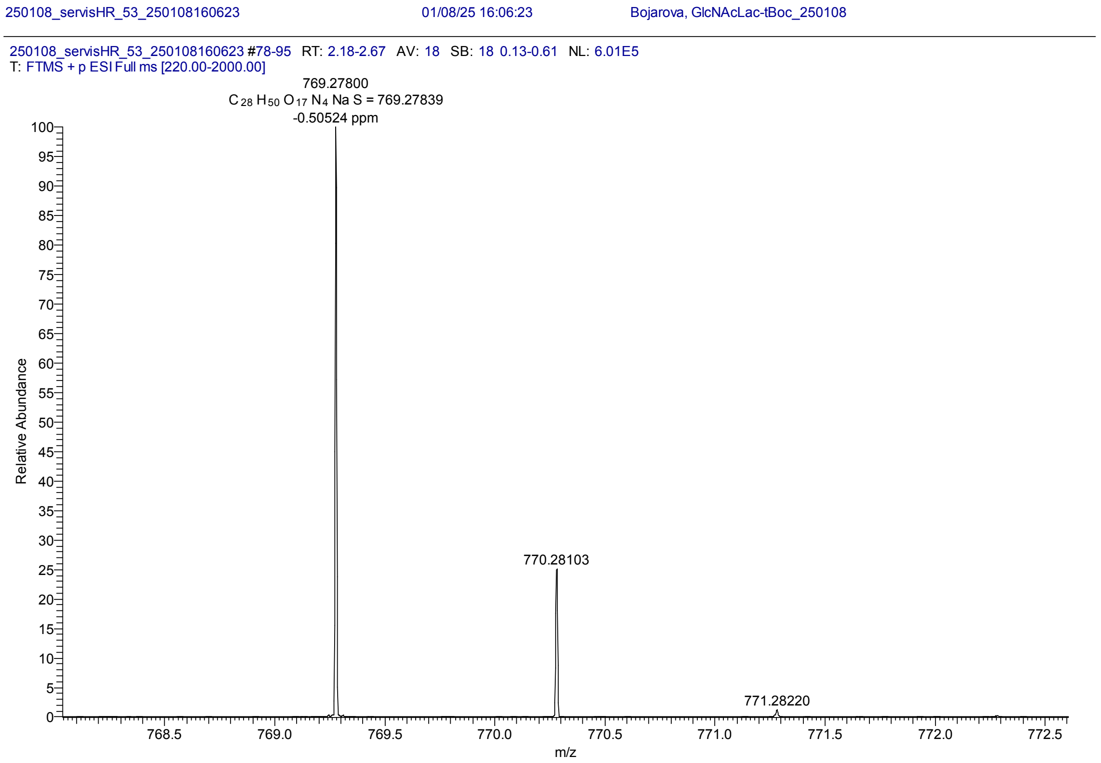

Figure S3d. High-resolution ESI-MS(+) of compound **2**. Calculated for $[M + Na]^+$ ($C_{28}H_{50}N_4O_{17}SNa^+$) 769.27839, measured 769.27800 (−0.51 ppm).

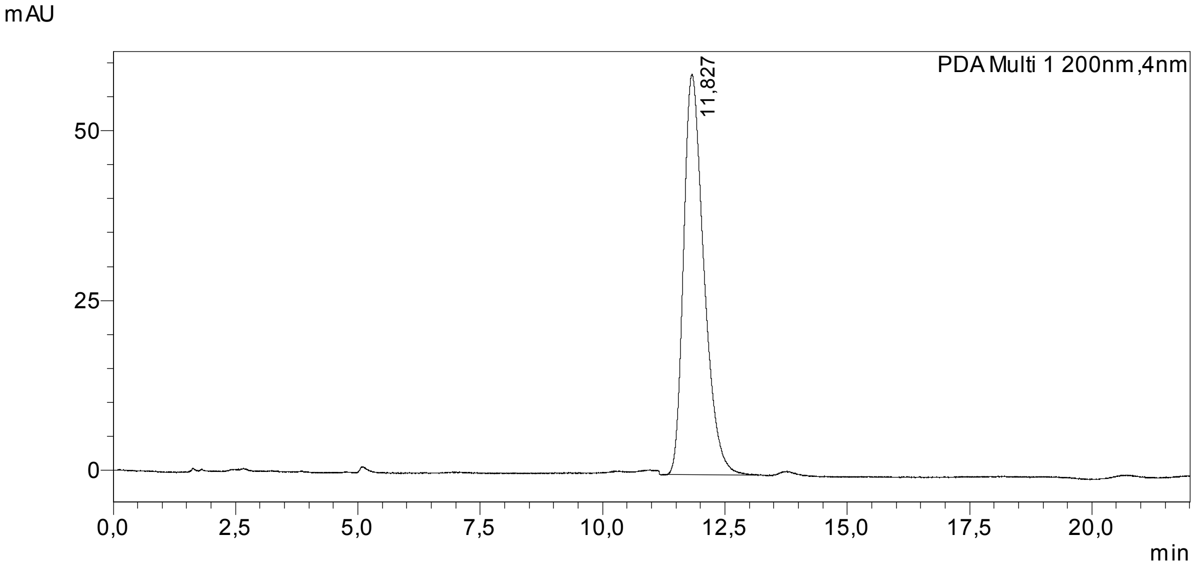

Figure S3e. HPLC chromatogram of compound **2** (retention time 11.827 min, purity 98%). Measured on a HILIC column.

LN1-Lac-*t*Boc (3)

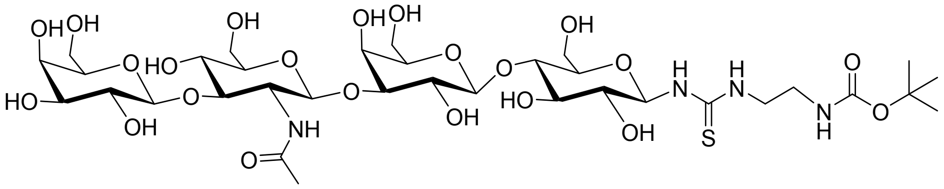

Table S3. ^{1}H and ^{13}C data of compound **3** (600.23 MHz for ^{1}H, 150.94 MHz for ^{13}C, D_2O, 30 °C).

	Atom	δ_C	m.	δ_H	n_H	m.	*J* [Hz]	Diagnostic HMBC
**Boc**	**CO**	158.53	S	-	0	-	-	2’
	**C**	81.38	S	-	0	-	-	$(CH_3)_3$
	**$(CH_3)_3$**	27.92	Q	1.450	9	s	-	
**spacer**	**1’**	44.56	T	3.71^{H}	2	m	-	
	**2’**	39.52	T	3.313	2	br t	5.6	
	**CS**	183.38^{x}	S	-	0	-	-	
**Glc^{D}**	**1**	83.27^{x}	D	5.61, 5.34^{x}	1	br s	-	
	**2**	71.91	D	3.51^{H}	1	m	-	
	**3**	75.31	D	3.73^{H}	1	m	-	
	**4**	78.05	D	3.70^{H}	1	m	-	1^{C}
	**5**	76.21	D	3.70^{H}	1	m	-	
	**6**	60.16	T	3.963	1	br d	12.0	
				3.83^{H}	1	m	-	
**Gal^{C}**	**1**	103.11	D	4.474	1	d	7.9	
	**2**	70.26	D	3.625	1	dd	9.9, 7.9	
	**3**	82.15	D	3.754	1	dd	9.9, 3.3	1^{B}
	**4**	68.58	D	4.169	1	d	3.3	
	**5**	75.14	D	3.74^{H}	1	m	-	
	**6**	61.20	T	3.78^{H}	2	m	-	
**GlcNAc^{B}**	**1**	102.75	D	4.758	1	d	8.4	3^{C}
	**2**	54.94	D	3.913	1	dd	10.4, 8.4	
	**3**	82.35	D	3.837	1	dd	10.4, 8.6	1^{A}
	**4**	68.72	D	3.588	1	dd	10.0, 8.6	
	**5**	75.44	D	3.498	1	ddd	10.0, 5.1, 2.3	
	**6**	60.77	T	3.92^{H}	1	m	-	
				3.797	1	dd	12.4, 5.1	
	**2-CO**	175.19	S	-	0	-	-	2^{B}, Ac^{B}
	**Ac**	22.47	Q	2.057	3	s	-	
**Gal^{A}**	**1**	103.71	D	4.457	1	d	7.8	3^{B}
	**2**	70.92	D	3.545	1	dd	9.9, 7.8	
	**3**	72.72	D	3.658	1	dd	9.9, 3.4	
	**4**	68.77	D	3.932	1	d	3.4	
	**5**	75.51	D	3.72^{H}	1	m	-	
	**6**	61.25	T	3.78^{H}	2	m	-	

^{x} … tentative assignment; ^{H} … HSQC readout

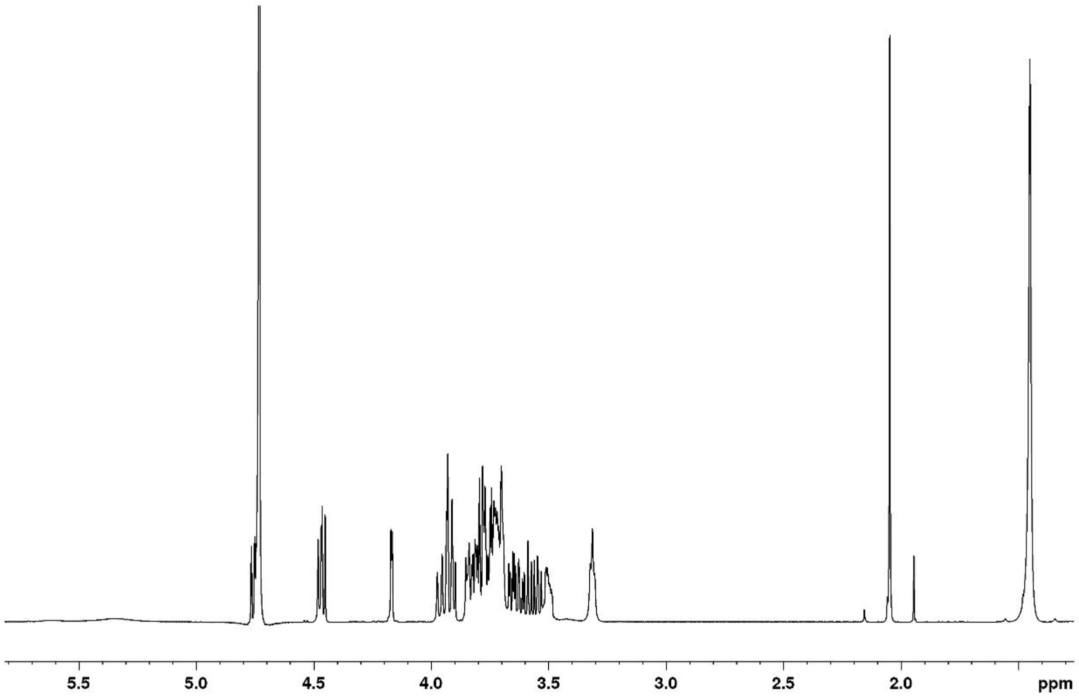

Figure S4a. ^{1}H NMR spectrum of compound **3** (600.23 MHz, D_2O, 30 °C).

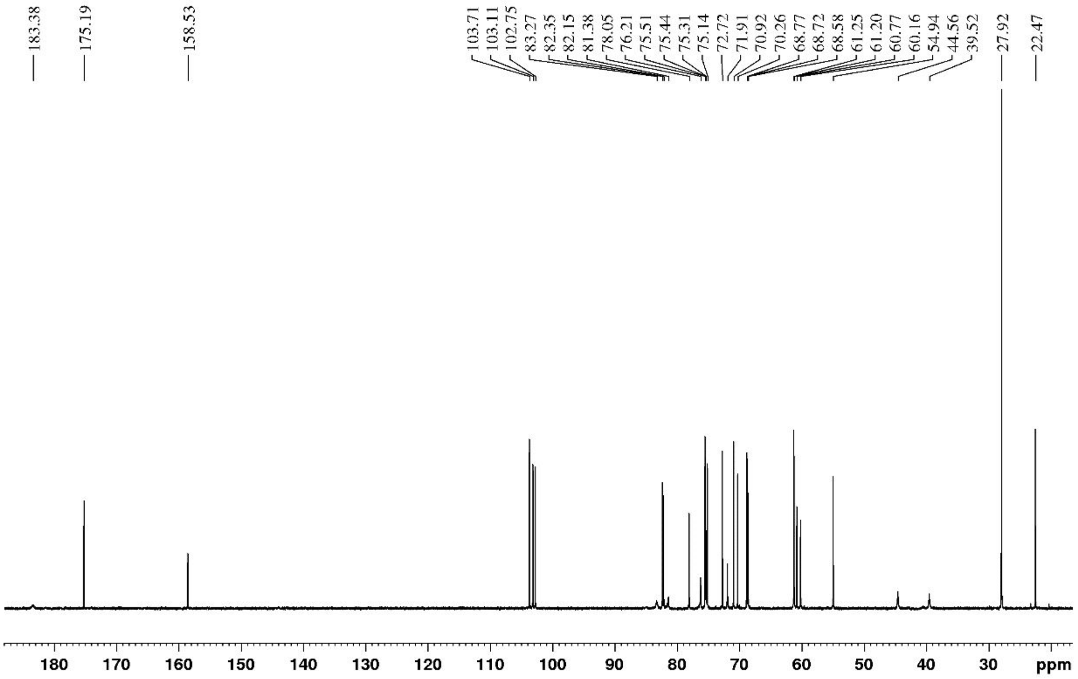

Figure S4b. ^{13}C NMR spectrum of compound **3** (150.94 MHz, D_2O, 30 °C).

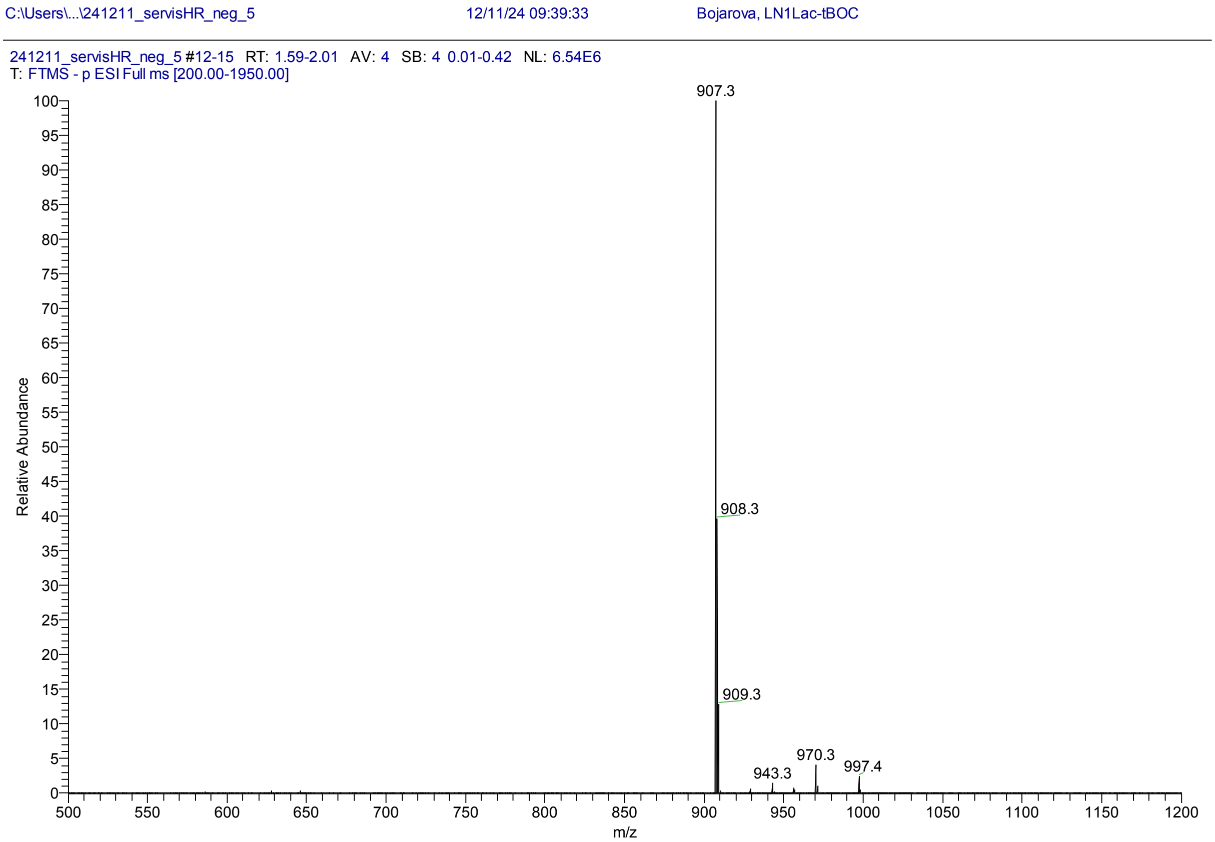

Figure S4c. ESI-MS(−) spectrum of compound **3**. [M − H]⁻, *m/z* 907.3.

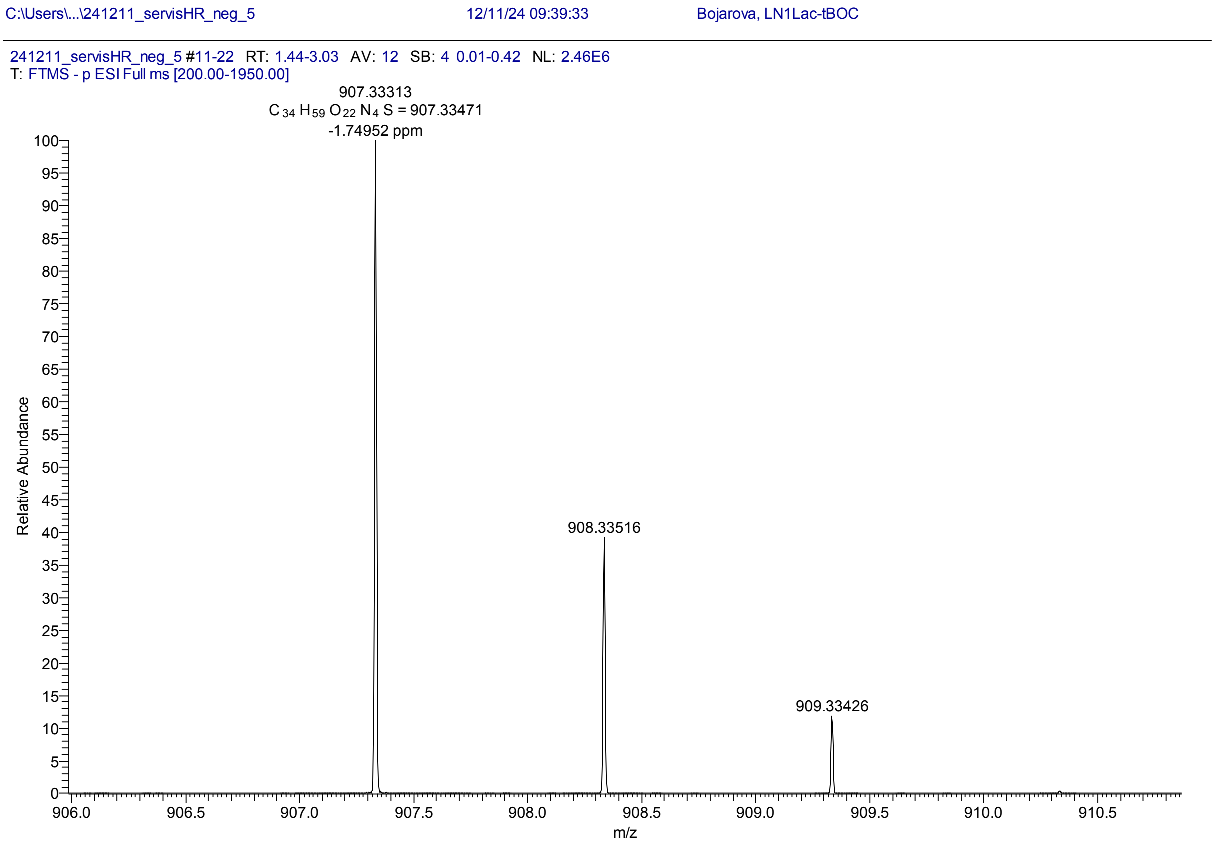

Figure S4d. High-resolution ESI-MS(+) of compound **3**. Calculated for $[M - H]^-$ ($C_{34}H_{59}N_4O_{22}S^-$) 907.33471, measured 907.33313 (−1.75 ppm).

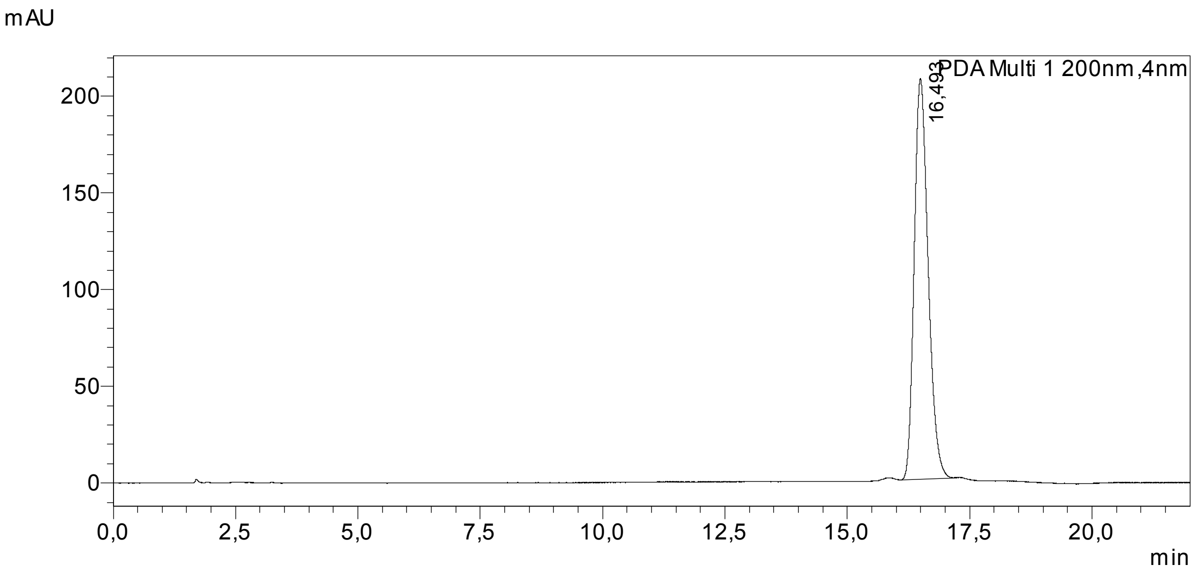

Figure S4e. HPLC chromatogram of compound **3** (retention time 16.493 min, purity 98%). Measured on a HILIC column.

LN2-Lac-*t*Boc (4)

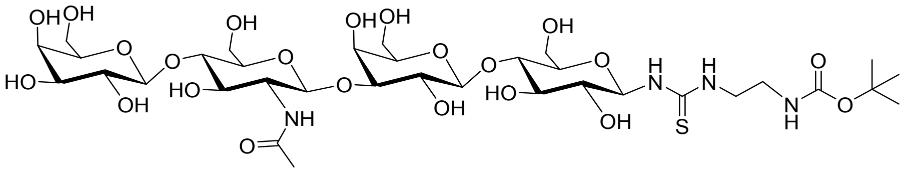

Table S4. ^{1}H and ^{13}C data of compound **4** (600.23 MHz for ^{1}H, 150.94 MHz for ^{13}C, D_2O, 30 °C).

	Atom	δ_C	m.	δ_H	n_H	m.	*J* [Hz]	Diagnostic HMBC
**Boc**	**CO**	158.53	S	-	0	-	-	2'
	**C**	81.37	S	-	0	-	-	$(CH_3)_3$
	**$(CH_3)_3$**	27.92	Q	1.453	9	s	-	
**spacer**	**1'**	44.56	T	3.712	2	br m	-	
	**2'**	39.51	T	3.316	2	br t	5.7	
	**CS**	183.36^{x}	S	-	0	-	-	
**Glc^{D}**	**1**	83.28^{x}	D	5.61, 5.34^{x}	1	br s	-	
	**2**	71.92	D	3.513	1	br dd	-	
	**3**	75.31	D	3.72^{H}	1	m	-	
	**4**	78.04	D	3.70^{H}	1	m	-	1^{C}
	**5**	76.21	D	3.70^{H}	1	m	-	
	**6**	60.16	T	3.966	1	br d	-	
				3.84^{H}	1	m	-	
**Gal^{C}**	**1**	103.11	D	4.476	1	d	7.9	
	**2**	70.22	D	3.625	1	dd	9.9, 7.9	
	**3**	82.21	D	3.745	1	dd	9.9, 3.3	1^{B}
	**4**	68.60	D	4.172	1	d	3.3	
	**5**	75.12	D	3.73^{H}	1	m	-	
	**6**	61.20	T	3.78^{H}	2	m	-	
**$GlcNAc^{B}$**	**1**	102.93	D	4.734	1	d	8.4	3^{C}
	**2**	55.45	D	3.823	1	dd	10.5, 8.4	
	**3**	72.42	D	3.76^{H}	1	m	-	
	**4**	78.51	D	3.76^{H}	1	m	-	1^{A}
	**5**	74.80	D	3.605	1	ddd	9.7, 4.8, 2.2	
	**6**	60.16	T	3.976	1	dd	12.3, 2.2	
				3.867	1	dd	12.3, 4.8	
	**2-CO**	175.13	S	-	0	-	-	2^{B}, Ac^{B}
	**Ac**	22.42	Q	2.057	3	s	-	
**Gal^{A}**	**1**	103.11	D	4.500	1	d	7.8	
	**2**	70.21	D	3.563	1	dd	10.0, 7.8	
	**3**	72.77	D	3.689	1	dd	10.0, 3.4	
	**4**	68.79	D	3.949	1	d	3.4	
	**5**	75.58	D	3.75^{H}	1	m	-	
	**6**	61.24	T	3.78^{H}	2	m	-	

^{x} … tentative assignment; ^{H} … HSQC readout

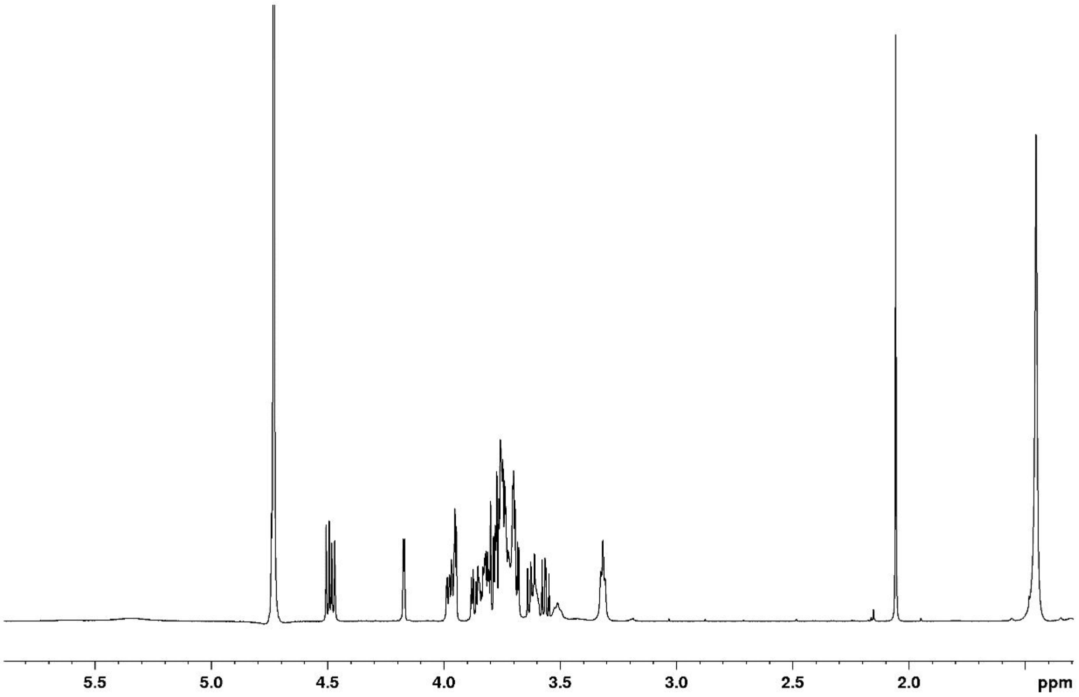

Figure S5a. ^{1}H NMR spectrum of compound **4** (600.23 MHz, D_2O, 30 °C).

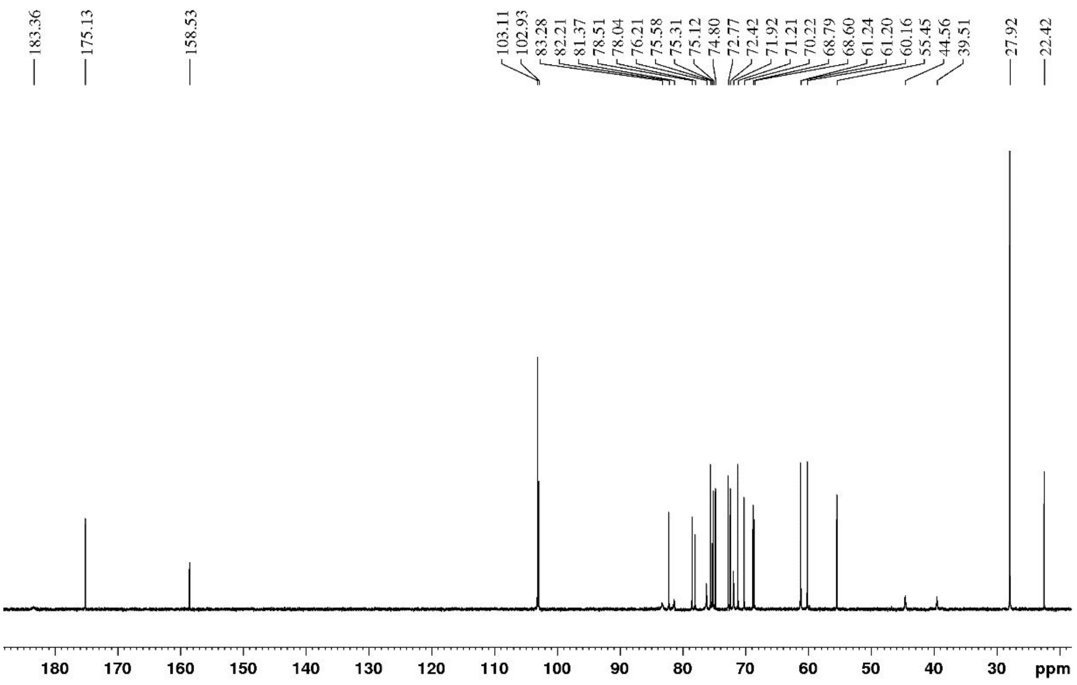

Figure S5b. ^{13}C NMR spectrum of compound **4** (150.94 MHz, D_2O, 30 °C).

C:\Users\...\241211_servisHR_neg_7 12/11/24 09:49:30 Bojarova, LN2Lac-tBOC

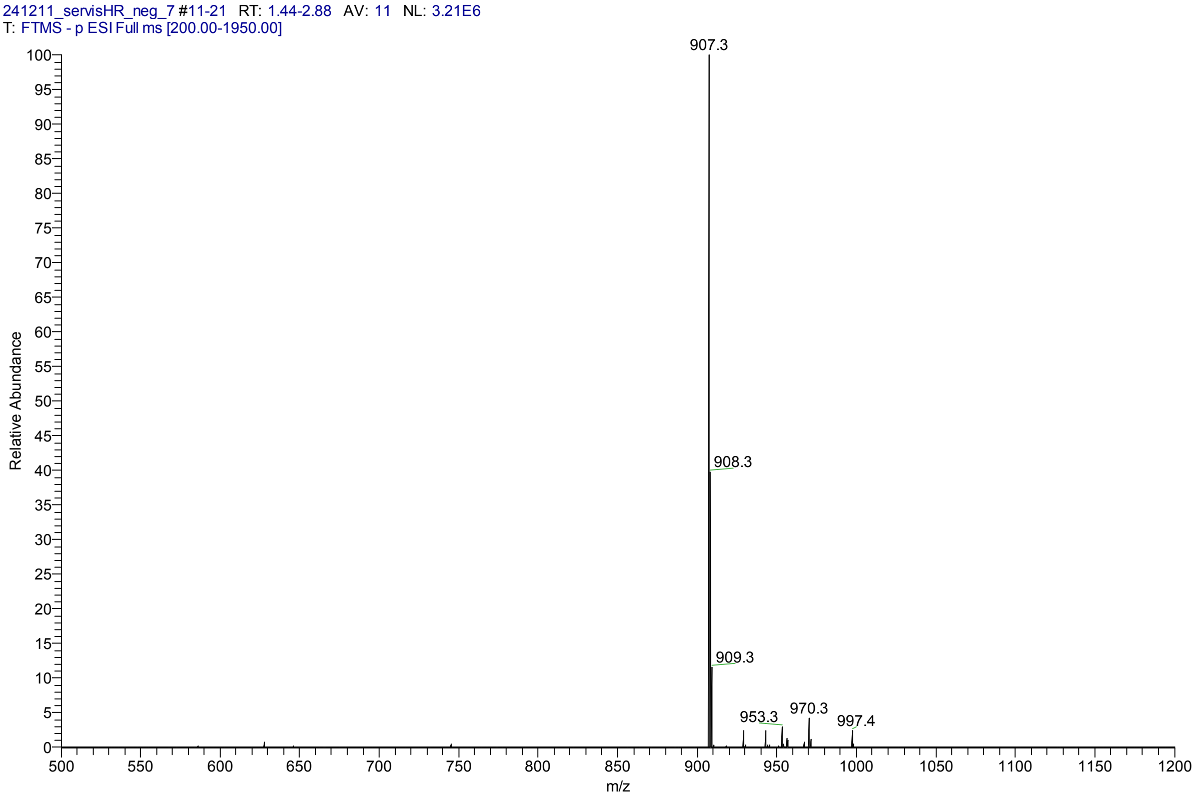

Figure S5c. ESI-MS(−) spectrum of compound **4**. [M − H]⁻, *m/z* 907.3.

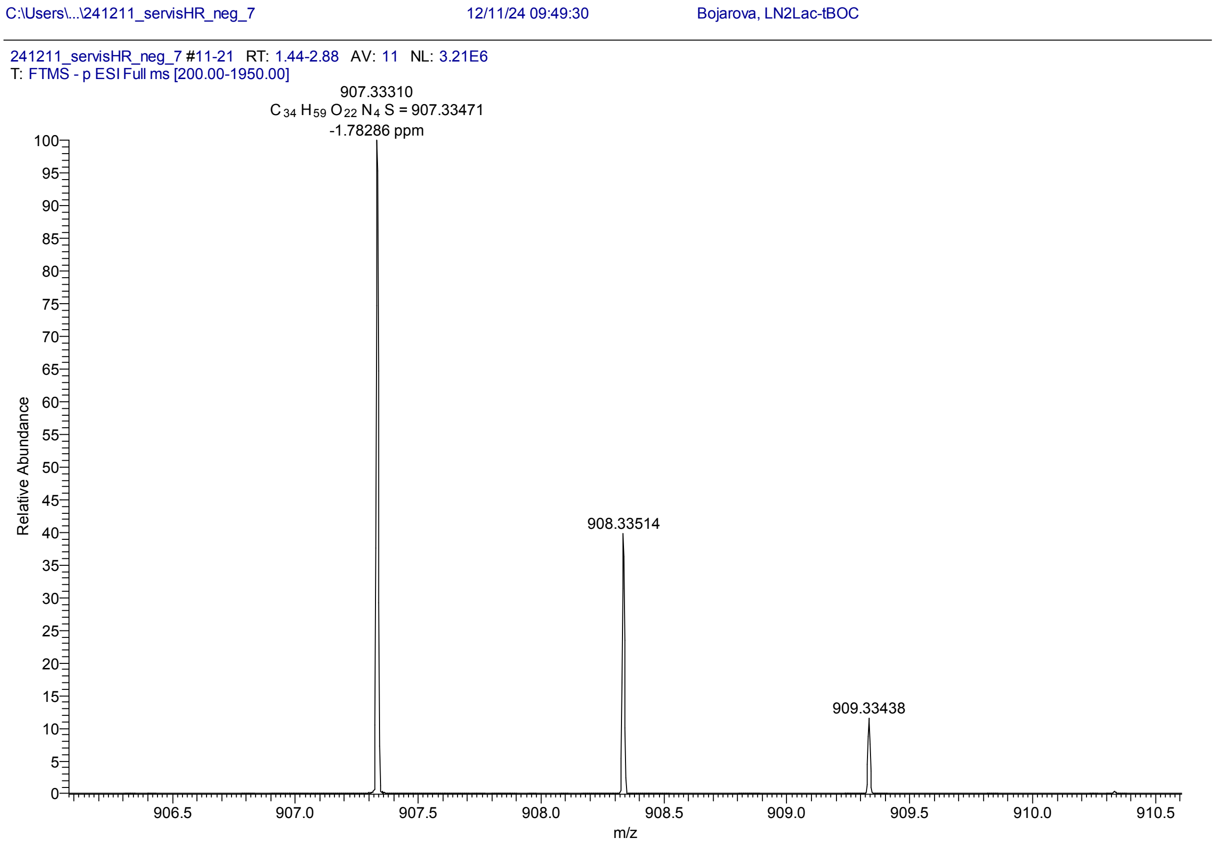

Figure S5d. High-resolution ESI-MS(+) of compound **4**. Calculated for $[M - H]^-$ ($C_{34}H_{59}N_4O_{22}S^-$) 907.33471, measured 907.33310 (−1.78 ppm).

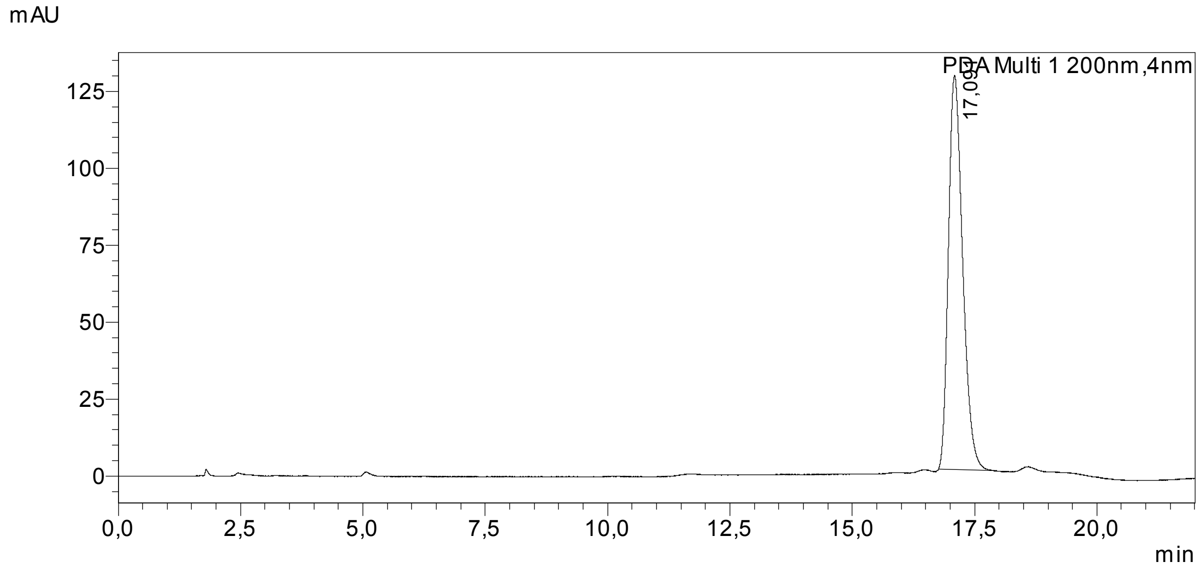

Figure S5d. HPLC chromatogram of compound **4** (retention time 17.091 min, purity 98%). Measured on a HILIC column.

LDN-Lac-*t*Boc (5)

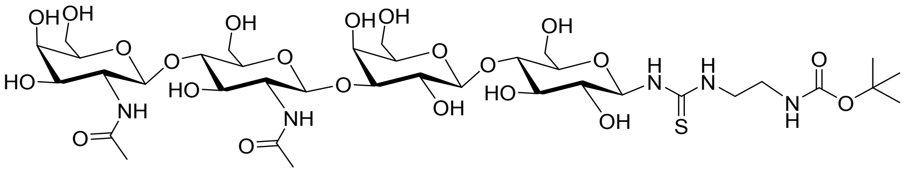

Table S5. ^{1}H and ^{13}C data of compound **5** (700.13 MHz for ^{1}H, 176.05 MHz for ^{13}C, D_2O, 30 °C).

	Atom	δ_C	m.	δ_H	n_H	m.	*J* [Hz]	Diagnostic HMBC
**Boc**	**CO**	158.50	S	-	0	-	-	2’
	**C**	81.33	S	-	0	-	-	$(CH_3)_3$
	**$(CH_3)_3$**	27.88	Q	1.439	9	s	-	
**spacer**	**1’**	44.53	T	3.70^H	2	m	-	
	**2’**	39.49	T	3.303	2	br t	5.5	
	**CS**	n.d.	S	-	0	-	-	
**Glc^D**	**1**	83.20^x	D	5.61, 5.34^x	1	br s	-	
	**2**	71.88	D	3.496	1	m	-	
	**3**	75.27	D	3.71^H	1	m	-	
	**4**	77.99	D	3.69^H	1	m	-	1^C
	**5**	76.17	D	3.69^H	1	m	-	
	**6**	60.11	T	3.950	1	m	-	
				3.82^H	1	m	-	
**Gal^C**	**1**	103.07	D	4.460	1	d	7.9	
	**2**	70.18	D	3.603	1	dd	9.9, 7.9	
	**3**	82.20	D	3.722	1	dd	9.9, 3.3	1^B
	**4**	68.54	D	4.145	1	dd	3.3, 0.8	
	**5**	75.07	D	3.72^H	1	m	-	
	**6**	61.16	T	3.78^H	2	m	-	
**GlcNAc^B**	**1**	102.88	D	4.697	1	d	8.3	3^C
	**2**	55.16	D	3.798	1	dd	10.5, 8.3	
	**3**	72.48	D	3.743	1	dd	10.5, 8.4	
	**4**	79.18	D	3.653	1	dd	9.8, 8.4	1^A
	**5**	74.53	D	3.520	1	ddd	9.8, 5.0, 2.2	
	**6**	60.19	T	3.845	1	dd	12.2, 2.2	
				3.661	1	dd	12.2, 5.0	
	**2-CO**	175.11	S	-	0	-	-	2^B, Ac^B
	**Ac**	22.37	Q	2.034	3	s	-	
**GalNAc^A**	**1**	101.93	D	4.534	1	d	8.4	4^B
	**2**	52.75	D	3.939	1	dd	10.8, 8.4	
	**3**	70.87	D	3.759	1	dd	10.8, 3.3	
	**4**	67.80	D	3.951	1	m	-	
	**5**	75.51	D	3.74^H	1	m	-	
	**6**	61.14	T	3.78^H	2	m	-	
	**2-CO**	174.96	S	-	0	-	-	2^A, Ac^A
	**Ac**	22.38	Q	2.073	3	s	-	

^{x} … tentative assignment; ^{H} … HSQC readout; n.d. … not detected

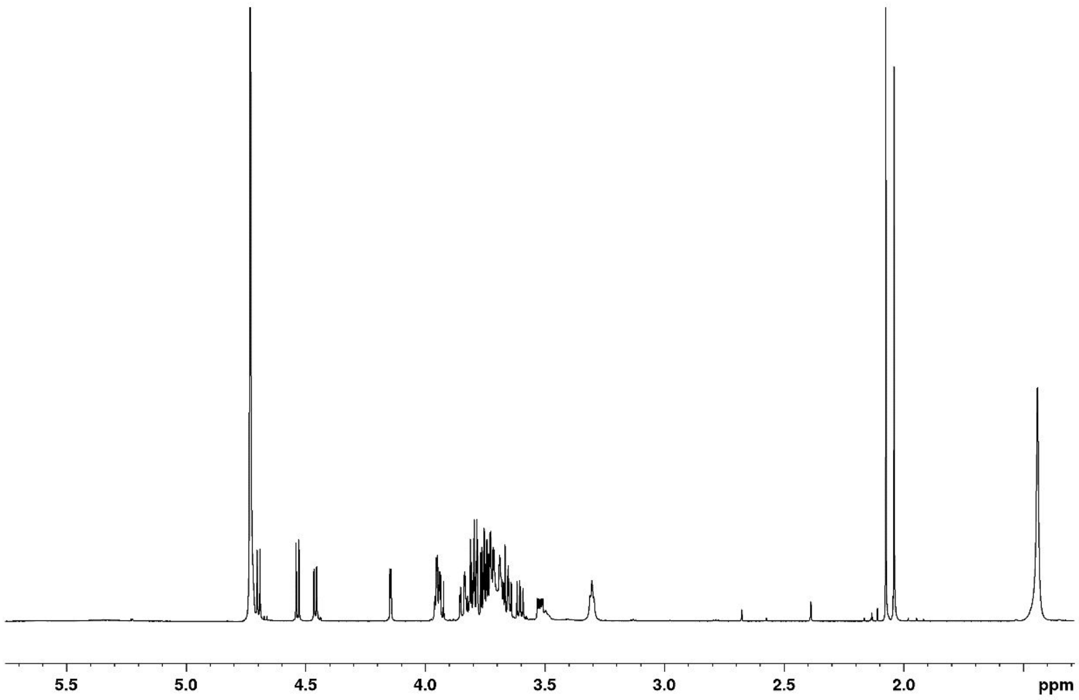

Figure S6a. ^{1}H NMR spectrum of compound **5** (700.13 MHz, D_2O, 30 °C).

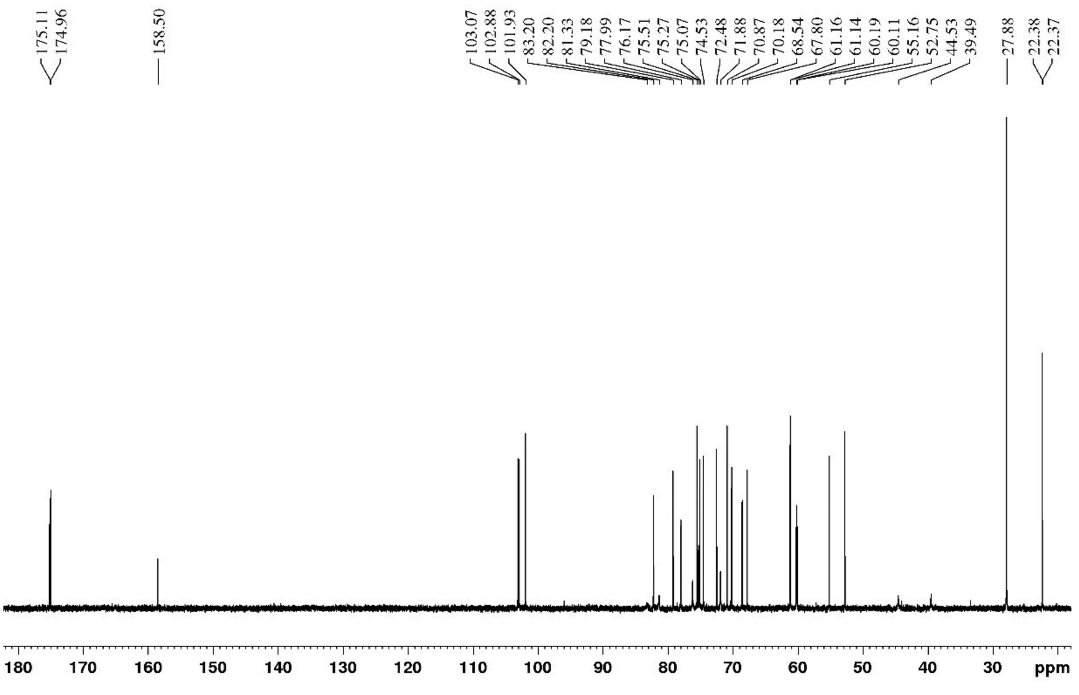

Figure S6b. ^{13}C NMR spectrum of compound **5** (176.05 MHz, D_2O, 30 °C).

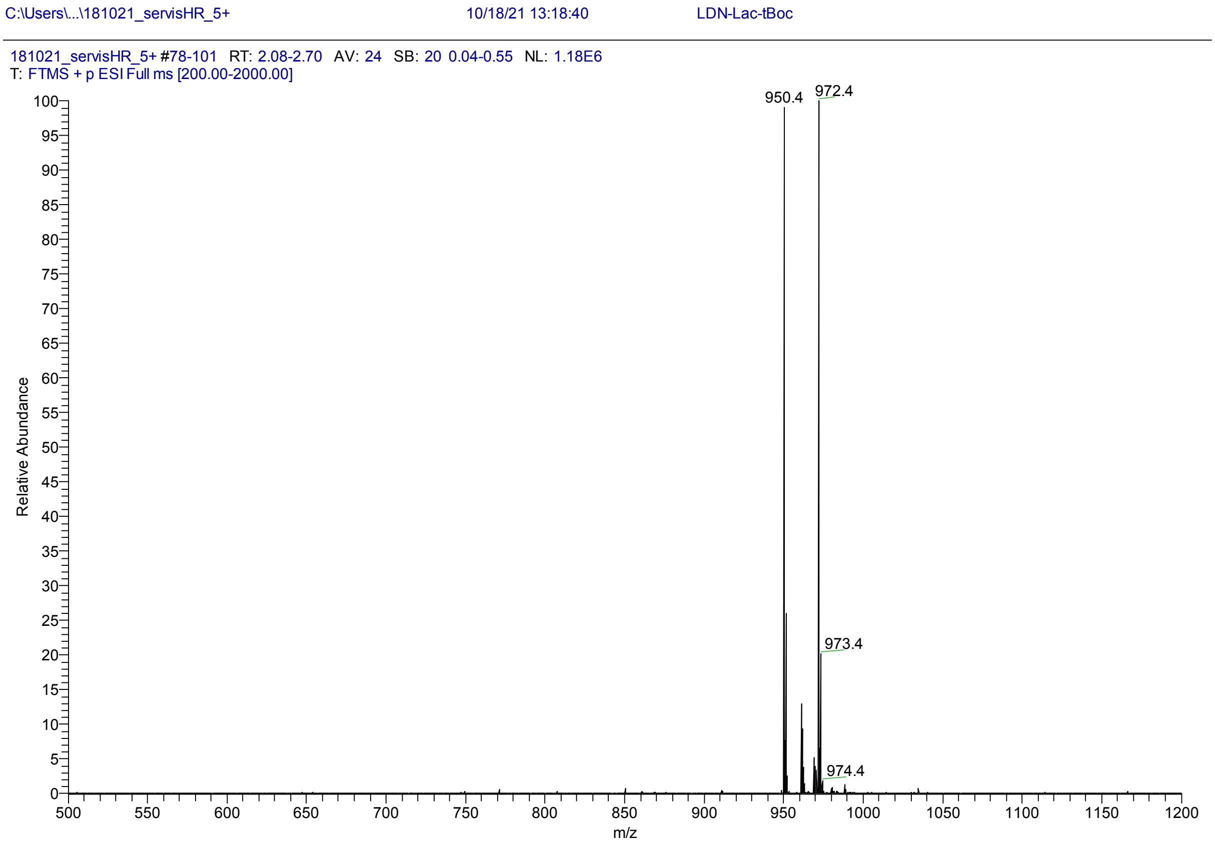

Figure S6c. ESI-MS(+) spectrum of compound **5**. [M + H]$^+$, *m/z* 950.4; [M + Na]$^+$, *m/z* 972.4.

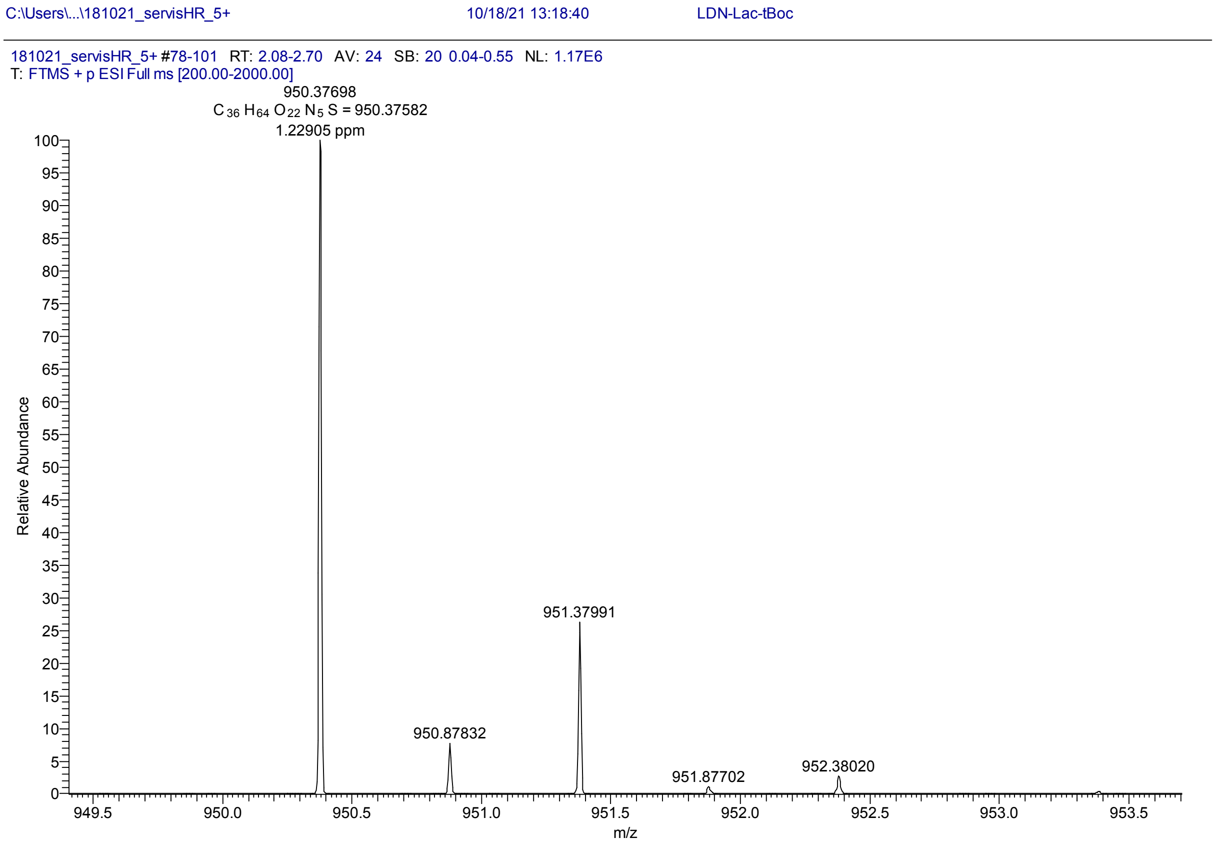

Figure S6d. High-resolution ESI-MS(+) of compound **5**. Calculated for $[M + H]^+$ ($C_{36}H_{64}N_5O_{22}S^+$) 950.37582, measured 950.37698 (1.23 ppm). The signals *m/z* 950.87832 and *m/z* 951.87702 correspond to doubly charged dimer ($[2M + 2H]^{2+}$) formed in the ion source.

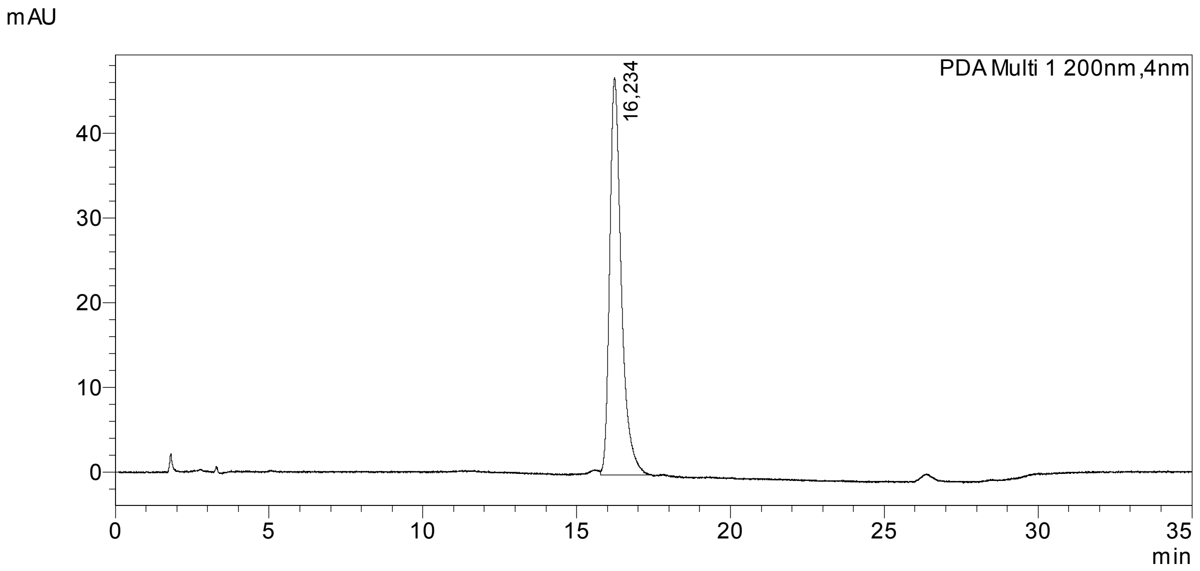

Figure S6e. HPLC chromatogram of compound **5** (retention time 16.234 min, purity 98%). Measured on a HILIC column.

LN2-*t*Boc (7)

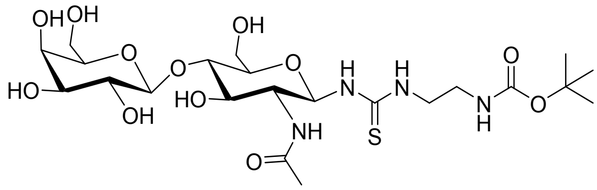

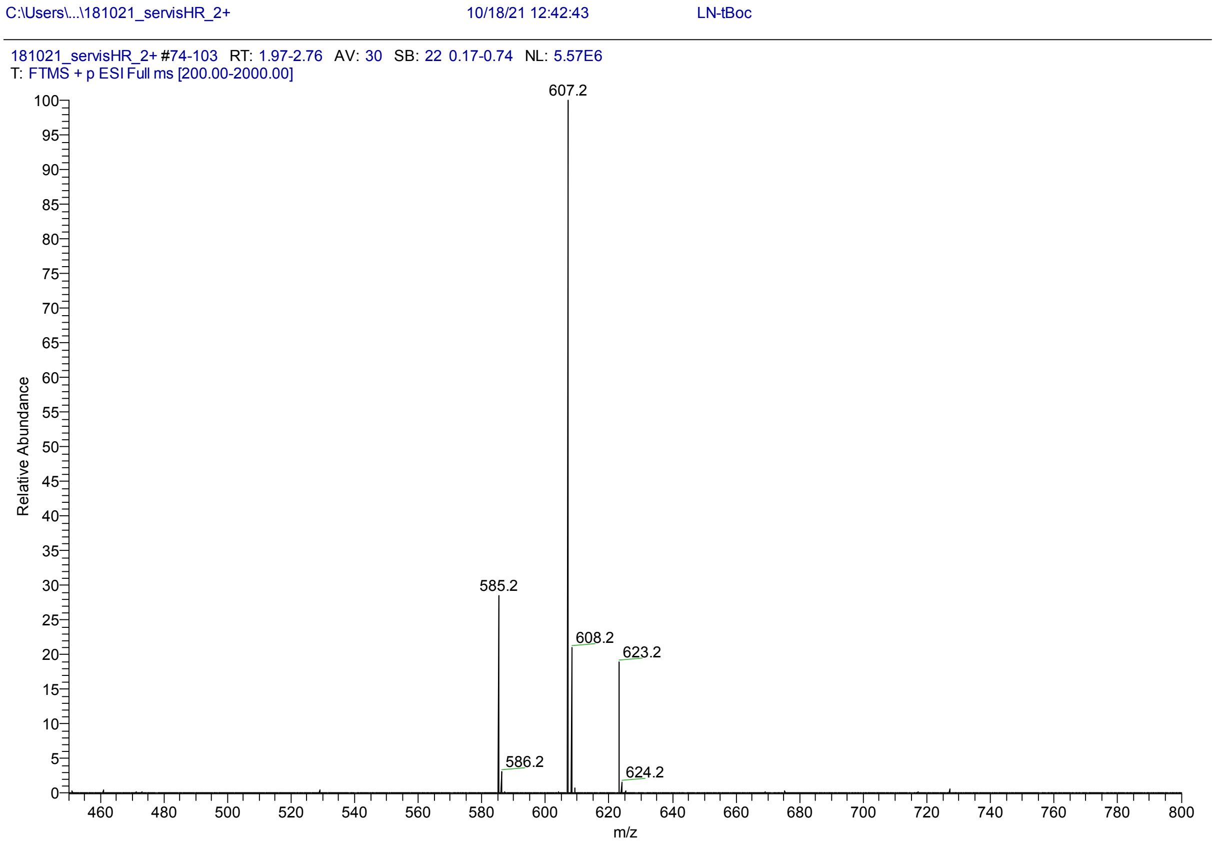

Figure S7a. ESI-MS(+) spectrum of compound **7**. $[M + H]^+$, *m/z* 585.2; $[M + Na]^+$, *m/z* 607.2; $[M + K]^+$, *m/z* 623.2.

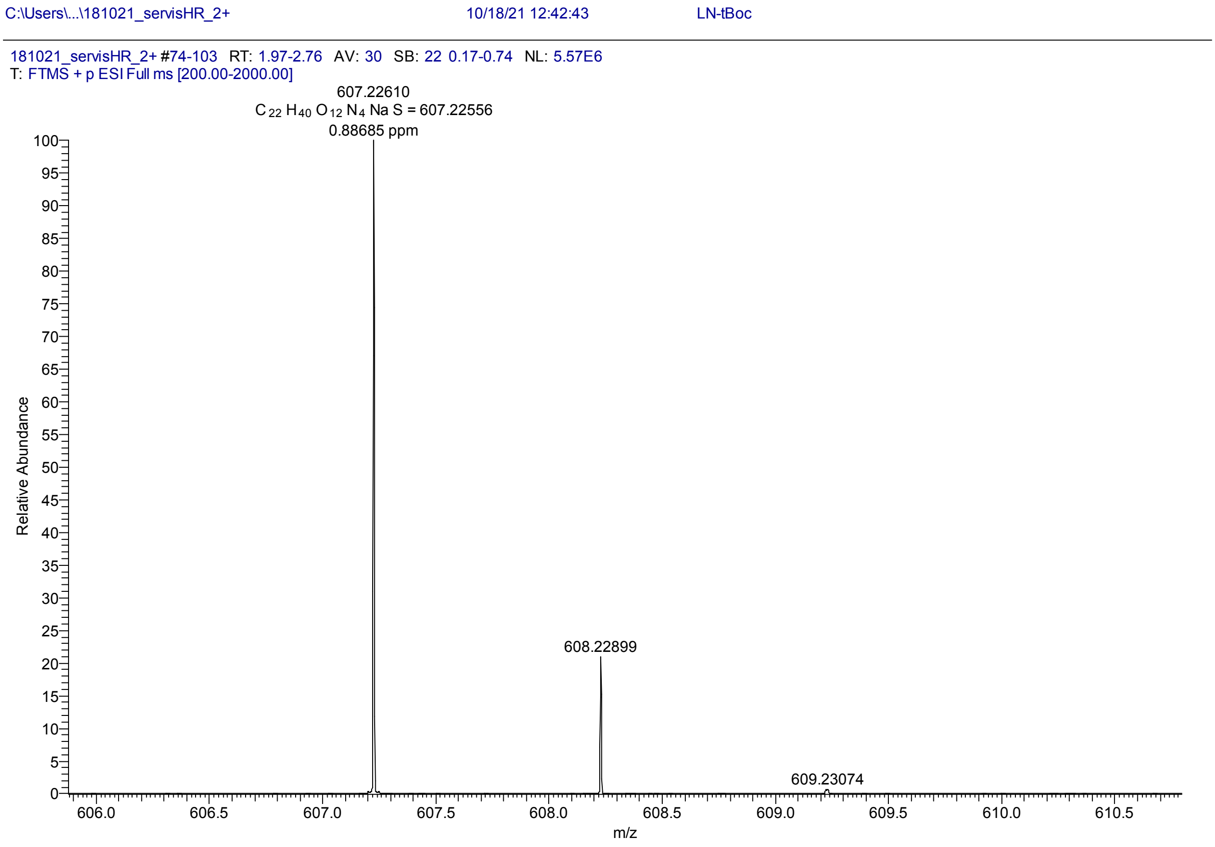

Figure S7b. High-resolution ESI-MS(+) of compound **7**. Calculated for $[M + Na]^+$ ($C_{22}H_{40}N_4O_{12}SNa^+$) 607.22556, measured 607.22610 (0.89 ppm).

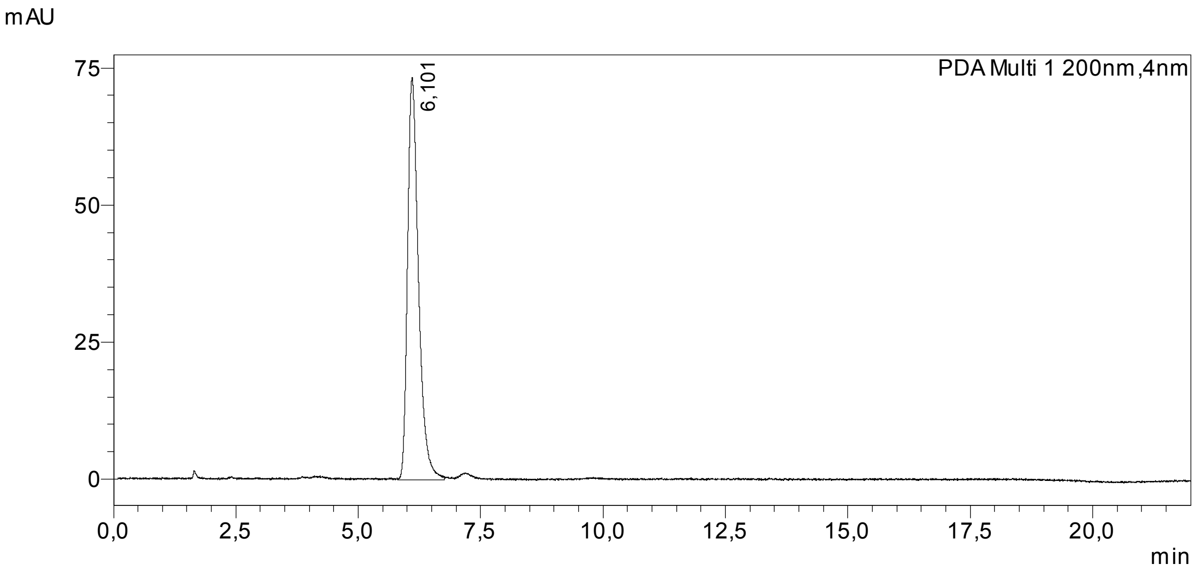

Figure S7c. HPLC chromatogram of compound **7** (retention time 6.101 min, purity 99%). Measured on a HILIC column.

GlcNAc-LN2-*t*Boc (8)

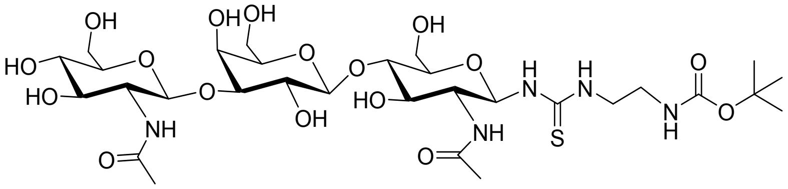

Table S6. ^{1}H and ^{13}C NMR data of compound **8** (700.13 MHz for ^{1}H, 176.05 MHz for ^{13}C, D_2O, 30 °C).

	Atom	δ_C	m.	δ_H	n_H	m.	*J* [Hz]	Diagnostic HMBC
**Boc**	**CO**	158.41	S	-	0	-		
	**C**	81.28	S	-	0	-		$(CH_3)_3$
	**$(CH_3)_3$**	27.88	Q	1.436	9	s		$(CH_3)_3$
**spacer**	**1'**	44.37	T	3.67^{H}	2	m		
	**2'**	39.50	T	3.269	2	br s		
	**CS**	n.d.	S	-	0	-		
**GlcNAc^{C}**	**1**	82.86^{x}	D	5.60, 5.50^{x}	1	br s		
	**2**	54.19	D	3.92^{H}	1	m		Ac
	**3**	72.82	D	3.806	1	br dd		
	**4**	78.27	D	3.74^{H}	1	m		1^{B}
	**5**	76.24	D	3.684	1	m		
	**6**	60.12	T	3.959	1	br dd		
				3.845	1	br dd	12.3, 4.4	
	**2-CO**	175.20	S	-	0	-		Ac
	**Ac**	22.23	Q	2.021	3	s		
**Gal^{B}**	**1**	103.10	D	4.484	1	d	7.8	
	**2**	70.22	D	3.608	1	dd	9.9, 7.8	
	**3**	82.11	D	3.734	1	dd	9.9, 3.3	1^{A}
	**4**	68.55	D	4.157	1	br d	3.3	
	**5**	75.10	D	3.73^{H}	1	m		
	**6**	61.15	T	3.76^{H}	2	m		
**GlcNAc^{A}**	**1**	103.02	D	4.697	1	d	8.5	3^{B}
	**2**	55.87	D	3.760	1	dd	10.4, 8.5	Ac
	**3**	73.78	D	3.574	1	dd	10.4, 8.4	
	**4**	69.92	D	3.478	1	dd	9.8, 8.4	
	**5**	75.87	D	3.448	1	ddd	9.8, 5.1, 2.1	
	**6**	60.71	T	3.903	1	dd	12.4, 2.1	
				3.767	1	dd	12.4, 5.1	
	**2-CO**	175.16	S	-	0	-		2, Ac
	**Ac**	22.37	Q	2.046	3	s		

^{x} … tentative assignment; ^{H} … HSQC readout; n.d. … not detected

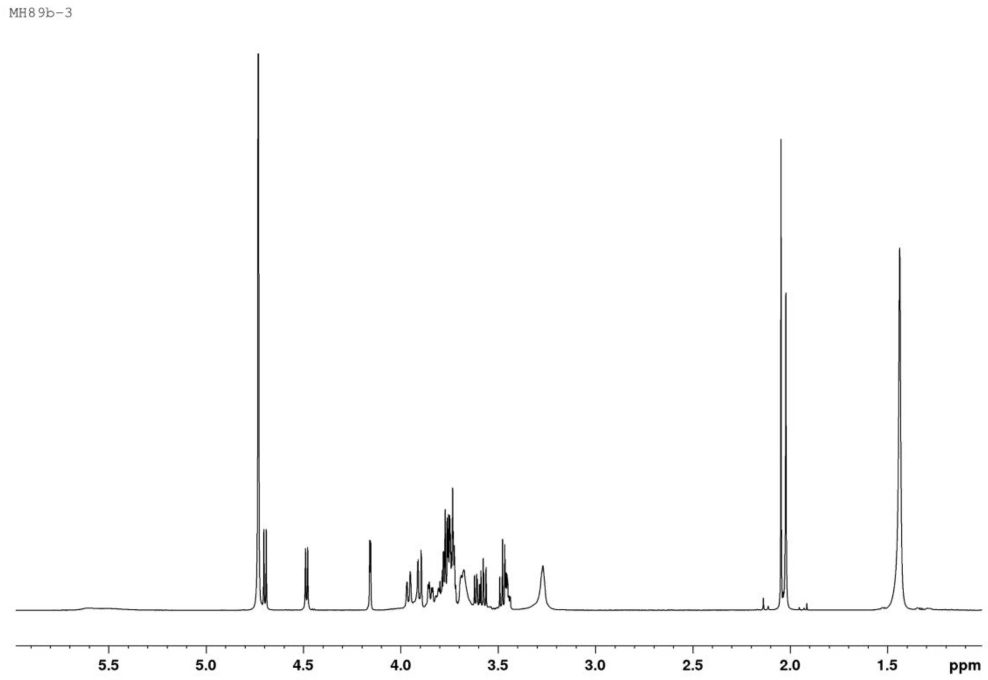

Figure S8a. ^{1}H NMR spectrum of compound **8** (700.13 MHz, D_2O, 30 °C).

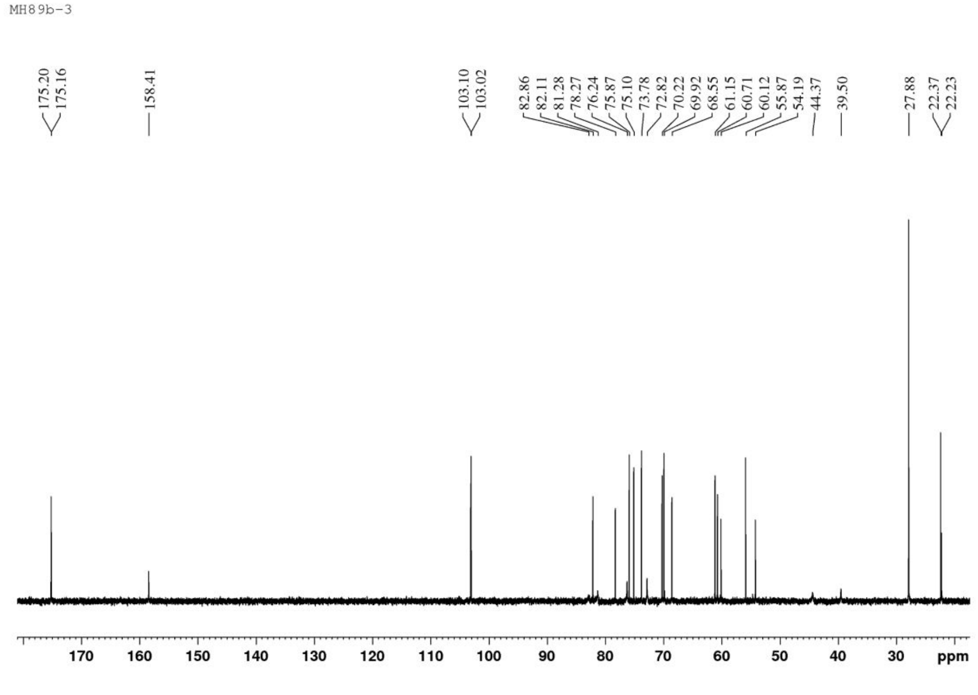

Figure S8b. ^{13}C NMR spectrum of compound **8** (176.05 MHz, D_2O, 30 °C).

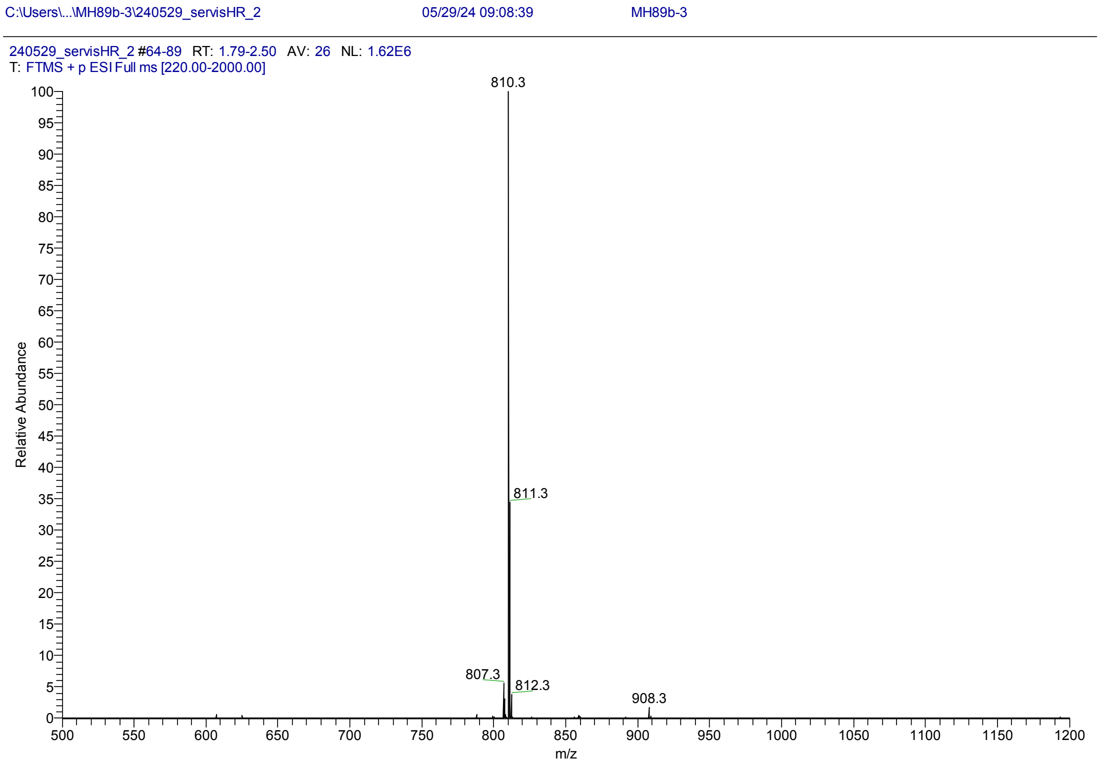

Figure S8c. ESI-MS(+) spectrum of compound **8**. $[M + Na]^+$, *m/z* 810.3.

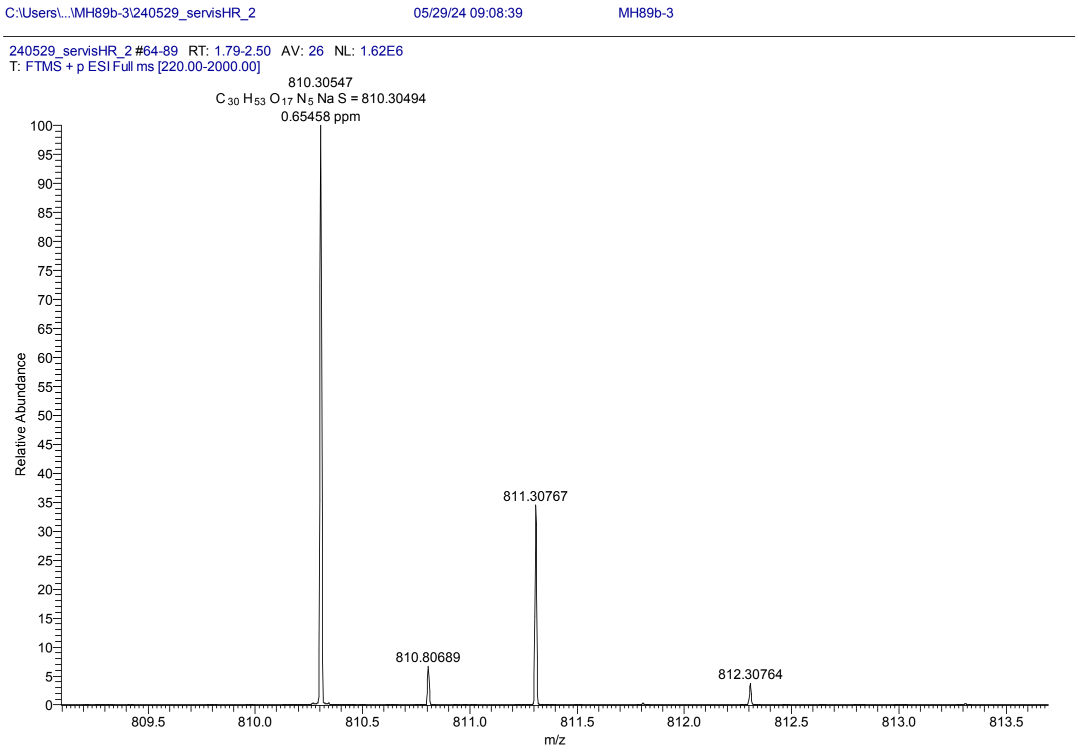

Figure S8d. High-resolution ESI-MS(+) of compound **8**. Calculated for $[M + Na]^+$ ($C_{30}H_{53}N_5O_{17}SNa^+$) 810.30494, measured 810.30547 (0.65 ppm). The signal *m/z* 810.80689 corresponds to doubly charged dimer ($[2M + 2Na]^{2+}$) formed in the ion source.

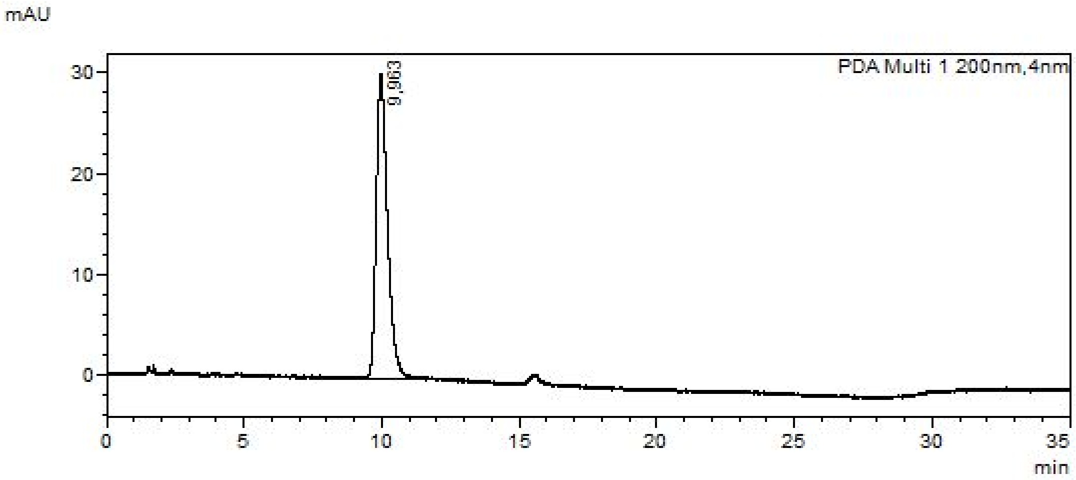

Figure S8e. HPLC chromatogram of compound **8** (retention time 9.963 min, purity 98%). Measured on a HILIC column.

LN1-LN2-*t*Boc (9)

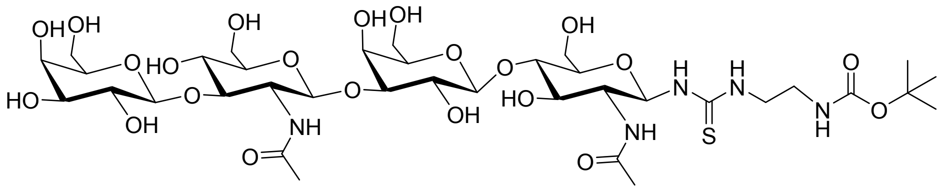

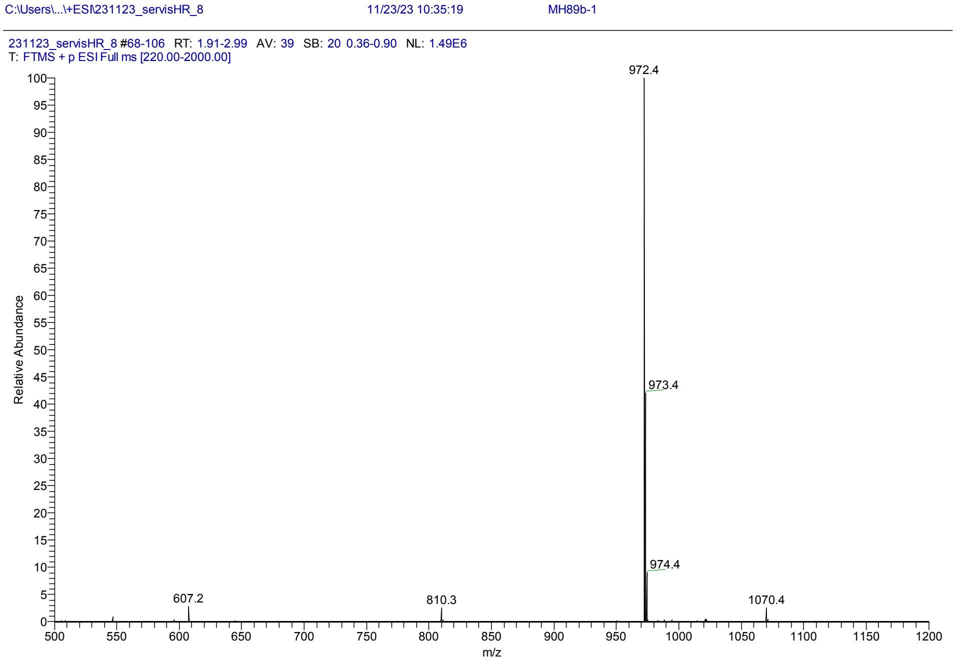

Figure S9a. ESI-MS(+) spectrum of compound **9**. $[M + Na]^+$, *m/z* 972.4.

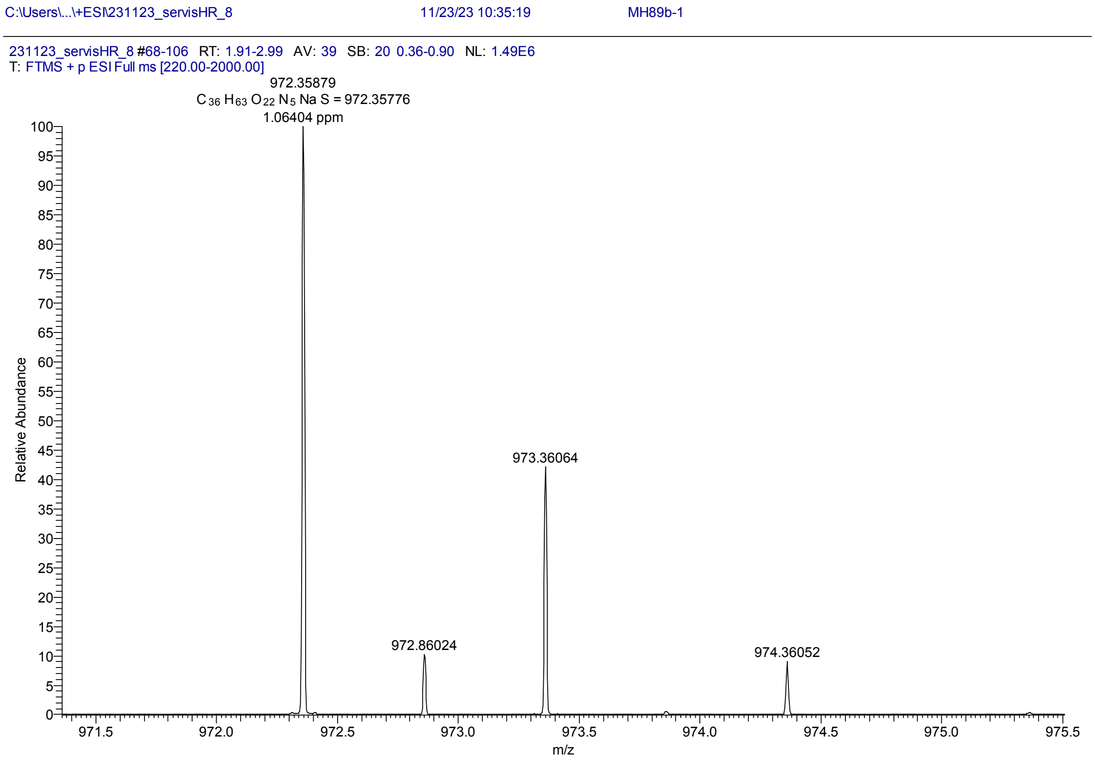

Figure S9b. High-resolution ESI-MS(+) of compound **9**. Calculated for $[M + Na]^+$ ($C_{36}H_{63}N_5O_{22}SNa^+$) 972.35776, measured 972.35879 (1.06 ppm). The signal *m/z* 972.86024 corresponds to doubly charged dimer ($[2M + 2Na]^{2+}$) formed in the ion source.

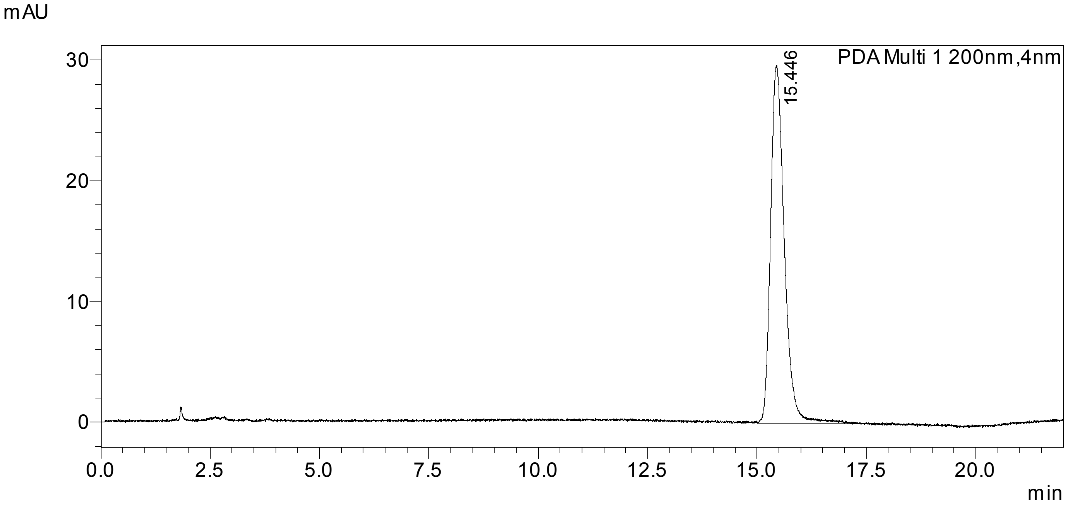

Figure S9c. HPLC chromatogram of compound **9** (retention time 15.446 min, purity 99%). Measured on a HILIC column.

LN2-LN2-*t*Boc (10)

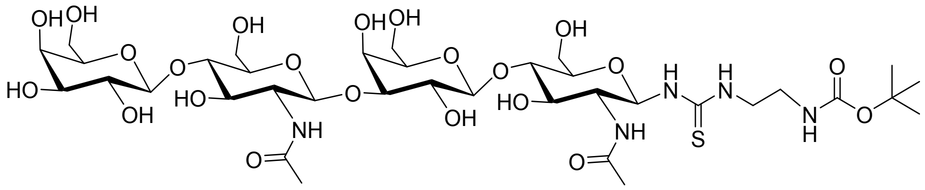

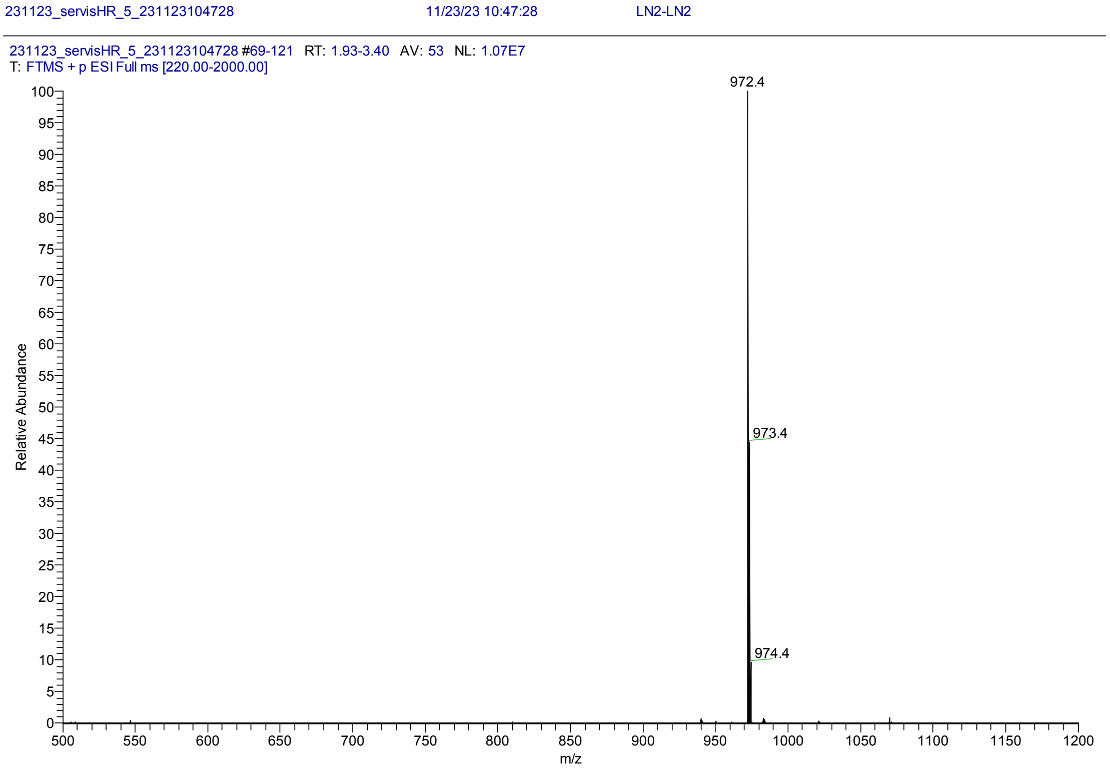

Figure S10a. ESI-MS(+) spectrum of compound **10**. $[M + Na]^+$, *m/z* 972.4.

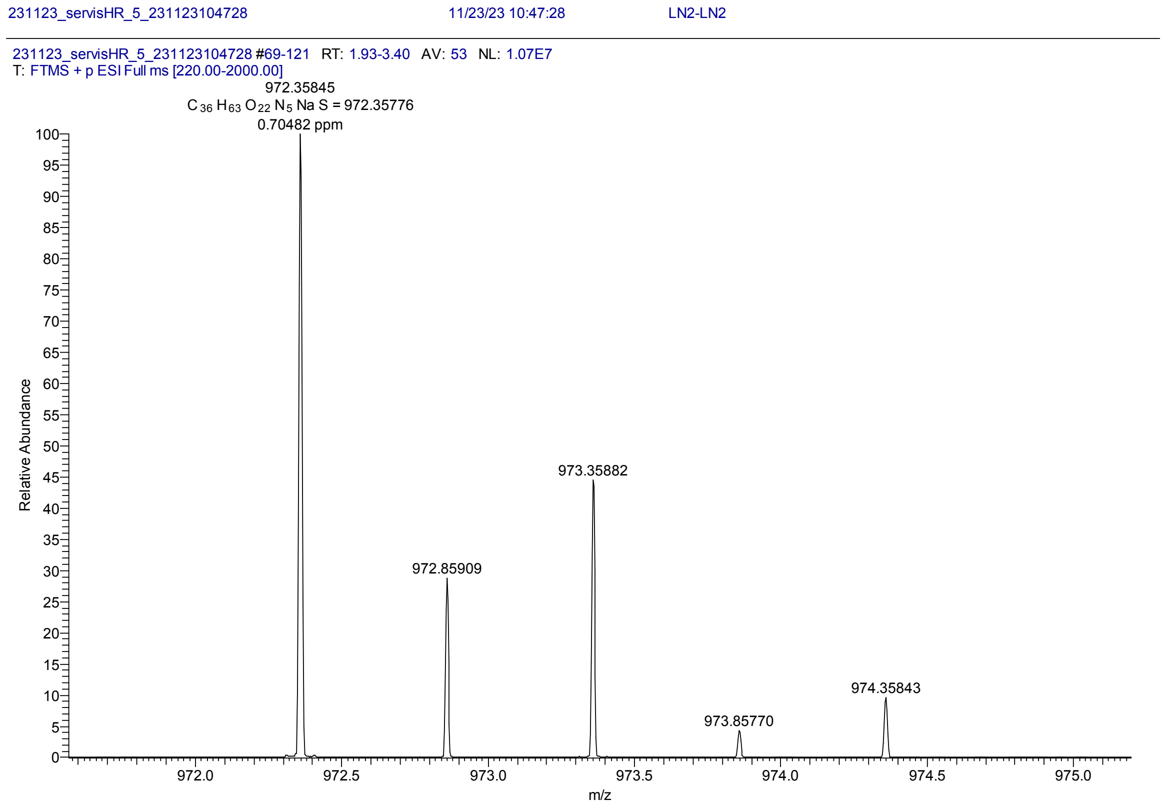

Figure S10b. High-resolution ESI-MS(+) of compound **10**. Calculated for $[M + Na]^+$ ($C_{36}H_{63}N_5O_{22}SNa^+$) 972.35776, measured 972.35845 (0.70 ppm). The signals *m/z* 972.85909 and *m/z* 973.85770 correspond to doubly charged dimer ($[2M + 2Na]^{2+}$) formed in the ion source.

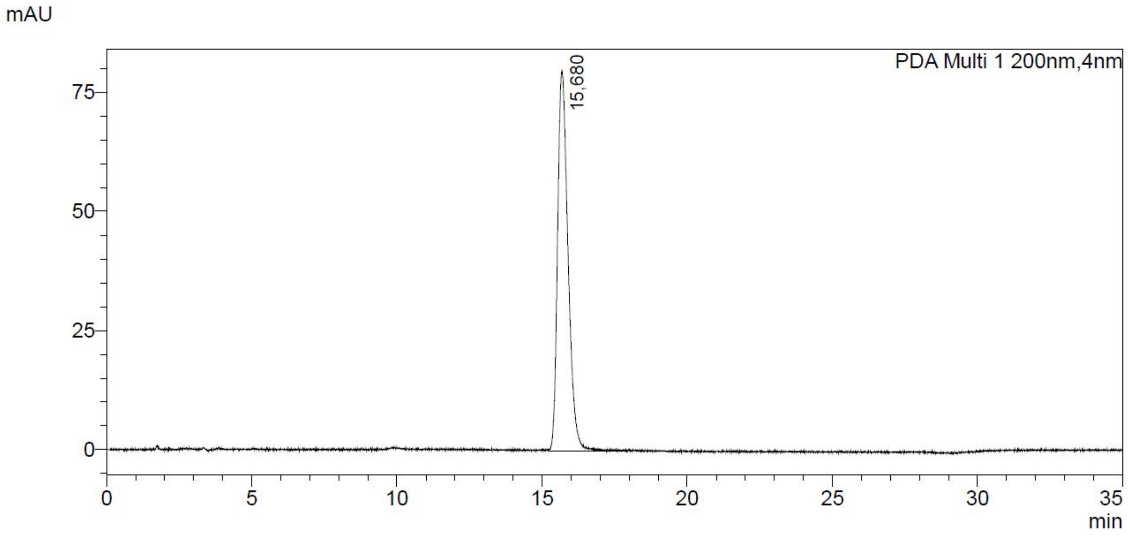

Figure S10c. HPLC chromatogram of compound **10** (retention time 15.680 min, purity 99%). Measured on a HILIC column.

LDN-LN2-*t*Boc (11)

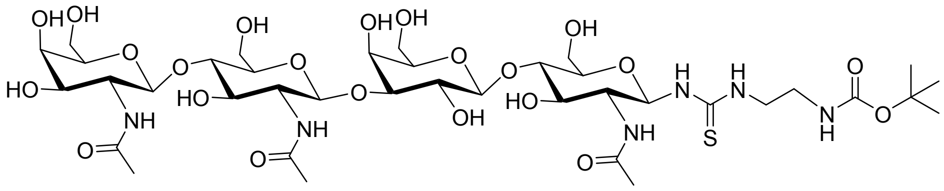

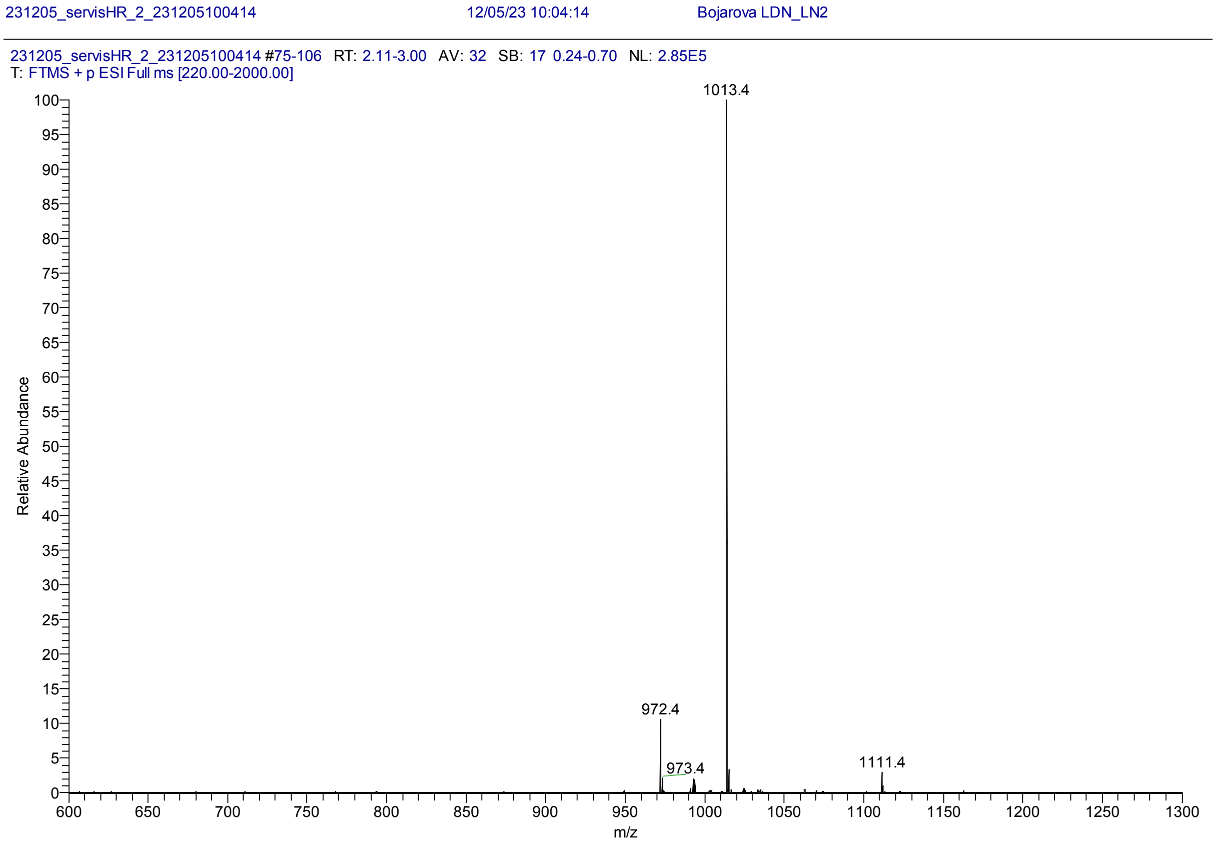

Figure S11a. ESI-MS(+) spectrum of compound **11**. $[M + Na]^+$, *m/z* 1013.4.

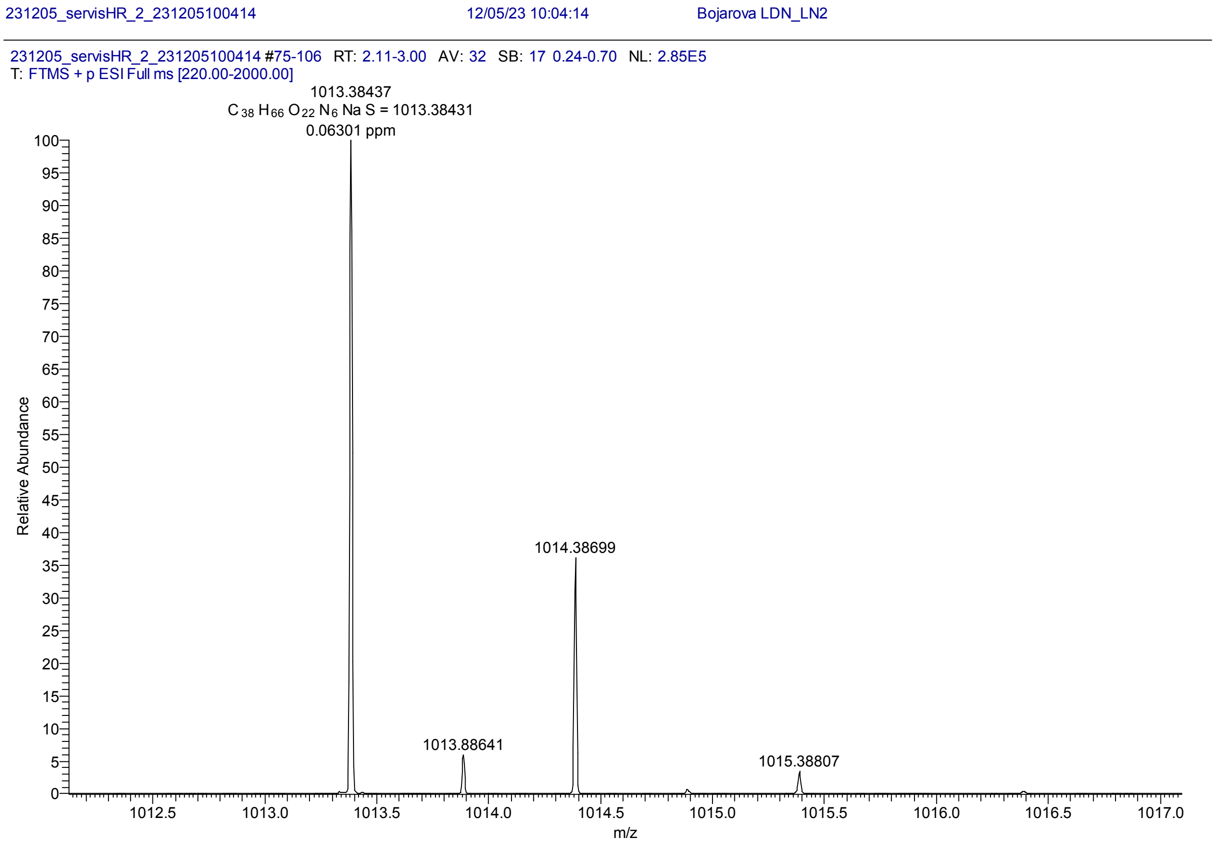

Figure S11b. High-resolution ESI-MS(+) of compound **11**. Calculated for $[M + Na]^+$ ($C_{38}H_{66}N_6O_{22}SNa^+$) 1013.38431, measured 1013.38437 (0.06 ppm). The signal *m/z* 1013.88641 corresponds to doubly charged dimer ($[2M + 2Na]^{2+}$) formed in the ion source.

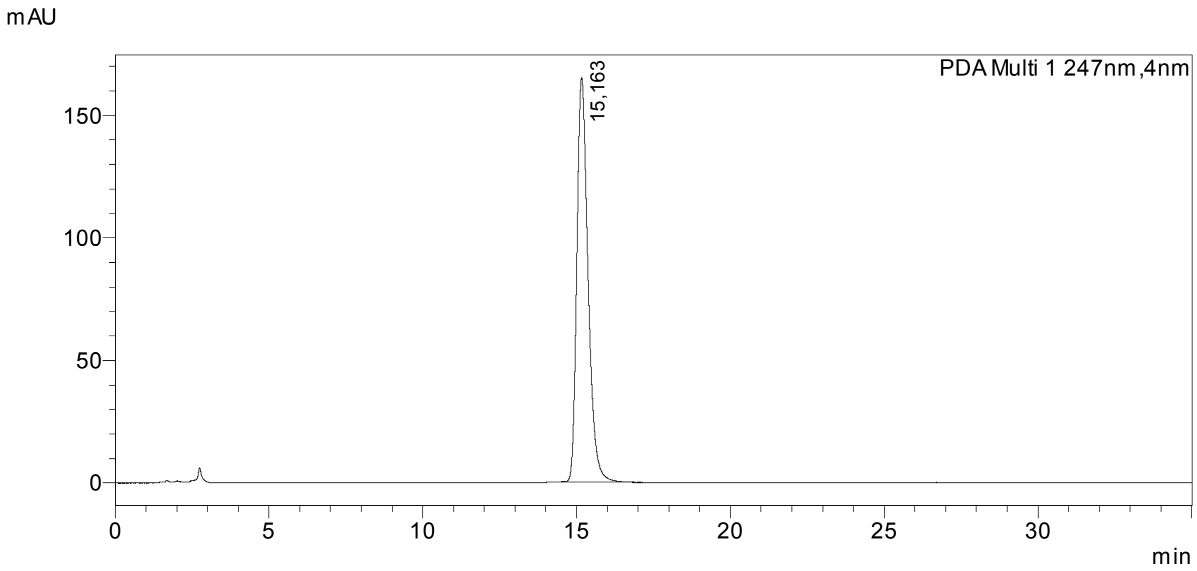

Figure S11c. HPLC chromatogram of compound **11** (retention time 15.163 min, purity 98%). Measured on a HILIC column.

5.2. Squarates

Lac-Sq (1a)

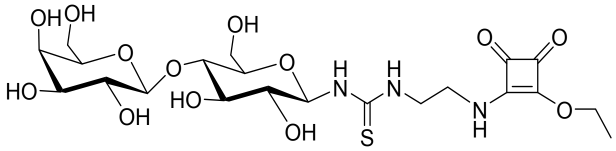

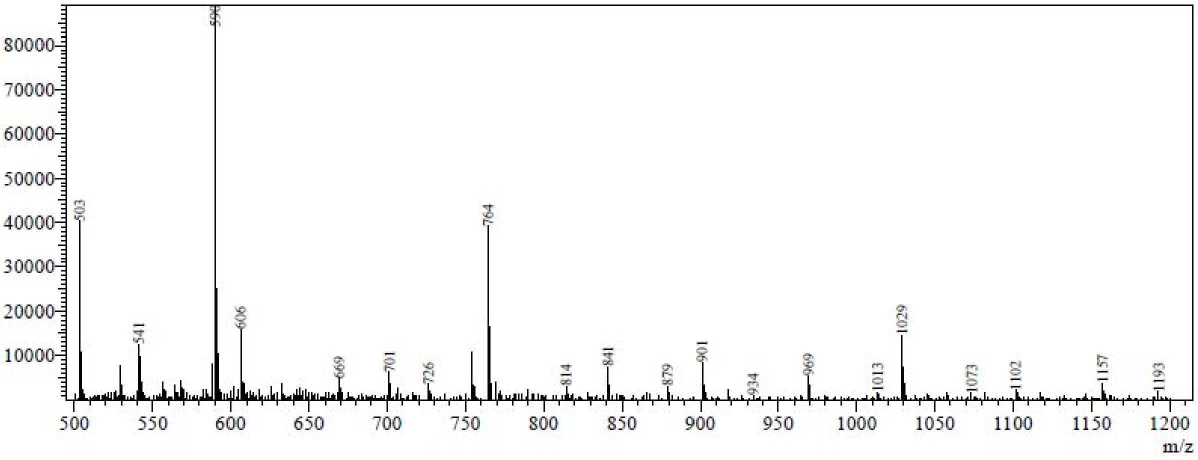

Figure S12a. ESI-MS(+) spectrum of compound **1a** (exact mass: 567.17; detected mass: $[M + Na]^+$, *m/z* 590).

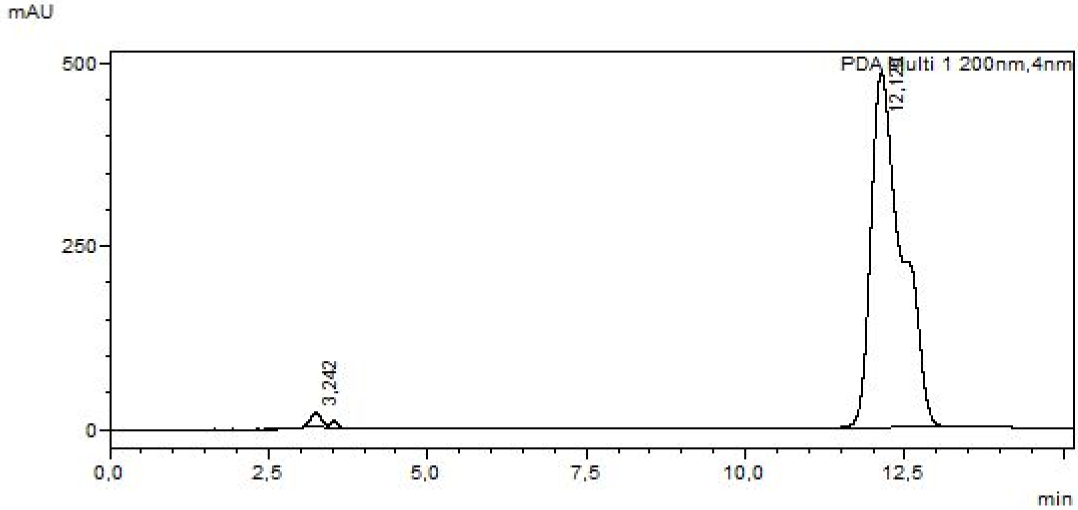

Figure S12b. HPLC chromatogram of compound **1a** (retention time 12.129 min, purity 98%). Measured on a C18 column.

LN1-Lac-Sq (3a)

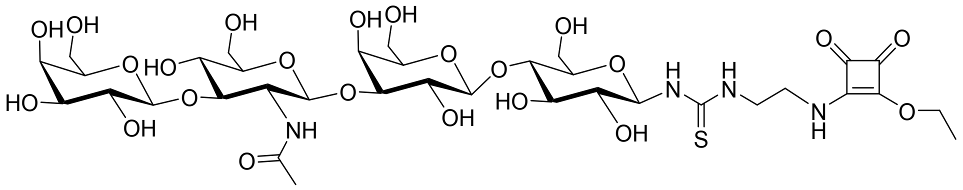

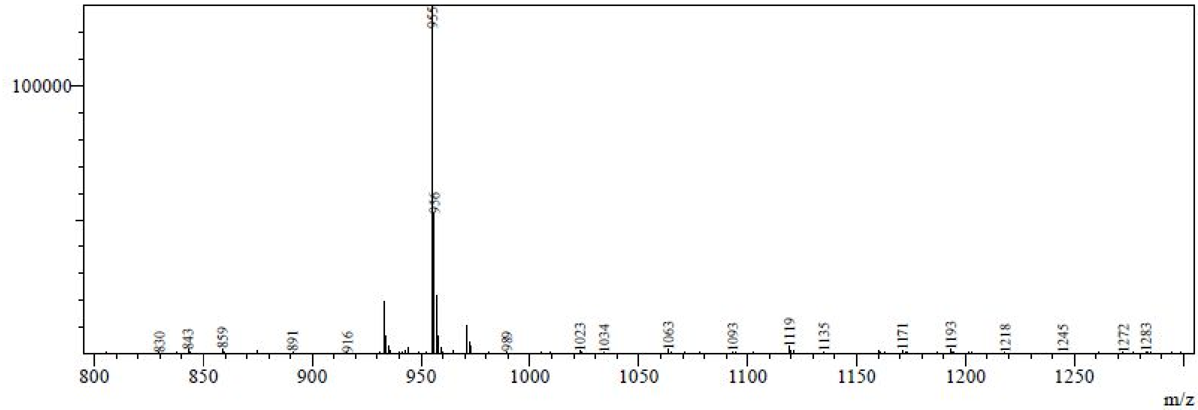

Figure S13a. ESI-MS(+) spectrum of compound **3a** (exact mass: 932.31; detected mass: $[M + Na]^+$, *m/z* 955).

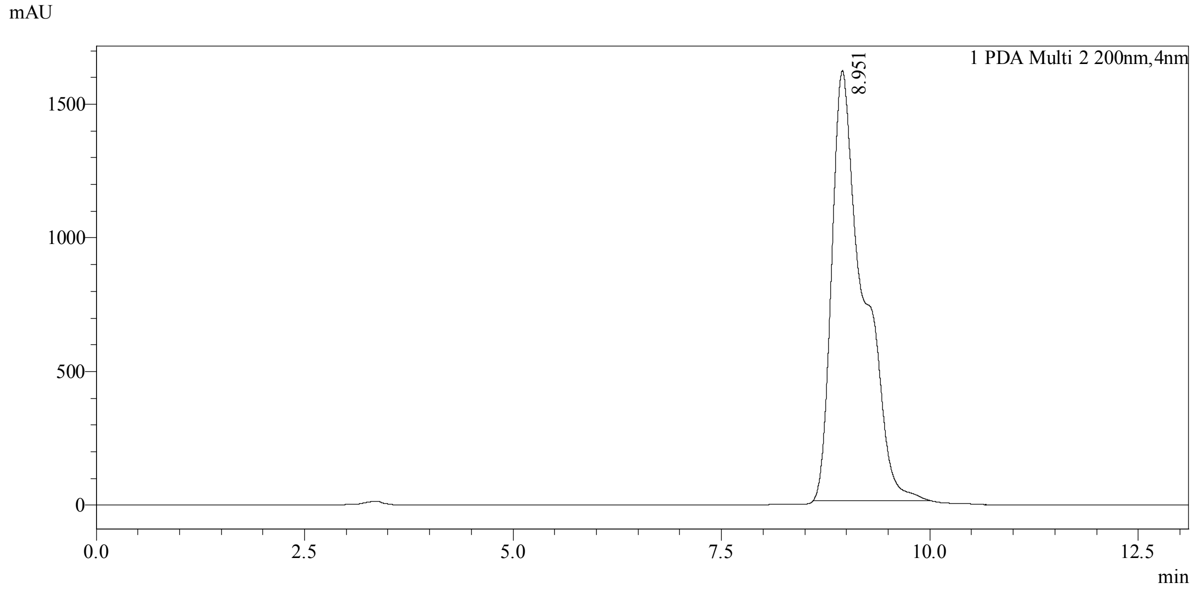

Figure S13b. HPLC chromatogram of compound **3a** (retention time 8.951 min, purity 98%). Measured on a C18 column.

LN2-Lac-Sq (4a)

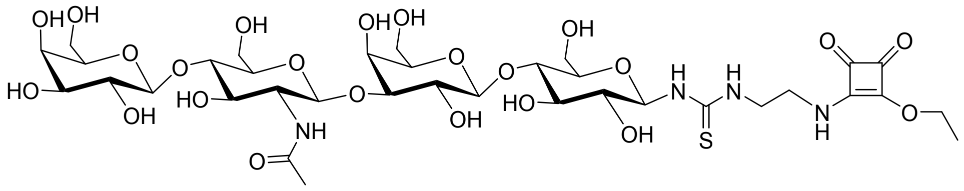

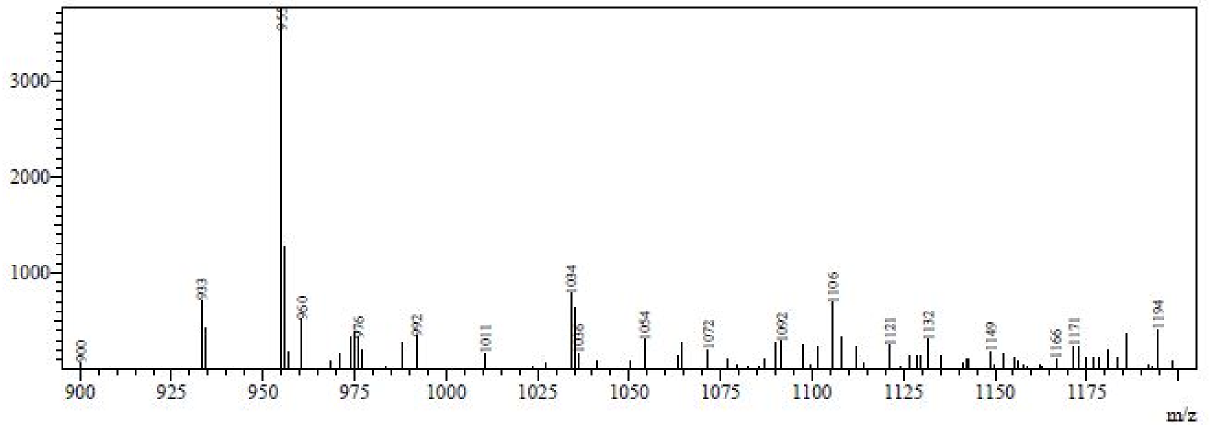

Figure S14a. ESI-MS(+) spectrum of compound **4a** (exact mass: 932.31; detected mass: $[M + H]^+$, *m/z* 933; $[M + Na]^+$, *m/z* 955).

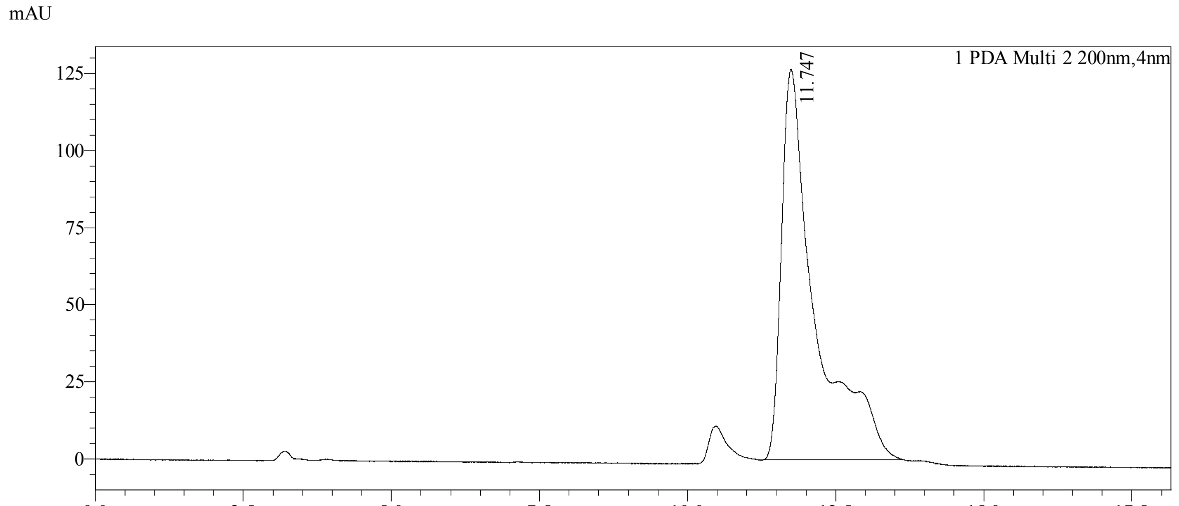

Figure S14b. HPLC chromatogram of compound **4a** (retention time 11.747 min, purity 94%). Measured on a C18 column.

LDN-Lac-Sq (5a)

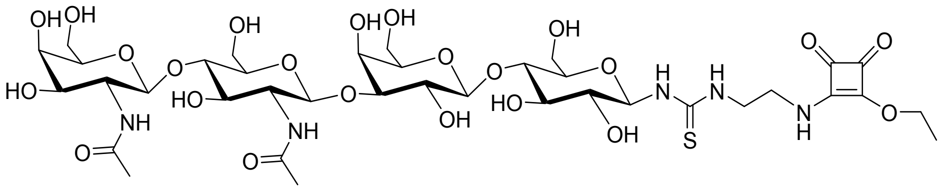

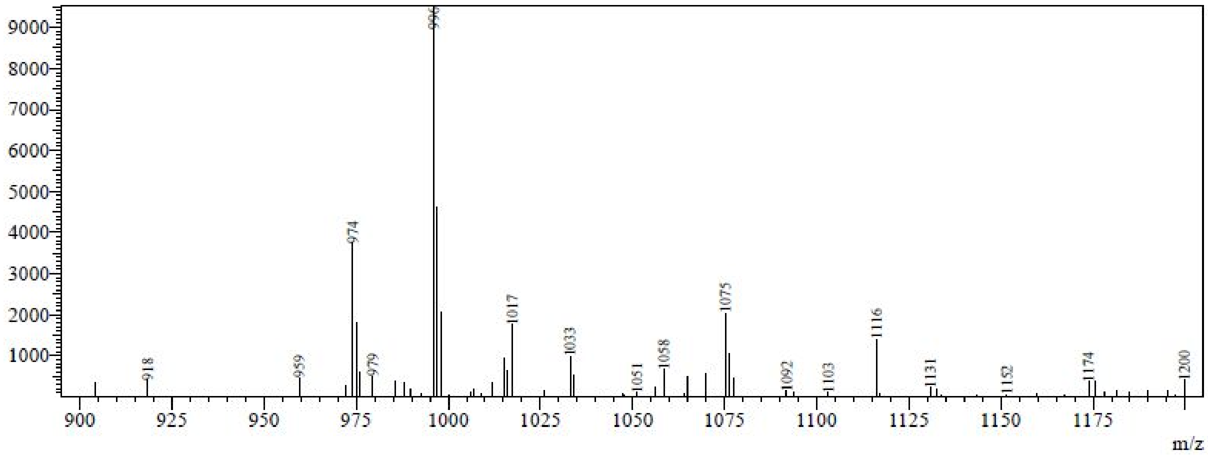

Figure S15a. ESI-MS(+) spectrum of compound **5a** (exact mass: 973.33; detected mass: $[M + H]^+$, *m/z* 974; $[M + Na]^+$, *m/z* 996).

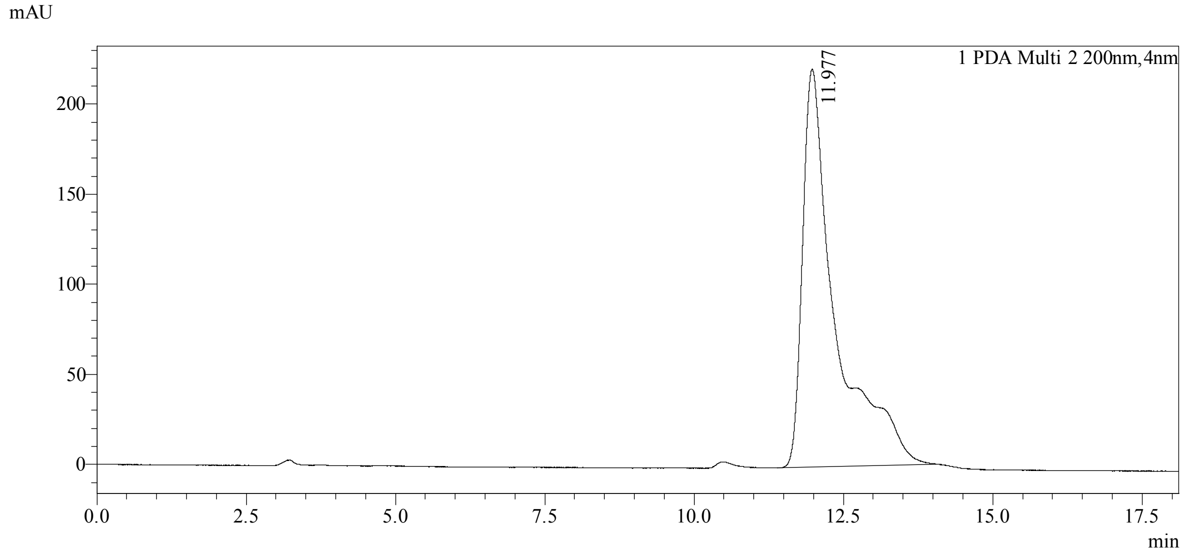

Figure S15b. HPLC chromatogram of compound **5a** (retention time 11.977 min, purity 98%). Measured on a C18 column.

LN2-Sq (7a)

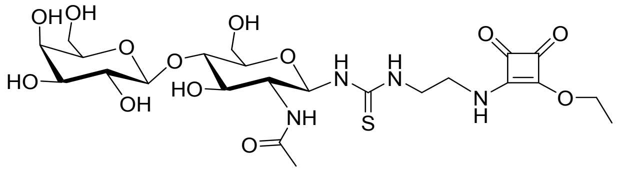

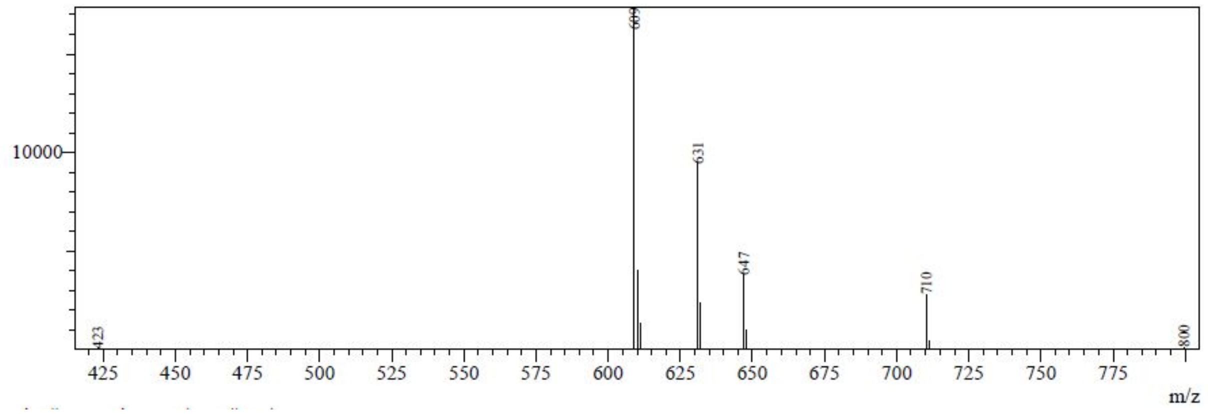

Figure S16a. ESI-MS(+) spectrum of compound **7a** (exact mass: 608.20; detected mass: $[M + H]^+$, *m/z* 609; $[M + Na]^+$, *m/z* 631).

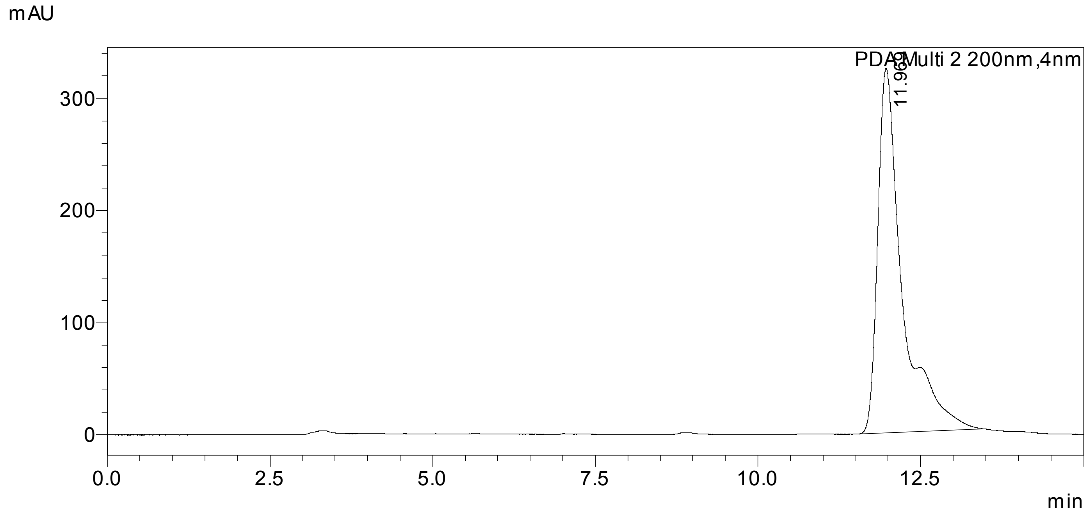

Figure S16b. HPLC chromatogram of compound **7a** (retention time 11.960 min, purity 98%). Measured on a C18 column.

LN1-LN2-Sq (9a)

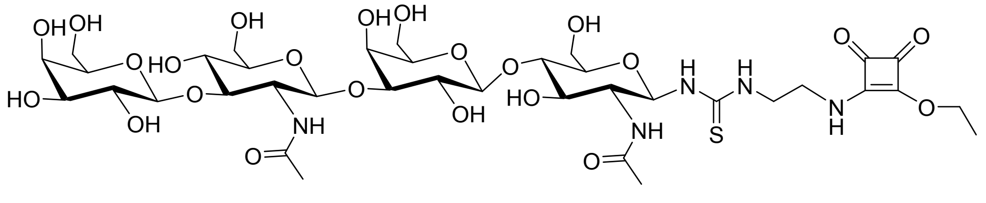

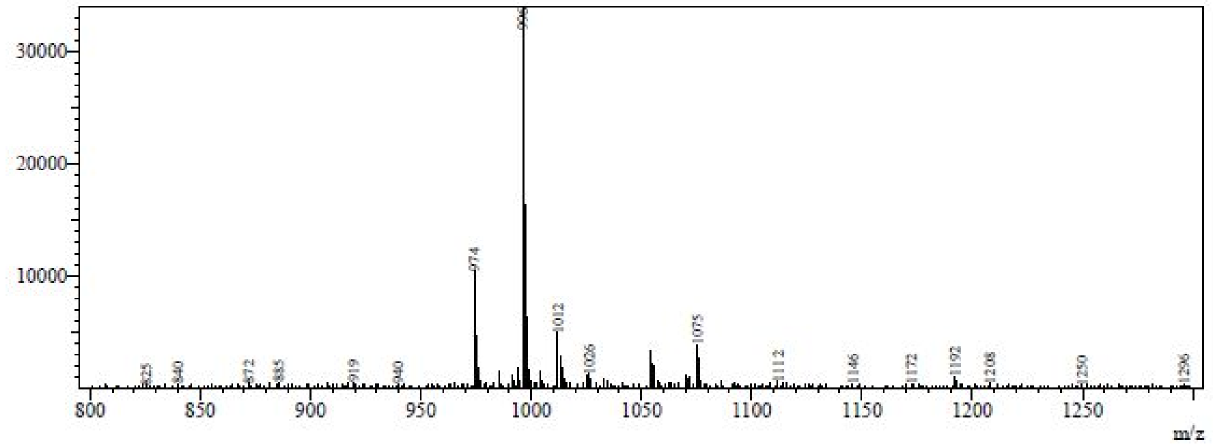

Figure S17a. ESI-MS(+) spectrum of compound **9a** (exact mass: 973.33; detected mass: $[M + H]^+$, *m/z* 974; $[M + Na]^+$, *m/z* 996).

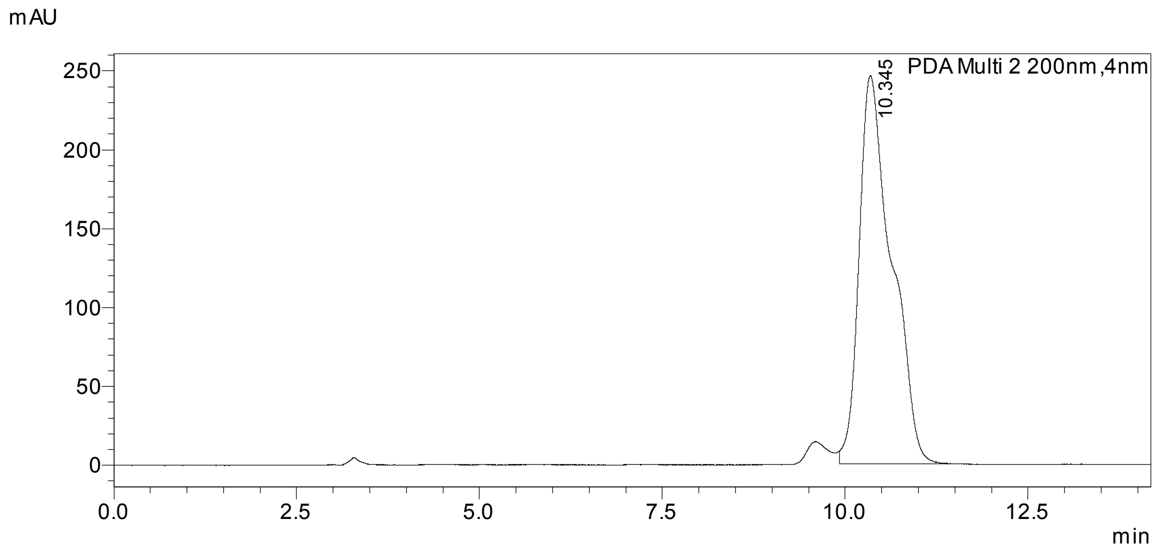

Figure S17b. HPLC chromatogram of compound **9a** (retention time 10.345 min, purity 96%). Measured on a C18 column.

LN2-LN2-Sq (10a)

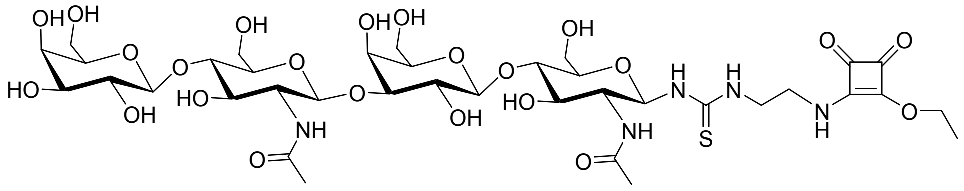

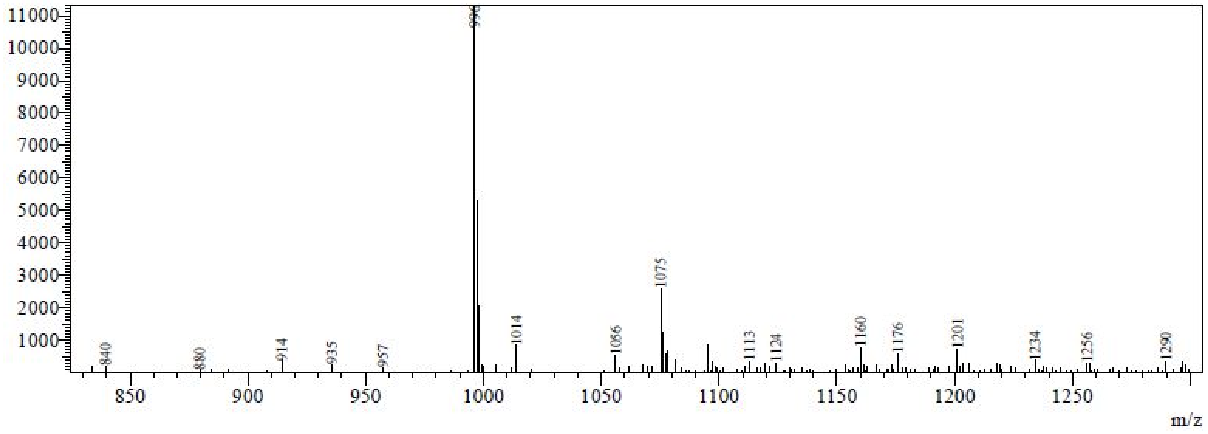

Figure S18a. ESI-MS(+) spectrum of compound **10a** (exact mass: 973.33; detected mass: $[M + Na]^+$, *m/z* 996).

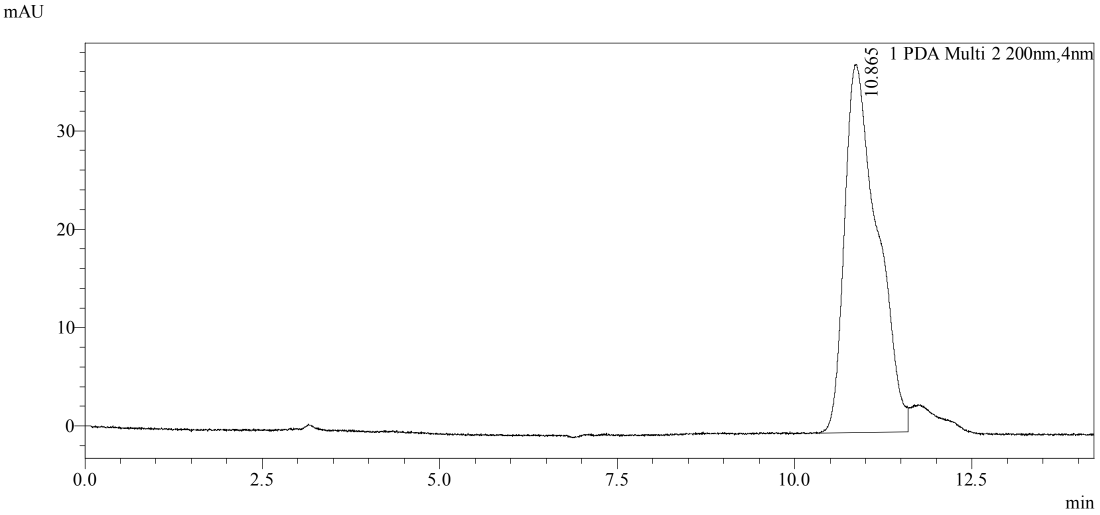

Figure S18b. HPLC chromatogram of compound **10a** (retention time 10.865 min, purity 93%). Measured on a C18 column.

LDN-LN2-Sq (11a)

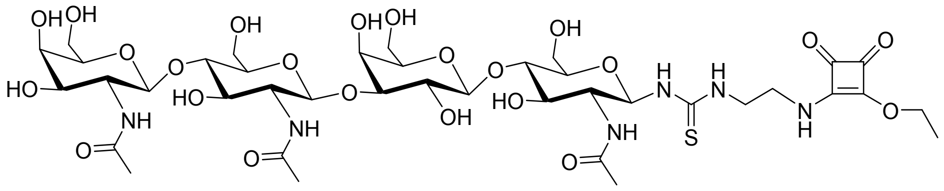

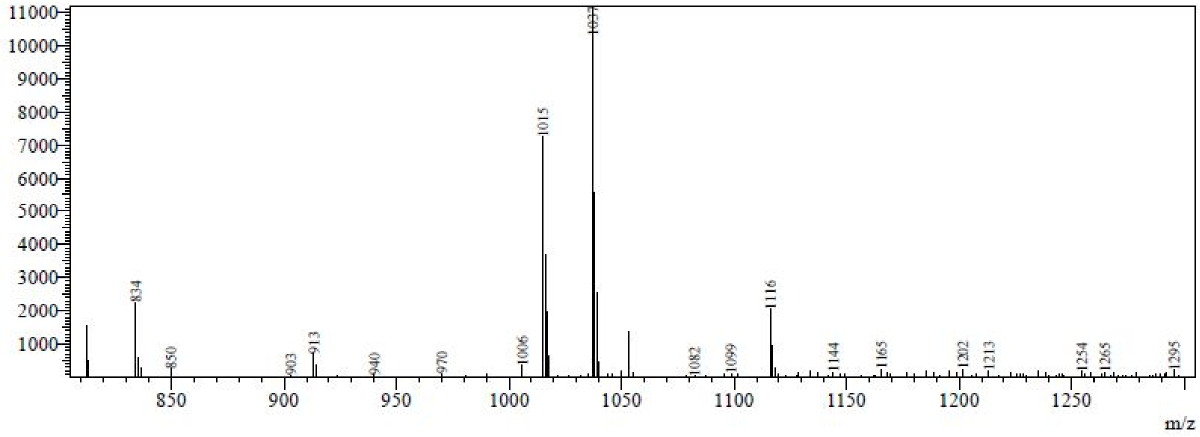

Figure S19a. ESI-MS(+) spectrum of compound **11a** (exact mass: 1014.36; detected mass: $[M + H]^+$, *m/z* 1015; $[M + Na]^+$, *m/z* 1037).

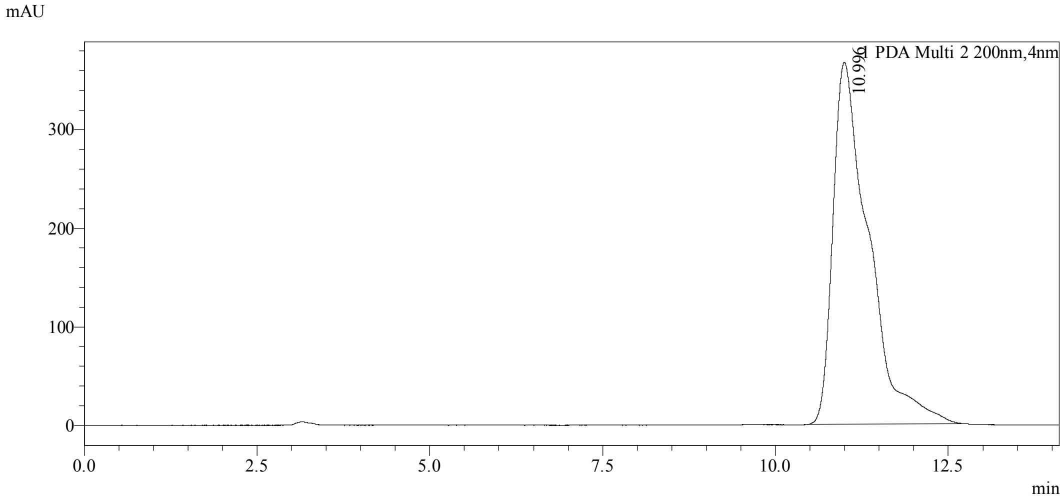

Figure S19b. HPLC chromatogram of compound **11a** (retention time 10.996 min, purity 99%). Measured on a C18 column.

5.3. Neo-Glycoproteins

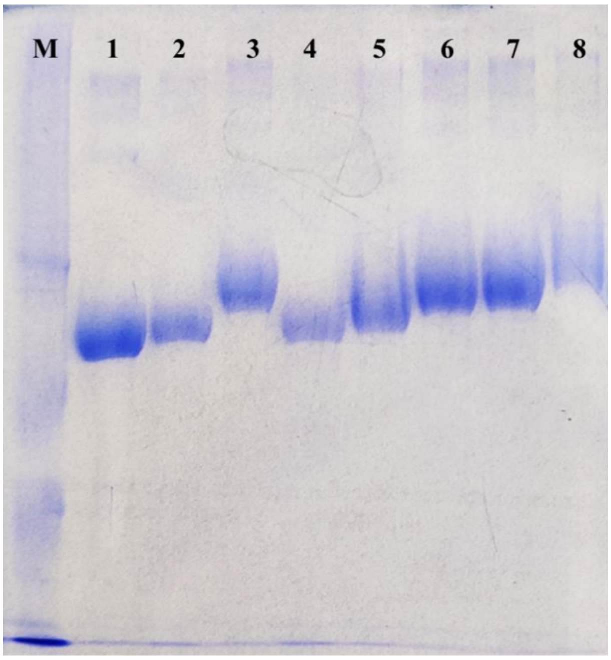

Figure S20. SDS-PAGE of neo-glycoproteins and HSA as starting material. **M**, Low molecular weight marker (GE Healthcare). Numbers refer to the neo-glycoproteins carrying respective glycans: **NGP1** carrying Lac (**1**); **NGP2** carrying LN2 (**7**); **NGP3** carrying LN1-Lac (**3**); **NGP4** carrying LN1-LN2 (**9**); **NGP5** carrying LN2-Lac (**4**); **NGP6** carrying LN2-LN2 (**10**); **NGP7** carrying LDN-Lac (**5**); **NGP8** carrying LDN-LN2 (**11**).

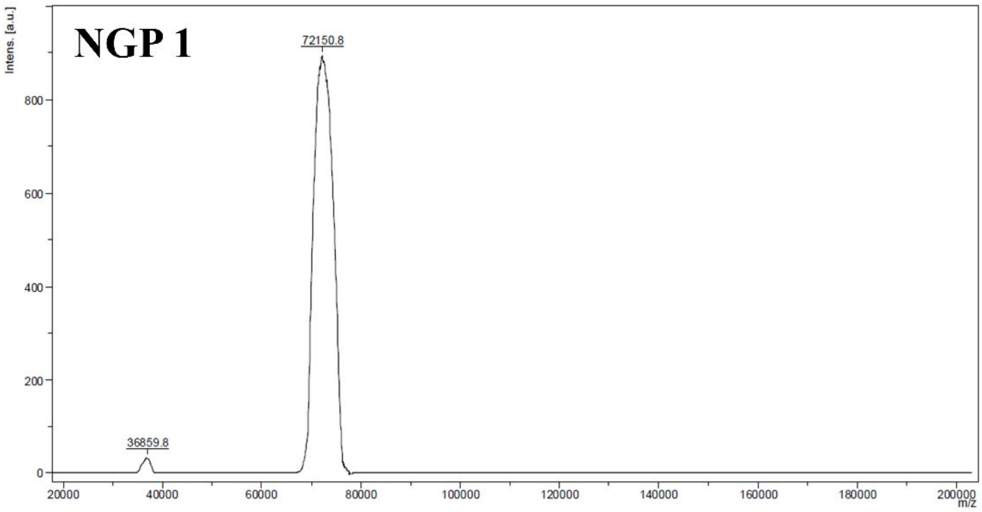

Figure S21. MALDI-TOF spectrum of neo-glycoprotein **NGP1** carrying Lac (**1**).

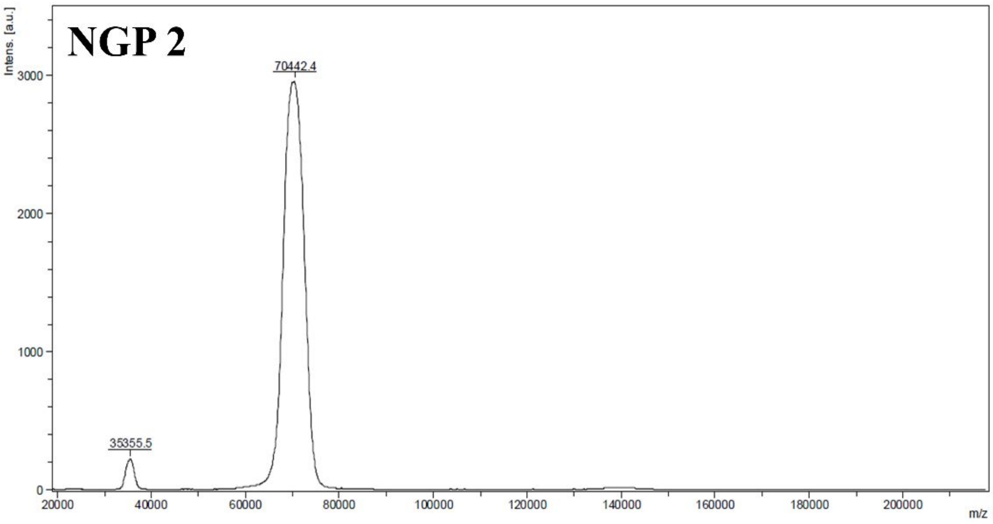

Figure S22. MALDI-TOF spectrum of neo-glycoprotein **NGP2** carrying LacNAc (**7**).

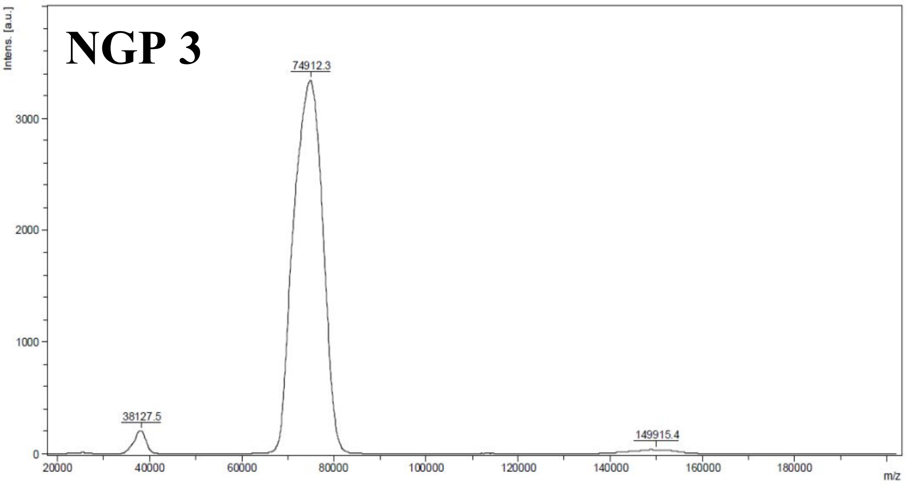

Figure S23. MALDI-TOF spectrum of neo-glycoprotein **NGP3** carrying LN1-Lac (**3**).

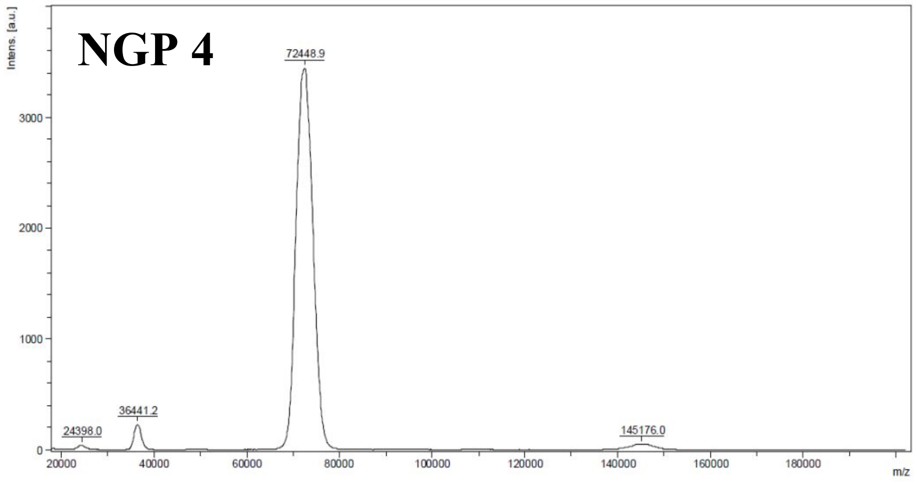

Figure S24. MALDI-TOF spectrum of neo-glycoprotein **NGP4** carrying LN1-LN2 (**9**).

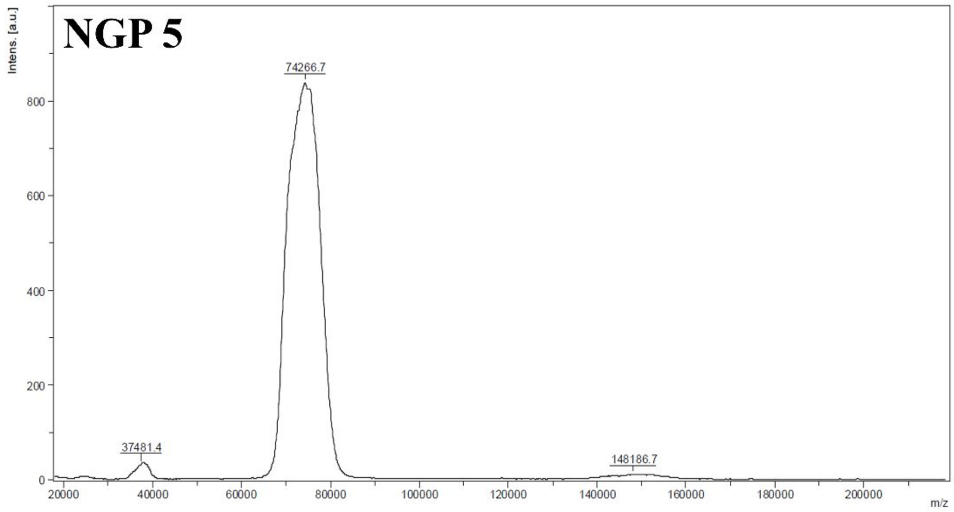

Figure S25. MALDI-TOF spectrum of neo-glycoprotein **NGP5** carrying LN2-Lac (**4**).

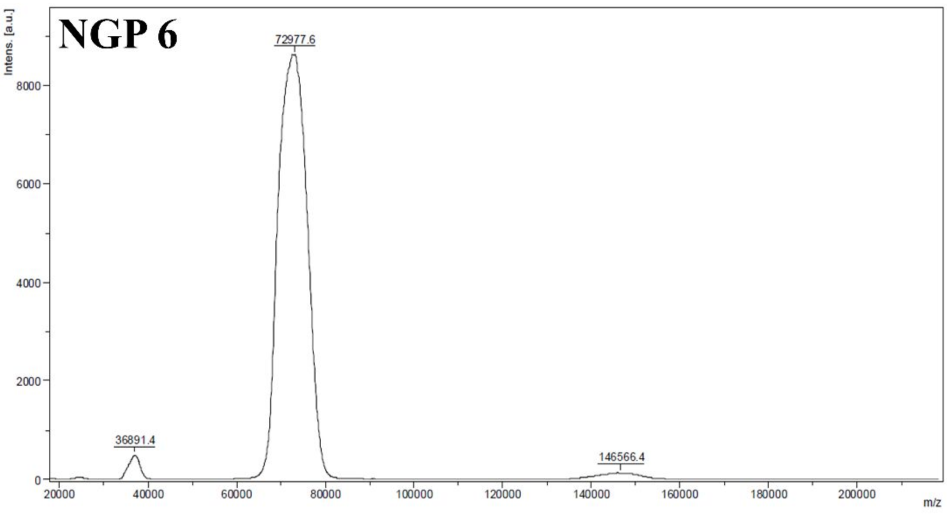

Figure S26. MALDI-TOF spectrum of neo-glycoprotein **NGP6** carrying LN2-LN2 (**10**).

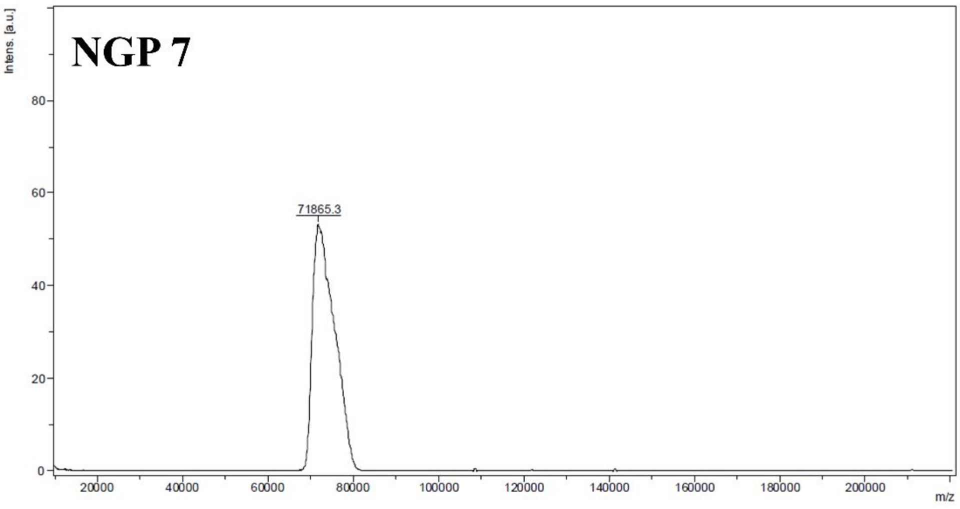

Figure S27. MALDI-TOF spectra of neo-glycoprotein **NGP7** carrying LDN-Lac (**5**).

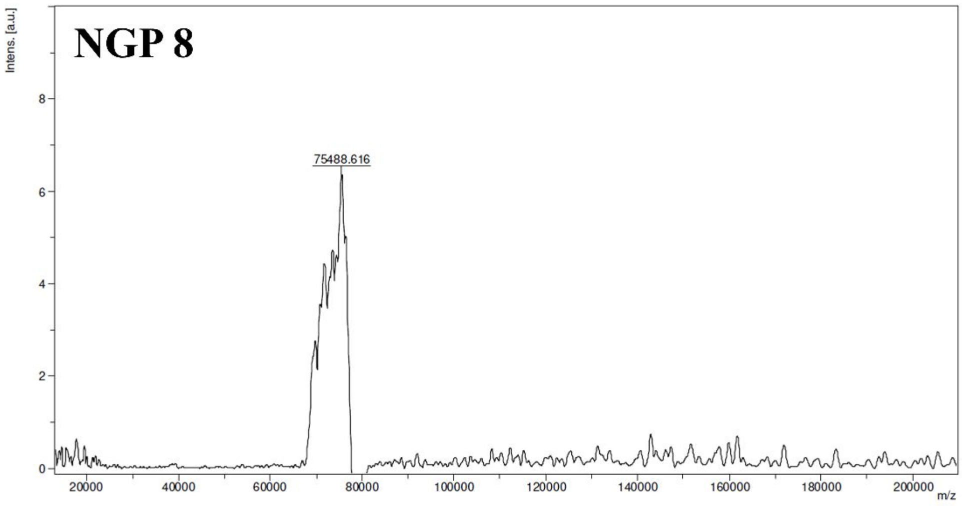

Figure S28. MALDI-TOF spectrum of neo-glycoprotein **NGP8** carrying LDN-LN2 (**11**).

Table S7. Glycan content of neo-glycoproteins determined by MALDI-TOF. Molecular weight of HSA was subtracted from the NGP molecular weight measured by MALDI-TOF. The difference was divided by the molecular weight of the respective squarate monoamide.

Neo-glycoprotein	Squarate monoamide	M_w of squarate monoamide	M_w of all attached glycans	No. of glycans
**NGP1**	**Lac-Sq (1a)**	567.0	5455.8	9.7
**NGP2**	**LN2-Sq (7a)**	608.0	3721.6	6.1
**NGP3**	**LN1-Lac-Sq (3a)**	932.3	9181.5	8.8
**NGP4**	**LN1-LN2-Sq (9a)**	973.3	5753.1	5.9
**NGP5**	**LN2-Lac-Sq (4a)**	932.3	7571.6	8.1
**NGP6**	**LN2-LN2-Sq (10a)**	973.3	6282.5	6.4
**NGP7**	**LDN-Lac-Sq (5a)**	973.3	5385.3	5.5
**NGP8**	**LDN-LN2-Sq (11a)**	1014.0	9009.0	8.9

6. Cloning and Production of Galectins

6.1. Cloning of Galectin Constructs

Recombinant human galectins (Gal-1, Gal-3, Gal-8, and Gal-9) for ELISA assays were produced as N-terminal His-tagged constructs in the pET-Duet1 vector, prepared as previously described.[8–11,14] The cloning sites comprised *Nco*I/*Asc*I for Gal-1 and Gal-3 (full length), and *Asc*I/*Not*I for Gal-8 and Gal-9. The Gal-1 construct carried a stabilizing C2S mutation to prevent protein spontaneous oxidation).[15] Gal-4 was cloned in pET28a vector (restriction sites *Nde*I/*Xho*I). The gene of Gal-4 was prepared commercially (Biocat GmbH, Germany), and contained a native full-length peptide linker of 28 aa (2979 Da); its subunits Gal-4N and Gal-4C contained a 10 aa part of the peptide linker to preserve the lectin activity.[8–11,14] The genes of Gal-8N, and Gal-8C contained a native full-length peptide linker of 34 aa (3717 Da), isoform PCTA1.[16] The Gal-9 gene carried a truncated HPPYPMPF linker for enhanced stability and solubility,[17] the same as its subunits Gal-9N and Gal-9C.

For biolayer interferometry (BLI), selectively mono-biotinylated galectin constructs were generated. *In vivo* monobiotinylation during heterologous expression was directed to a single lysine in the AVI-tag sequence (GLNDIFEAQ**K**IEWHE) under the catalysis by a co-expressed biotin ligase. An AVI-tag was added to the flexible N-terminal domain of Gal-3, while a 15-residue linker was introduced between the AVI-tag and Gal-1 to preserve its lectin activity. Gal-8-AVI-link, Gal-8N-AVI-link, Gal-8C-AVI-link, Gal-9-AVI-link, Gal-9N-AVI-link and Gal-9C-AVI-link constructs were prepared by cloning the respective genes as specified above into the expression vector pET-Duet1 containing the AVI-tag followed by a 15 aa peptide linker. A pair of synthetic nucleotide sequences (Gal-8-AVI-link: 5′-AAAAACTAGTGCGATGTTGTCC-3′ and 5′-AAAACGGCCGCATTCTTACTAC-3′; Gal-8C-AVI-link: 5′-AAAAACTAGTGCGTTCAGCTCGGAC-3′ and 5′-AAAAGCGGCCGCATTCTTACTACCAG-3′; Gal-9-AVI-link: 5′-

AAAAACTAGTGGGATGGCCTTCAGCGG-3′ and 5′-AAAAGCGGCCGCCTATGTCTGCAC-3′; Gal-9N-AVI-link: 5′-AAAAACTAGTGGGATGGCCTTCAGCGG-3′and 5′-AAAAGCGGCCGCCTAGAAAGGCATC-3′; Gal-9C-AVI-link: 5′-AAAAACTAGTGACCCCCCCTATCCG-3′ and 5′-AAAAGCGGCCGCCTATGTCTGCAC-3′) was annealed to afford a double-stranded DNA fragment carrying the *Spe*I and *Not*I restriction sites. The restricted PCR product was ligated into the *Spe*I/*Not*I-cleaved pETDuet1-Gal-1-AVI-link vector. The isolated plasmids were analyzed by sequencing to confirm the correct subcloning. Genes of Gal-4-AVI-link, Gal-4N-AVI-link, Gal-4C-AVI-link, and Gal-8N-AVI-link containing the AVI-tag and the respective peptide linker as specified above were synthesized commercially (Biocat GmbH, Germany) and cloned into the pET-Duet1 vector (*Asc*I/*Not*I). The gene constructs of AVI-tagged galectins are shown in Figure S29.

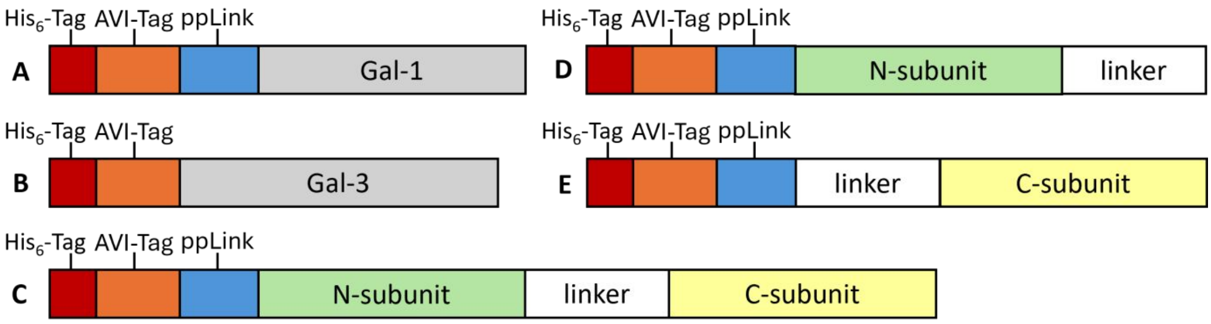

Figure S29. Gene constructs of AVI-galectins. A. Gene construct of Gal-1-AVI-link. **B.** Gene construct of Gal-3-AVI. **C.** AVI-Tagged gene constructs of tandem-repeat galectins (Gal-4-AVI-link, Gal-8-AVI-link, Gal-9-AVI-link). **D.** AVI-Tagged gene constructs of the N-terminal subunits of tandem-repeat galectins (Gal-4N-AVI-link, Gal-8N-AVI-link, Gal-9N-AVI-link). **E.** AVI-Tagged gene constructs of the C-terminal subunit of tandem-repeat galectins (Gal-4C-AVI-link, Gal-8C-AVI-link, Gal-9C-AVI-link).

6.2. Production and Purification of Galectins

The His-tagged galectin constructs were produced as described previously.[8–11,14] Briefly, the His-tagged galectins were produced by transforming *E. coli* Rosetta 2(DE3)pLysS cells, which were cultivated in Luria-Bertani medium (LB; 60 mL, 10 g/L tryptone, 5 g/L NaCl, 5 g/L yeast extract) at 37 °C and 220 rpm overnight. Then the precultures were transferred to Terrific Broth medium (TB; 600 mL; 12 g/L tryptone, 24 g/L yeast extract, 4 mL/L glycerol, 2.3 g/L KH_2PO_4, 12.5 g/L K_2HPO_4) supplemented with ampicillin (100 μg/mL) and chloramphenicol (34 μg/mL) and cultivated at 37 °C and 140 rpm. Protein expression was induced with 0.5 mM IPTG at an OD_{600} of 0.6-0.8, followed by incubation at 25 °C for 24 h. Cells were harvested by centrifugation (8880 × g, 20 min, 4 °C). The AVI-tagged constructs were expressed in *E. coli* BL21(λDE3) cells carrying a plasmid with an IPTG-inducible gene *birA* encoding for biotin ligase as described previously.[18,19] The transformed *E. coli* cells were cultured in MDO medium (20 g/L yeast extract, 20 g/L glycerol, 1 g/L KH_2PO_4, 3 g/L K_2HPO_4, 2 g/L NH_4Cl, 0.5 g/L Na_2SO_4) supplemented with ampicillin (150 μg/mL) and chloramphenicol (10 μg/mL) and cultivated at 37 °C and 140 rpm. When OD_{600} reached 0.6, D-biotin (50 μM; 12 μg/mL) was added along with 1 mM IPTG. The cells were grown for an additional 4 h at 37 °C and harvested by centrifugation (8880 × g, 20 min, 4 °C).

For the purification of galectins, harvested cells were suspended in an equilibration buffer (20 mM phosphate/ 500 mM NaCl/ 20 mM imidazole pH 7.4). Phenylmethylsulfonyl fluoride (PMSF, 1% *v/v*) was added to prevent cleavage by proteases. The suspension was sonicated using UltraSonic Processor UP50H (Ultrasound Technologies, Caldicot, UK) for 6 cycles (1 min pulse, 2 min break on ice). After centrifugation (20,230 × g, 20 min, 4 °C), the cell-free extract was loaded on an equilibrated Ni-NTA column (GE Medical Systems, Prague, Czech Republic). Bound galectins were eluted with an elution buffer containing 500 mM imidazole. In the case of Gal-9, a pre-elution step of washing with 50 mL of 50 mM imidazole was included

to reduce non-specific protein adsorption to the column. Fractions were analyzed for protein content using Bradford assay[6] calibrated for bovine serum albumin (BSA), pooled and dialyzed overnight in PBS buffer pH 7.5 (7 L) containing 2 mM EDTA, followed by 4 h dialysis in PBS buffer (7 L). Gal-1 and Gal-3 proteins were stable at 4 °C for approximately two months; Gal-4, Gal-8, and Gal-9 for ca. four weeks. The purity of prepared galectins was confirmed on SDS-PAGE (12% gel; Figure S30A). The success of biotinylation of AVI-tagged galectins was confirmed by Western Blot (Figure S30B). The lectin activity of AVI-tagged galectins was confirmed by ELISA and the binding affinity (K_D values) to asialofetuin was compared to His-tagged galectins (Table S8).

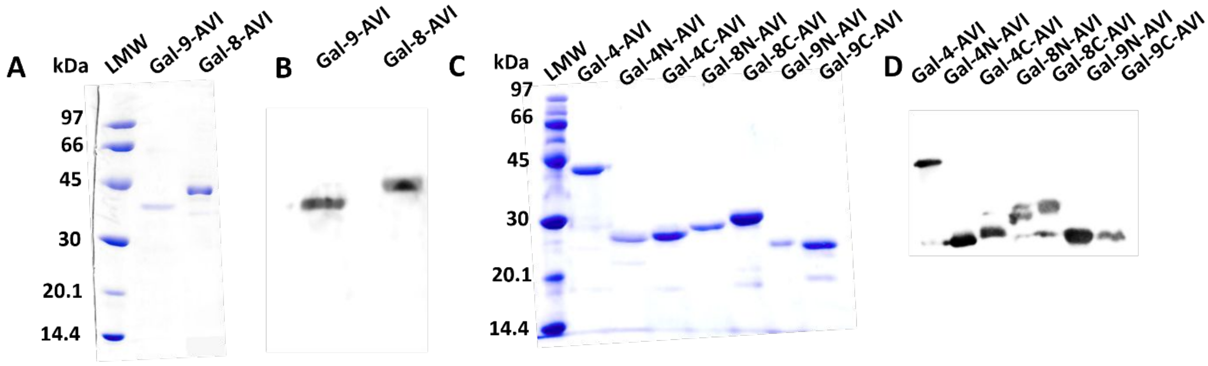

Figure S30. A. SDS-PAGE of Gal-8-AVI, and Gal-9-AVI, 12% gel. **B.** Western blot of Gal-8-AVI, Gal-9-AVI. **C.** SDS-PAGE of Gal-4-AVI, Gal-4N-AVI, Gal-4C-AVI, Gal-8N-AVI, Gal-8C-AVI, Gal-9N-AVI, Gal-9C-AVI, 12% gel. **D.** Western blot of Gal-4-AVI, Gal-4N-AVI, Gal-4C-AVI, Gal-8N-AVI, Gal-8C-AVI, Gal-9N-AVI, Gal-9C-AVI. Samples were loaded with 20 µg protein per lane. The electrophoresis was performed with a 130 V constant voltage. LMW – Amersham Low Molecular Weight Calibration Kit for SDS Electrophoresis (GE Healthcare, Chicago, USA): 97 kDa – phosphorylase b from rabbit muscle; 66 kDa – bovine serum albumin; 45 kDa – chicken egg white ovalbumin; 30 kDa – carbonic anhydrase from bovine erythrocyte; 20.1 kDa – trypsin inhibitor from soybean; 14.4 kDa – α-lactalbumin from bovine milk.

Table S8. Binding affinity of prepared galectins to asialofetuin.

Protein	K_D for ASF [µM][a]	
	His-tag[b]	AVI-tag
Gal-4	3.3 ± 1.1	2.3 ± 0.8
Gal-4N	38 ± 3	17 ± 2
Gal-4C	19 ± 2	6.5 ± 1.2
Gal-8	0.14 ± 0.06	0.32 ± 0.13
Gal-8N	2.3 ± 0.5	4.6 ± 1.3
Gal-8C	9.4 ± 0.4	11 ± 2
Gal-9	0.15 ± 0.04	0.18 ± 0.05
Gal-9N	25 ± 6	11 ± 4
Gal-9C	3.6 ± 0.5	4.9 ± 0.4

[a] K_D values are determined by direct ELISA assay in a procedure analogous to the competitive ELISA assay as detailed in the main text. In the direct ELISA assay, the incubation step comprises only serial dilution of the respective galectin in EPBS (50 µL/well). Galectin concentrations were determined by standard Bradford assay calibrated for γ-globulin (IgG). [b] K_D values for His-tagged proteins were adopted from our previously published work (Gal-4,[11] Gal-8,[19] and Gal-9).[19]

Table S9. Affinity (IC_{50}) of prepared compounds to Gal-1, and Gal-3 determined by competitive ELISA assay.

Compound	Sample	IC_{50}[a]			
		Gal-1	*rp*[b]	Gal-3	*rp*[b]
**1**	Lac	286 ± 23	-	136 ± 18	-
**NGP1**	Lac-HSA	> 20	*< 14*	0.62 ± 0.15	*220*
**7**	LN2	79 ± 9	-	47 ± 6	-
**NGP2**	LN2-HSA	4.6 ± 1.3	*17*	0.15 ± 0.04	*310*
**3**	LN1-Lac	>500	-	15.2 ± 1	-
**NGP3**	LN1-Lac-HSA	0.32 ± 0.11	*> 1500*	0.25 ± 0.09	*61*
**9**	LN1-LN2	>500	-	27 ± 4	-
**NGP4**	LN1-LN2-HSA	> 20	-	0.05 ± 0.02	*540*
**4**	LN2-Lac	436 ± 41	-	7.3 ± 1.6	-
**NGP5**	LN2-Lac-HSA	6.1 ± 1.1	*71*	0.26 ± 0.08	*28*
**10**	LN2-LN2	106 ± 7	-	12 ± 1	-
**NGP6**	LN2-LN2-HSA	6.5 ± 0.8	*16*	0.22 ± 0.14	*54*

**5**	LDN-Lac	435 ± 102	-	2.2 ± 1.3	-
**NGP7**	LDN-Lac-HSA	15 ± 3	*29*	0.08 ± 0.01	*28*
**11**	LDN-LN2	98 ± 22	-	2.7 ± 2.4	-
**NGP8**	LDN-LN2-HSA	> 20	< *5*	0.04 ± 0.01	*68*

[a] IC_{50} (half maximal inhibitory potency) is the concentration of compound required to inhibit galectin binding to immobilized ASF by 50%. Each value was determined in at least quadruplicate; [b] *relative potency* was calculated as the ratio of the affinity of monovalent ligand and the respective neo-glycoprotein

6.3. Production of Galectin Constructs for HSQC and STD Experiments

Both unlabeled and ^{15}N-labeled productions were done as previously described.[20] Briefly, *E. coli* Rosetta (DE3) cells were transformed with the plasmid encoding for the respective full-length galectin construct (Gal-4, Gal-8 or Gal-9). Due to challenges with Gal-9 precipitation, an alternative Gal-9 construct with a modified peptide linker, as previously published,[17] was used for the HSQC titration experiment with **NGP-1** and Gal-9. Then, a single colony was inoculated into 200 mL of LB medium supplemented with 50 μg/mL kanamycin and incubated overnight at 37 °C. The overnight culture was then diluted into 2 L of fresh LB medium containing kanamycin to achieve an initial optical density (OD_{600}) of 0.1 at 600 nm. Cells were grown at 37 °C until their OD_{600} reached 0.6–0.8. Protein expression was induced by adding 1 mM IPTG, and the culture was incubated overnight at 20 °C.

An analogous protocol was used for ^{15}N-labeled protein constructs with minor deviations. Overnight grown transformed cells were transferred to M9 minimal medium containing 1 g/L of $^{15}NH_4Cl$. Upon reaching an OD_{600} of 0.6, protein expression was induced by 1 mM IPTG. The cells were harvested after 24 h incubation at 25 °C.

7. Structural Insights into the Binding Events by Nuclear Magnetic Resonance (NMR)

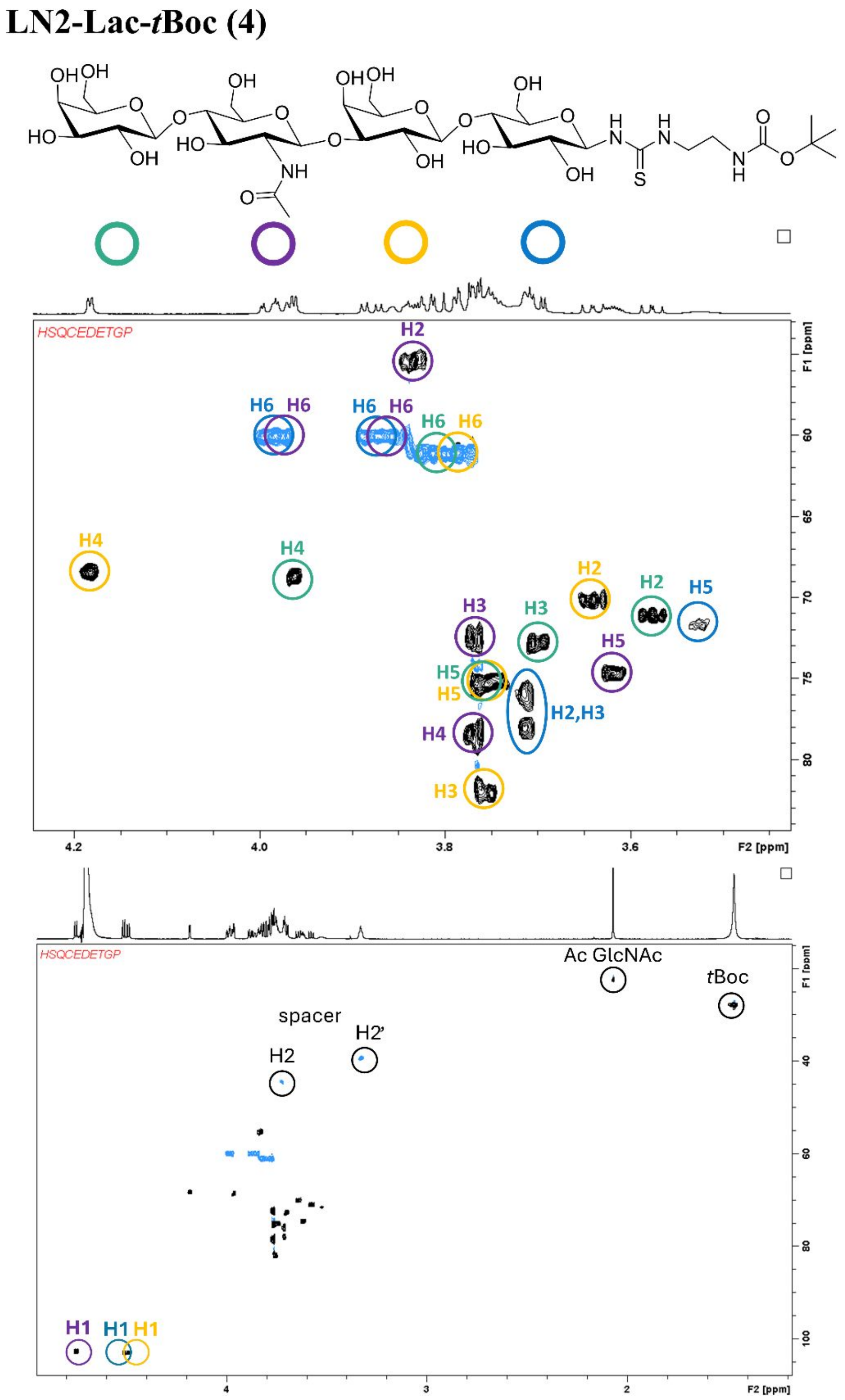

Figure S31 ^{1}H-^{13}C Heteronuclear single quantum coherence (HSQC) spectrum of ligand **LN2-Lac-***t*Boc (**4**) with assigned corresponding protons of respective carbohydrate residues. Blue - Glc; yellow - Gal^{INT}; violet - GlcNAc; green – Gal^{TER}

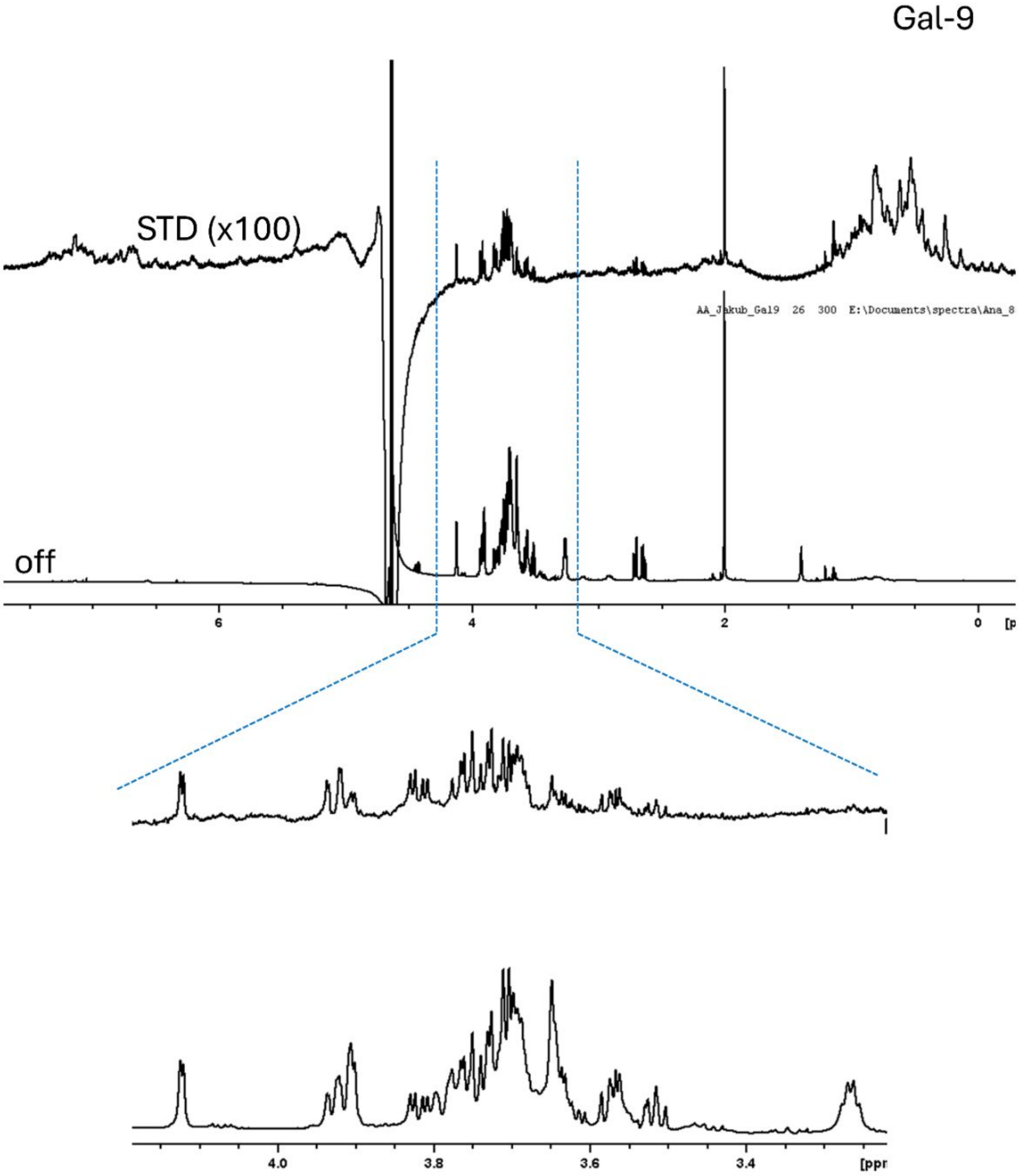

Figure S32. Saturation-transfer difference (STD-NMR) experiment used for epitope mapping with Gal-9, acquired on a sample of Gal-9 (14 µM) + **LN2-Lac**-*t*Boc (700 µM) solution (50:1 ligand-to-galectin ratio). Upper spectrum: differential spectrum (STD-NMR spectrum,) derived from subtraction of the off-resonance and on-resonance spectra. Lower spectrum: off-resonance spectrum (reference).

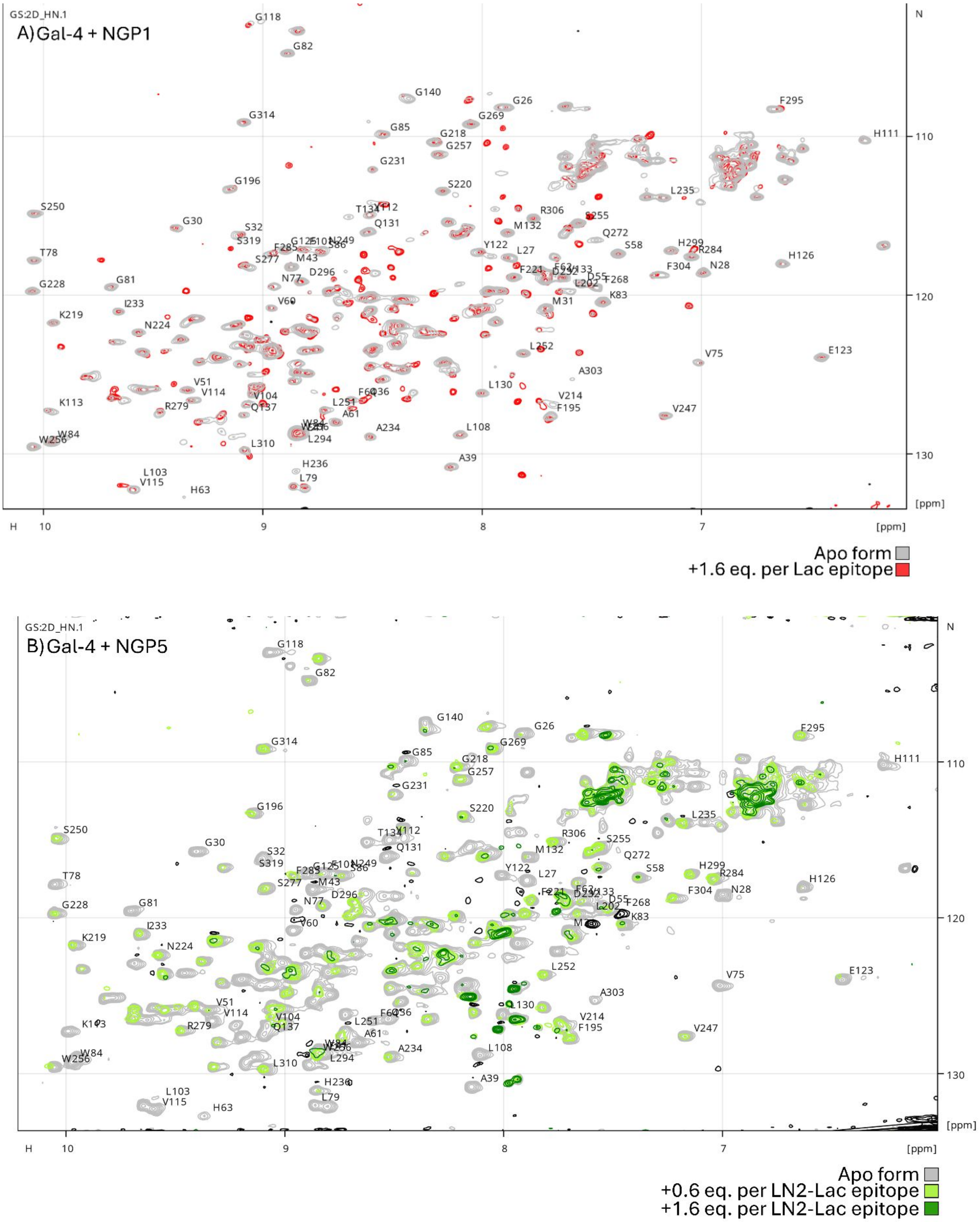

Figure S33. HSQC galectin maps of Gal-4 and their changes after neo-glycoprotein addition. **A**. Gal-4 and **NGP1**; **B**. Gal-4 and **NGP5**.

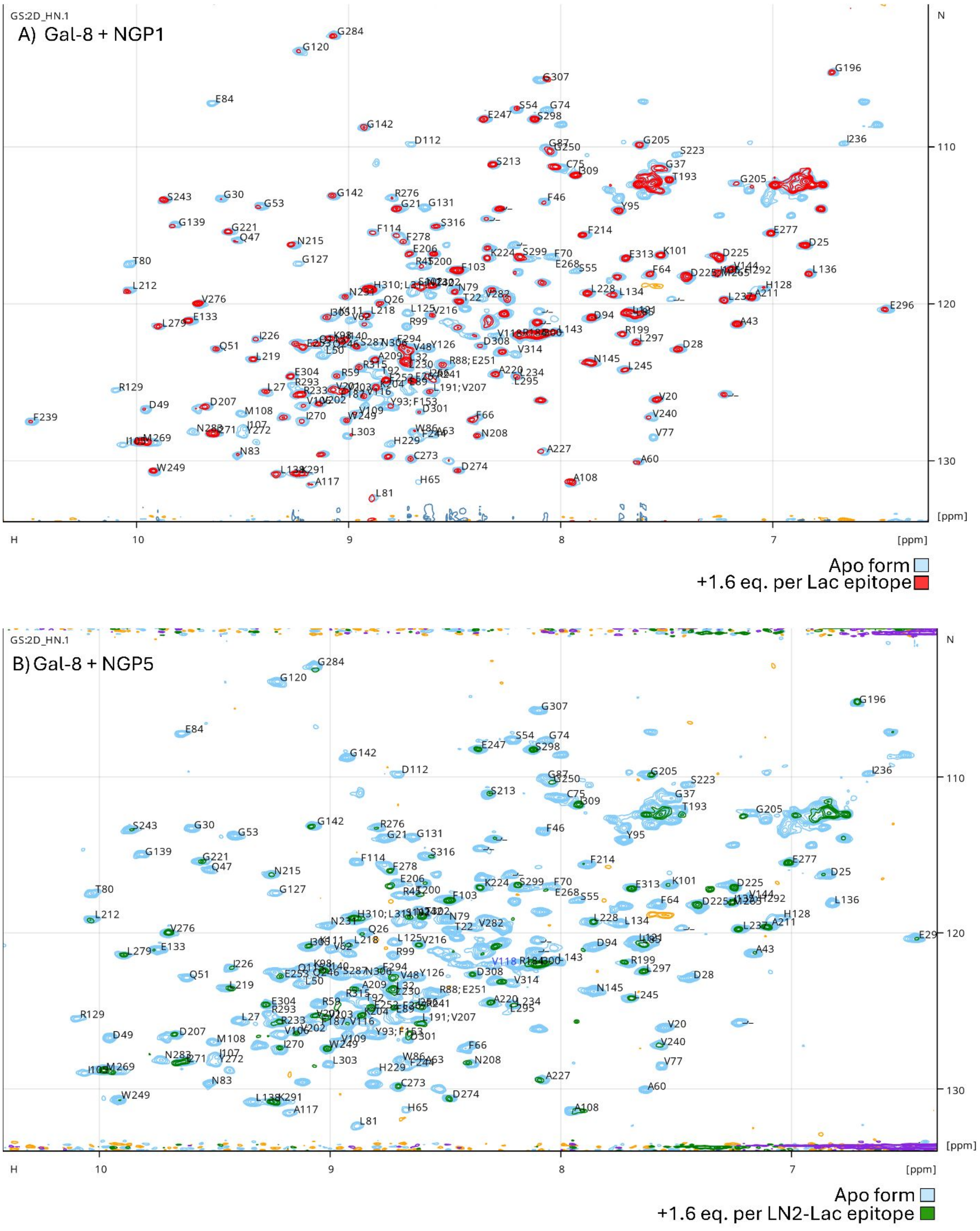

Figure S34. HSQC galectin maps of Gal-8 and their changes after neo-glycoprotein addition. **A**. Gal-8 and **NGP1**; **B**. Gal-8 and **NGP5**.

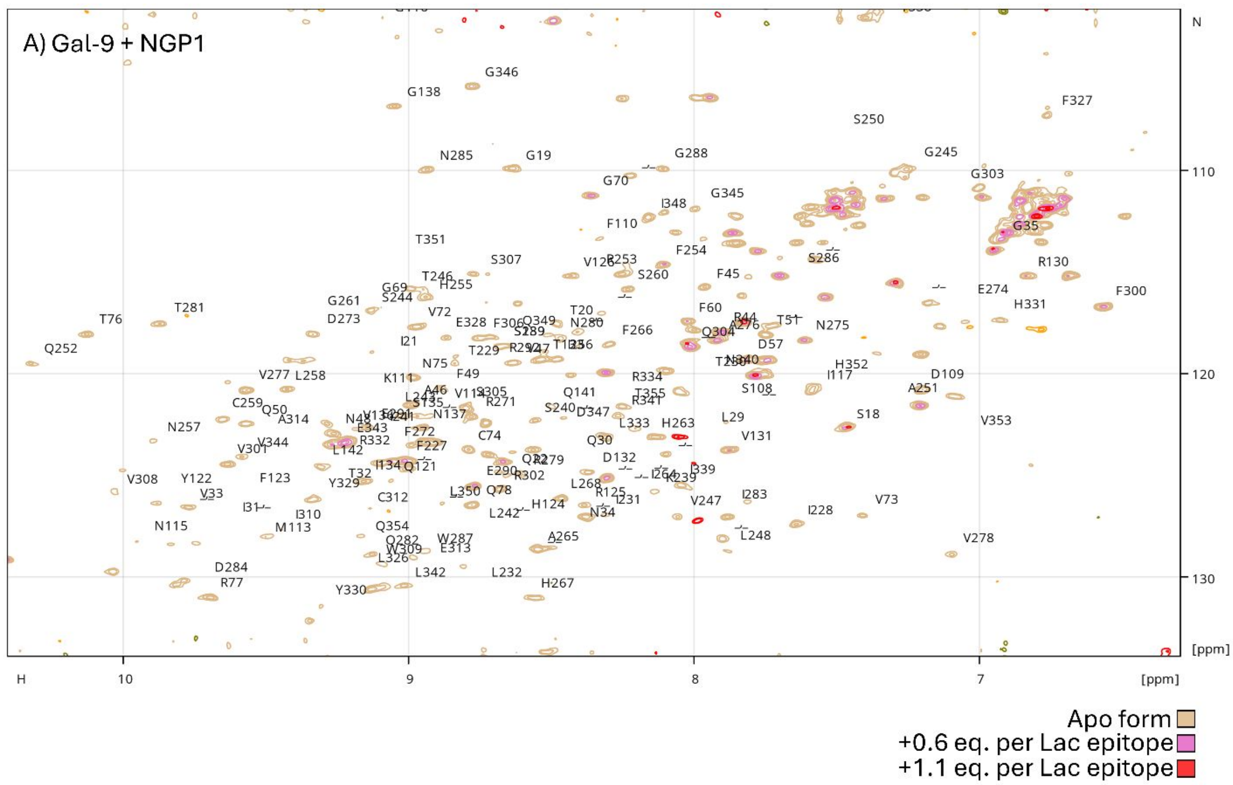

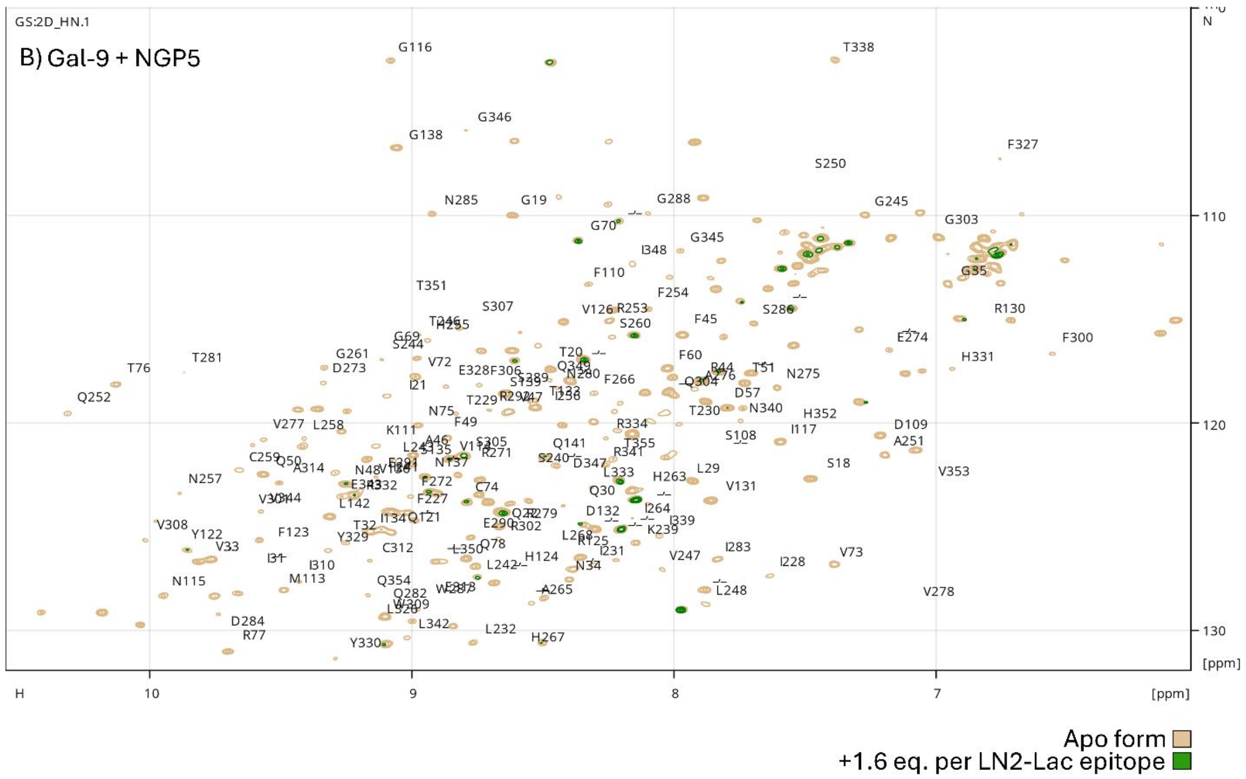

Figure S35. HSQC galectin maps of Gal-9 and their changes after neo-glycoprotein addition. **A**. Gal-9 and **NGP1**; **B**. Gal-8 and **NGP9**.

8. Biolayer Interferometry

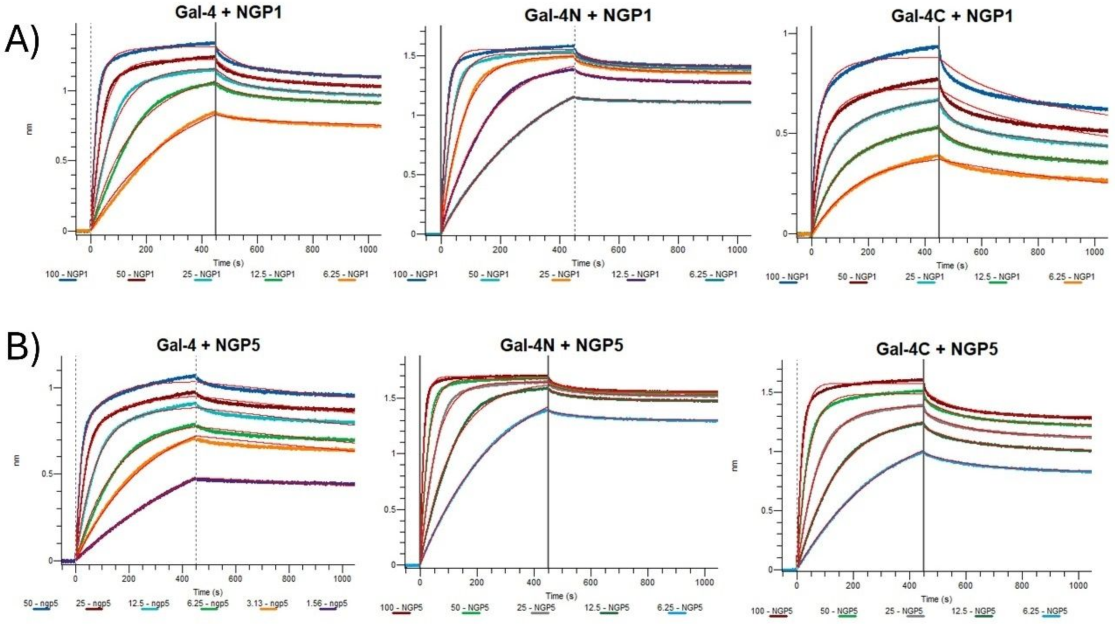

Figure S36. Kinetic analysis of the interaction of neo-glycoproteins with Gal-4 and its subunits. **A**. Interaction with **NGP1**; **B**. Interaction with **NGP5**.

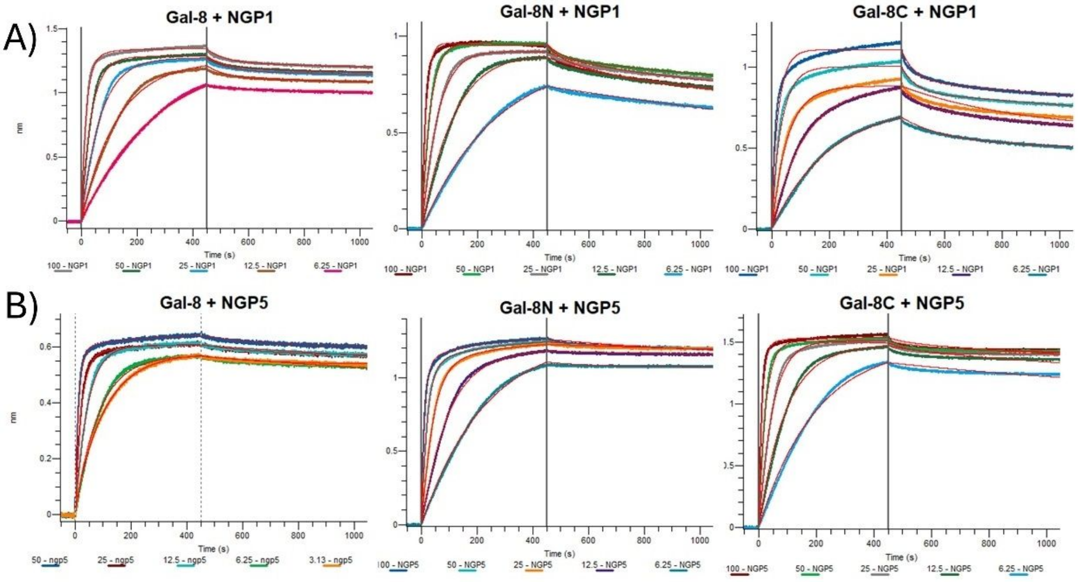

Figure S37. Kinetic analysis of the interaction of neo-glycoproteins with Gal-8 and its subunits. **A**. Interaction with **NGP1**; **B**. Interaction with **NGP5**.

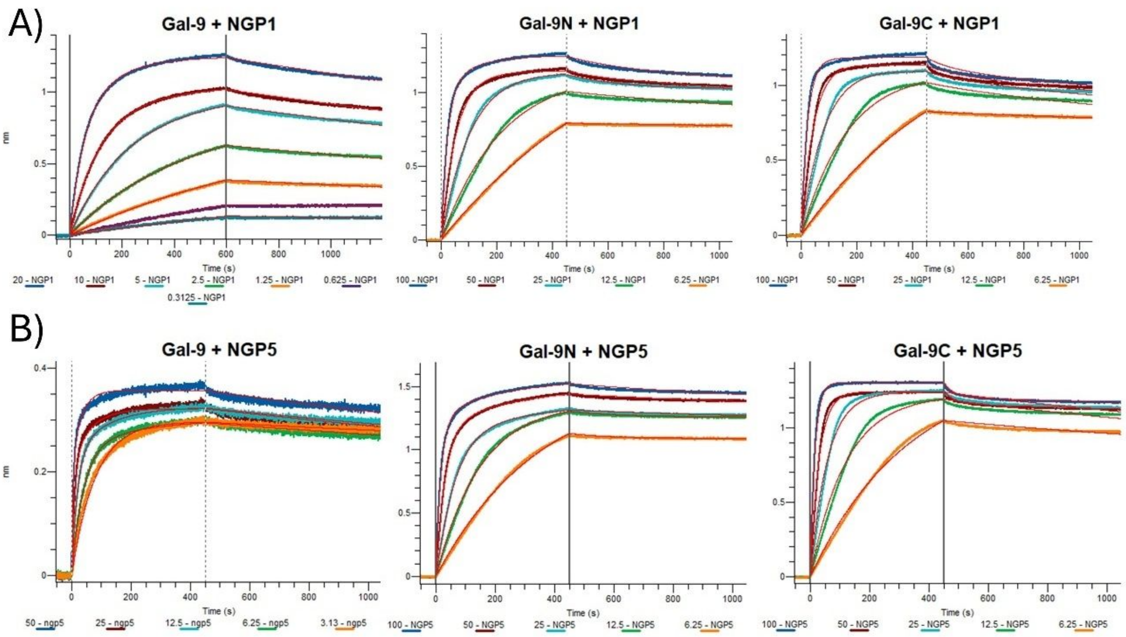

Figure S38. Kinetic analysis of the interaction of neo-glycoproteins with Gal-9 and its subunits. **A**. Interaction with **NGP1**; **B**. Interaction with **NGP5**.

9. Molecular Modeling

Gal-4 binding. The Gal-4 binding scores show a rather large discrepancy between the binding of **LN2-Lac** and **LN2-LN2** in both CRDs with preferential binding to the N-subunit. It was also shown that the **LN2** motif is better accepted than the **LN1** motif in the AB-site of the Gal-4N subunit. This is reflected on fewer interactions with **LN1-Lac** and **LN1-LN2** than with **LN2-Lac** and **LN2-LN2** (Table S10, and Figure S44A for site labeling) and in lower free energies of binding for **LN2-LN2** and **LN2-Lac** for the N-subunit compared with **LN1-LN2** and **LN1-Lac** (Table 3). Gal-4N binds **LN2-LN2** and **LN2-Lac** stronger than Gal-4C (Table 3).

LN1-LN2 is a poor substrate for Gal-4C - after 25 ns of molecular dynamics simulation it got reoriented, and finally lost interactions with many residues from the binding cleft (Figures S40C bottom panel, S44B). This is a result of weak binding of the **LN2** motif in the C-D site of Gal-4C – see Figure S43, and Table S10).

The somewhat higher free energy of binding of **LN2-LN2** together with the lower number of hydrogen bonds in the D-site (Figure S43A) shows that the *N*-acetylated substrate is not well bound in the D-site of Gal-4C (main text, Table 3). The presence of Lys261 in Gal-4C close to the *N*-acetyl group of docked **LN2** determine the preference for **Lac** in the D-site (Figure S40, Table S10B).

Analysis of the interactions of **LN2-Lac** in the Gal-4 binding site revealed other differences between the N- and C-subunits responsible for substrate affinity. The presence of Arg45 in the B-site of the N-subunit (Ser220 in Gal-4C) enabled the formation of an additional hydrogen bond between the *N*-acetyl group and the lectin (Table S10). Hydrophobic Phe47 in the B-site of the N-subunit additionally improves carbohydrate binding through C-H-π interaction (main text, Figure 3A).[21] In the C-subunit we find Ala222 in the corresponding position.

Both subunits showed comparably good **LN1-Lac** binding, which is consistent with the low IC_{50} measured. Notably, the orientation of **LN1-Lac** in the A-site is different from other tetrasaccharides (Figures S40B *vs*. E) due to the presence of the β(1→3) glycosidic linkage. This ligand reorientation enabled the formation of a new hydrogen bond between *N*-acetylglucosamine and Trp256 and a more stable interaction with Gln313 in Gal-4C (Table S10, Figures S40B, E *vs*. G).

In the case of Gal-4N, a slightly better binding score was calculated for **LN2** than for **Lac** (Table 3). The binding scores for both substrates to the C-subunit of Gal-4 are comparable. For the binding scores of Lac in the C- and N-subunits we calculated a rather large standard deviation, which may explain why the calculated average scores are not in such a good agreement with Table 2. Immobilization of the ligand by the linker may also play a role.

The introduction of the *N*-acetyl group in the capping unit of **LDN-Lac** or **LDN-LN2** ligands had no dramatical impact on the binding scores in either Gal-4N or Gal-4C (compared with **LN2-Lac** or **LN2-LN2**, respectively). The analysis of the position of **LN2-LN2** and **LDN-LN2** in Gal-4C revealed that the ligands have different equilibrated positions and interactions (Table S10, Figure S40).

Gal-8 binding. Gal-8 has comparable scores for the disaccharide pair **Lac**, and **LN2**, and for the tetrasaccharide pair **LN2-Lac** and **LN2-LN2** in both CRDs (Figure S39 or main text, Table 3). Slightly better is the binding of **Lac** to Gal-8C and *vice versa* of **LN2-Lac** to Gal-8N as agrees with BLI (Table 2). Of the ligands analyzed, the worst binding score was found between Gal-8N and **Lac**, or **LN2**, and between Gal-8C and **LN1-Lac**, or **LN2**.

Table S11 shows that the terminal **LN1** motif could not be stabilized in the A-site of Gal-8C: **LN1-LN2**, and **LN1-Lac** form only a few hydrogen bonds; stacking or hydrophobic interactions in the A-site with these ligands are also absent (Figure S43). Terminal **LN1** also

formed few interactions in the A-site of the N-subunit, though it still had better scores than the C-subunit. This could be explained by a stronger interaction of terminal **LN1** with the residues in the B-site of Gal-8N as a result of the difference in sequences, namely with Arg59 of the B-C loop (absent in Gal-8C, for details see ref. [19]) – Figure S41, Table S11.

In Gal-8C, it was expected that stacking of the *N*-acetyl group with Arg254 would improve binding as seen in Gal-3.[14] Indeed, we could often see stacking (Figure S41 for **LN2**, and **LN1-LN2**), but **Lac** formed more hydrogen bonds with Gal-8C, which explains its better binding to **Lac** than to **LN2**.

LN2-Lac has a better binding score than other ligands with both CRDs of Gal-8. Together with **LN2-LN2** it is a good ligand as both tetrasaccharides form more hydrogen bonds in the A-site of Gal-8C (Figure S43) than other ligands tested. **LN2-Lac** forms an additional hydrogen bond with Asn306 while both form hydrogen bonds with Glu304, Asn215, Asp217 in A-B sites (Figure 3B in the main text, Table S11). Asn306 is also involved in the stabilization of **LDN-Lac** in the A-site of Gal-8C. This residue is replaced by the larger Gln313 in Gal-4C. From the hydrogen bond frequency analysis (Tables S10-S11) we can see that though Gln313 in Gal-4C also forms hydrogen bonds with **LN2-Lac**, **LN2-LN2** and **LDN-Lac** during molecular dynamics simulation, here the bulkier side chain length of the residue forces the ligand to be positioned further from the binding groove than in Gal-8C, resulting in fewer hydrogen bonds in total formed in the A-B sites of Gal-4C (Tables S12A, B).

LN2-Lac binds with a better score in the N-subunit than in the C-subunit, but the calculated ratio of subunit scores is smaller than that found in the main text, Table 3. Tyr141 (residue absent in Gal-4, Gal-9 and Gal-8C) in the A-site forms C-H/π stacking with the terminal galactose and could be responsible for a stronger binding of **LN2-Lac** in Gal-8N than in Gal-8C (main text, Figure 3).

The ligands **LDN-Lac** and **LDN-LN2** bind more weakly to Gal-8C (fewer hydrogen bonds in the A-B site and in total than with **LN2-Lac**, Figure S43B) than to Gal-8N. The *N*-acetylglucosamine of **LDN-LN2** in the D-site of Gal-8C has different interacting partners (Table S11) as a result of a ligand shift from the original binding site. A significant improvement in the binding score for **LDN-Lac** and **LDN-LN2** with Gal-8N was observed compared with Gal-8C (main text, Table 3). Similar to **LN2-Lac**, these ligands are stabilized in the A-site of Gal-8N by C-H/π-stacking with Tyr141. An additional improvement in binding to the **LDN**-capped ligands in the A-B site of Gal-8N is achieved by a stronger interaction with Arg59. In **LN2-Lac** the galactose hydroxyl interacts with Arg59 whereas in **LDN-Lac** and **LDN-LN2** the interaction with Arg59 is with the carbonyl of the *N*-acetyl group. Hydrophobic interactions generally play an important role in the binding of **LDN-Lac** and **LDN-LN2** to Gal-8 (Figure S43B). The *N*-acetyl group of the terminal *N*-acetylglucosamine forms a hydrophobic interaction with Leu81, Val62 and Ala60 in Gal-8N and with Ile226, Leu219 in Gal-8C (Figures S40-S42).

Gal-9 binding. We observed stronger binding (lower free energies of binding) of the terminal **LN1** motif in the C-subunit of Gal-9 whereas, in contrast, the terminal **LN2** motif was better accepted in the N-subunit (Table 3). This difference between both subunits is more pronounced in the poly-LacNAc ligands.

Gal-9N had a better score for binding **LN2**-based ligands than **Lac**-based ones (main text, Table 3), but Gal-9C slightly prefers **Lac** in the C-D site. The number of hydrogen bonds (Figure S43C), and interaction partners (Table S12, Figure S42) are similar for **LN2** and **Lac** in Gal-9C. The improvement of the binding scores for the **LN2** motif with Gal-9N could be explained by the weak van der Waals interaction of the *N*-acetyl group of **LN2** with Arg87 (Figure S45C). A similar interaction got broken during molecular dynamics simulation with the

corresponding Arg260 of Gal-9C (Figure S45D). In Gal-9C, Arg260 interacts with Glu242 of the C-D loop (Figure S42D bottom panel), hindering the interaction with the *N*-acetyl group. The binding differs between subunits.[19] Therefore, not only the presence of Arg260 plays a role in the observed differences between Gal-9N and Gal-9C, but also its amino acid environment.

The terminal galactosyl in **LN1-LN2** or **LN1-Lac** ligands rarely forms hydrogen bonds in either CRD (Table S12). **LN1-Lac** binds better to Gal-9N than **LN1-LN2**, where the position of the terminal galactose of **LN1-Lac** in the A-site is better stabilized by hydrophobic contacts (Figure S43C).

The hydrogen bond network of **LN2-Lac**, **LN1-LN2** and **LN1-Lac** with the A-B sites of Gal-9C is different. Only **LN2-Lac** forms hydrogen bonds with Arg221, His223 and Gly311, whereas other ligands interact with Asn237 and Glu314 (Table S12, Figure S42).

The improved affinity of Gal-9N to **LN2-LN2** and **LN2-Lac** is a result of the additional hydrogen bonding with Ser54 of the A-B loop of Gal-9N (the loop is shorter in Gal-9C)[19] - Table S12. This interaction is not formed with **LN1-Lac** and **LN1-LN2** due to the more distant positioning of the ligands.

LDN-Lac and **LDN-LN2** bind well to both subunits of Gal-9. The *N*-acetylglucosamine unit is stabilized at the A-site of Gal-9N only by hydrophobic interactions with Gly55, Ile58, and Asn48 (Figure S43C). Charged atoms of Arg77, Asn48 are close to the *N*-acetyl group of *N*-acetylglucosamine in the A-site of Gal-9N and are able to stabilize its position by electrostatic interaction.

Table S10. Frequency of hydrogen bonding formed during a stable period of molecular dynamics simulation run of ligands with Gal-4. The interaction of Gal-4N with **Lac**, **LN1-Lac**, and **LN2-Lac** was analyzed for 60-80 ns; with **LN1-LN2**, **LN2**, **LN2-LN2**, **LDN-Lac**, and **LDN-LN2** for 80-100 ns. The interaction of of Gal-4C with Lac, and **LN1-Lac** was analyzed for 60-80 ns; with **LN1-LN2**, **LN2**, **LN2-Lac**, **LN2-LN2**, **LDN-Lac**, and **LDN-LN2** for 80-100 ns. The first column denotes carbohydrate units at sites A-D (see legend to Figure S42). All interactions with the frequency lower than 0.02 are omitted. Some residues form multiple interactions – with ligand carbohydrate units in more than one site.

A

	Gal-4N	Lac	LN1-Lac	LN1-LN2	LN2	LN2-Lac	LN2-LN2	LDN-Lac	LDN-LN2
D	ARG67	0.96	1.00	0.98	0.98	0.96	0.98	1.00	0.99
	GLU87	0.96	1.00	1.00	1.00	1.00	1.00	1.00	1.00
	ARG89	0.14	0.16	0.26	0.08	0.26	0.30	0.17	0.00
C	ARG45	0.00	0.00	0.00	0.00	0.02	0.00	0.00	0.00
	HIS63	1.00	0.98	0.96	0.98	0.98	0.98	0.98	1.00
	ARG67	0.96	1.00	0.90	0.96	0.90	0.96	0.93	0.89
	ASN77	1.00	1.00	1.00	1.00	0.98	1.00	1.00	1.00
	GLU87	0.98	0.96	1.00	0.96	1.00	1.00	0.97	0.99
B	ARG45	0.00	0.14	0.00	0.00	0.14	0.62	0.00	0.00
	ASN49	0.00	0.92	0.09	0.00	0.96	1.00	1.00	1.00
	TRP84	0.00	0.04	0.00	0.00	0.00	0.00	0.00	0.00
	ASP139	0.00	0.00	0.10	0.00	0.00	0.00	0.00	0.00
A	TYR20	0.00	0.00	0.00	0.00	0.06	0.02	0.31	0.00
	ARG45	0.00	0.00	0.04	0.00	0.00	0.00	0.00	0.00
	GLN137	0.00	0.00	0.00	0.00	0.98	0.80	0.25	0.30
	ASP139	0.00	0.14	0.40	0.00	0.82	0.38	0.07	0.28

B

	Gal-4C	Lac	LN1-Lac	LN1-LN2	LN2	LN2-Lac	LN2-LN2	LDN-Lac	LDN-LN2
D	ARG240	1.00	1.00	0.00	1.00	1.00	0.26	1.00	0.00
	ASN249	0.00	0.00	0.00	0.00	0.00	0.00	0.00	0.35
	GLY257	0.00	0.00	0.00	0.00	0.00	0.00	0.00	0.44
	GLU259	1.00	0.54	0.00	1.00	1.00	0.00	0.69	0.49
	LYS261	0.06	0.00	0.00	0.00	0.10	0.04	0.00	0.00
C	HIS236	1.00	0.98	0.00	0.96	0.98	0.88	0.98	0.00
	ARG240	0.94	0.92	0.00	0.86	0.73	0.56	0.92	0.17
	ASN249	1.00	0.98	0.00	1.00	1.00	1.00	1.00	1.00
	GLU259	0.92	0.50	0.00	0.96	0.98	0.24	0.67	0.49
B	ASN224	0.00	0.00	0.00	0.00	0.98	0.98	0.00	0.00
	HIS236	0.00	0.00	0.00	0.00	0.00	0.00	0.00	0.14
	ASN238	0.00	0.00	0.00	0.00	0.00	0.00	0.00	0.02
	ARG240	0.00	0.00	0.00	0.00	0.00	0.00	0.00	0.08
	TRP256	0.00	0.80	0.00	0.00	0.00	0.00	0.68	0.00
	GLN313	0.00	0.20	0.00	0.00	0.49	0.04	0.53	0.00
A	LYS226	0.00	0.00	0.00	0.00	0.10	0.68	0.00	0.00
	GLU311	0.00	0.02	0.00	0.00	0.37	0.06	0.00	0.00
	GLN313	0.00	0.48	0.00	0.00	0.15	0.30	0.22	0.65
	GLY314	0.00	0.04	0.00	0.00	0.00	0.00	0.00	0.00

Table S11. Frequency of hydrogen bonding formed during a stable period of molecular dynamics simulation run of ligands with Gal-8. The interaction of Gal-8N with **LN1-Lac**, **LN2**, and **LN2-Lac** was analyzed for 50-70 ns; with **Lac**, **LN1-LN2**, **LN2-LN2**, **LDN-Lac**, and **LDN-LN2** for 80-100 ns. The interaction of Gal-8C with **Lac**, **LN1-LN2**, **LN2-LN2, LDN-Lac**, and **LDN-LN2** was analyzed for 80-100 ns; with **LN1-Lac**, **LN2**, and **LN2-Lac** for 50-70 ns. All interactions with the frequency lower than 0.02 are omitted.

A

	Gal-8N	Lac	LN1-Lac	LN1-LN2	LN2	LN2-Lac	LN2-LN2	LDN-Lac	LDN-LN2
D	ARG45	0.00	0.00	0.84	0.00	0.00	0.00	0.00	
	ARG69	1.00	0.08	0.16	0.92	1.00	0.46	0.98	1.00
	GLU89	0.96	0.06	0.90	0.22	0.04	0.02	1.00	0.93
C	ARG45	0.88	0.96	0.82	0.92	1.00	1.00	1.00	1.00
	GLN47	0.00	0.02	0.70	0.90	0.00	0.00	0.00	0.00
	HIS65	1.00	0.96	0.26	0.78	1.00	0.98	1.00	1.00
	ARG69	0.90	0.10	0.10	0.92	0.88	0.14	0.77	0.83
	ASN79	1.00	1.00	1.00	0.02	1.00	1.00	1.00	1.00
	GLU89	0.92	0.02	0.86	0.00	0.04	0.02	0.98	0.85
B	ARG45	0.00	0.00	0.02	0.00	0.16	0.00	0.15	0.22
	GLN47	0.00	1.00	0.94	0.00	0.90	1.00	0.95	0.90
	ASP49	0.00	0.98	0.96	0.00	1.00	0.88	1.00	1.00
	ARG59	0.00	0.46	0.88	0.00	0.04	0.30	0.00	0.00
A	ARG59	0.00	0.00	0.00	0.00	0.24	0.38	0.54	0.61
	GLY142	0.00	0.10	0.16	0.00	0.00	0.42	0.00	0.00

B

	Gal-8C	Lac	LN1-Lac	LN1-LN2	LN2	LN2-Lac	LN2-LN2	LDN-Lac	LDN-LN2
D	ARG233	1.00	0.20	1.00	0.78	1.00	0.46	1.00	0.00
	ASN242	0.00	0.00	0.00	0.00	0.00	0.00	0.00	0.02
	GLY250	0.00	0.00	0.00	0.00	0.00	0.00	0.00	0.03
	GLU252	0.90	0.32	1.00	0.78	1.00	0.18	1.00	0.00
	ARG254	0.26	0.04	0.18	0.14	0.24	0.00	0.14	0.00
C	HIS229	0.92	0.92	1.00	0.76	1.00	0.44	0.99	0.10
	ARG233	0.96	0.38	0.84	0.72	0.98	0.42	0.92	0.00
	ASN242	1.00	0.98	1.00	0.78	1.00	0.46	0.99	0.99
	GLU252	0.88	0.84	0.98	0.78	0.96	0.02	0.99	0.93
B	ASN215	0.00	0.10	0.64	0.00	0.90	0.00	0.00	0.00
	ASP217	0.00	1.00	1.00	0.00	1.00	0.46	1.00	0.57
	HIS229	0.00	0.02	0.02	0.00	0.02	0.00	0.00	0.03
	ASN306	0.00	0.00	0.00	0.00	0.00	0.02	0.00	0.00
A	ASN215	0.00	0.00	0.00	0.00	0.08	0.46	0.00	0.00
	ASP217	0.00	0.00	0.00	0.00	0.40	0.44	0.00	0.00
	LYS224	0.00	0.00	0.00	0.00	0.00	0.02	0.04	0.03
	GLU304	0.00	0.16	0.00	0.00	0.92	0.46	0.02	0.90
	ASN306	0.00	0.08	0.00	0.00	0.22	0.00	0.39	0.00

Table S12. Frequency of hydrogen bonding formed during a stable period of molecular dynamics simulation run of ligands with Gal-9. The interaction of Gal-9N with **Lac**, **LN1-Lac**, **LN2, LN2-Lac**, **LN2-LN2**, **LDN-Lac**, or **LDN-LN2** it was analyzed for 80-100 ns; with **LN1-LN2** for 55-65 ns. The interaction of Gal-9C with **Lac**, **LN1-Lac**, **LN1-LN2**, **LN2**, **LN2-Lac**, **LN2-LN2**, **LDN-Lac**, and **LDN-LN2** it was analyzed for 80-100 ns. All interactions with the frequency lower than 0.02 are omitted.

A

	Gal-9N	Lac	LN1-Lac	LN1-LN2	LN2	LN2-Lac	LN2-LN2	LDN-Lac	LDN-LN2
D	ARG65	1.00	1.00	0.98	0.70	0.98	0.96	1.00	1.00
	GLU85	0.58	1.00	1.00	1.00	0.22	1.00	0.99	1.00
	ARG87	0.20	0.02	0.02	0.14	0.00	0.08	0.12	0.04
C	ARG44	0.04	0.00	0.00	0.00	0.00	0.02	0.00	0.00
	HIS61	0.94	0.96	0.98	0.78	1.00	0.98	1.00	0.98
	ARG65	0.94	0.88	0.88	0.94	0.64	0.84	0.97	0.96
	ASN75	1.00	1.00	1.00	1.00	1.00	1.00	1.00	1.00
	GLU85	0.48	0.98	1.00	0.98	0.10	0.98	0.96	0.99
B	ARG44	0.00	0.58	0.60	0.00	0.68	0.10	0.38	0.12
	ASN48	0.00	1.00	0.94	0.00	1.00	0.96	0.86	0.96
	HIS61	0.00	0.02	0.00	0.00	0.00	0.00	0.00	0.00
	ASN137	0.00	0.88	0.82	0.00	0.96	1.00	0.98	0.95
A	SER54	0.00	0.00	0.00	0.00	0.30	0.66	0.00	0.00
	GLY55	0.00	0.00	0.00	0.00	0.16	0.00	0.00	0.00
	ARG77	0.00	0.00	0.02	0.00	0.00	0.02	0.00	0.00
	ASN137	0.00	0.02	0.00	0.00	0.00	0.00	0.00	0.00
	GLY138	0.00	0.06	0.00	0.00	0.00	0.00	0.00	0.00

B

	Gal-9C	Lac	LN1-Lac	LN1-LN2	LN2	LN2-Lac	LN2-LN2	LDN-Lac	LDN-LN2
D	ARG239	1.00	1.00	1.00	1.00	1.00	1.00	1.00	1.00
	GLU258	1.00	1.00	1.00	1.00	1.00	0.96	0.77	0.70
	ARG260	0.16	0.30	0.04	0.16	0.14	0.02	0.09	0.07
C	ARG221	0.00	0.00	0.00	0.00	0.02	0.00	0.00	0.00
	HIS235	1.00	0.92	0.98	0.98	1.00	1.00	1.00	0.99
	ARG239	0.90	0.94	0.98	0.90	0.92	0.94	0.96	0.96
	ASN248	1.00	1.00	1.00	0.98	0.98	1.00	1.00	1.00
	GLU258	0.90	0.96	0.94	0.96	0.98	0.72	0.58	0.66
B	ARG221	0.00	0.00	0.00	0.00	0.08	0.00	0.04	0.47
	HIS223	0.00	0.06	0.00	0.00	0.10	0.30	0.03	0.13
	ASN225	0.00	0.92	1.00	0.00	0.94	1.00	1.00	0.93
	ASN237	0.00	0.64	0.10	0.00	0.00	0.00	0.00	0.00
A	HIS223	0.00	0.00	0.00	0.00	0.02	0.00	0.00	0.00
	GLY311	0.00	0.00	0.00	0.00	0.30	0.00	0.02	0.00
	GLU314	0.00	0.00	0.30	0.00	0.00	0.16	0.22	0.33

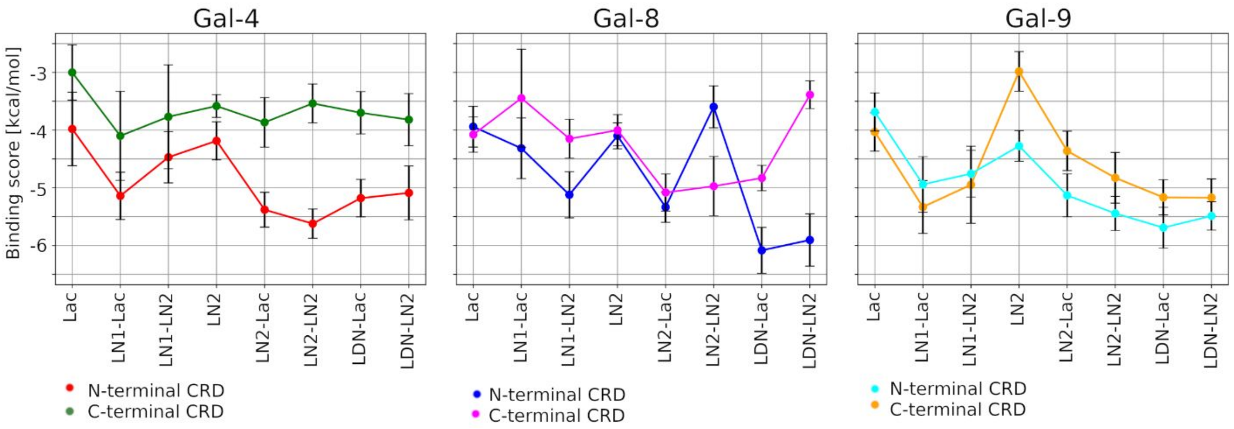

Figure S39. Ligand binding scores, calculated for an equilibrated period of molecular dynamics simulation (shown in Tables S10-S12). Average values are shown by colored dots with standard deviation.

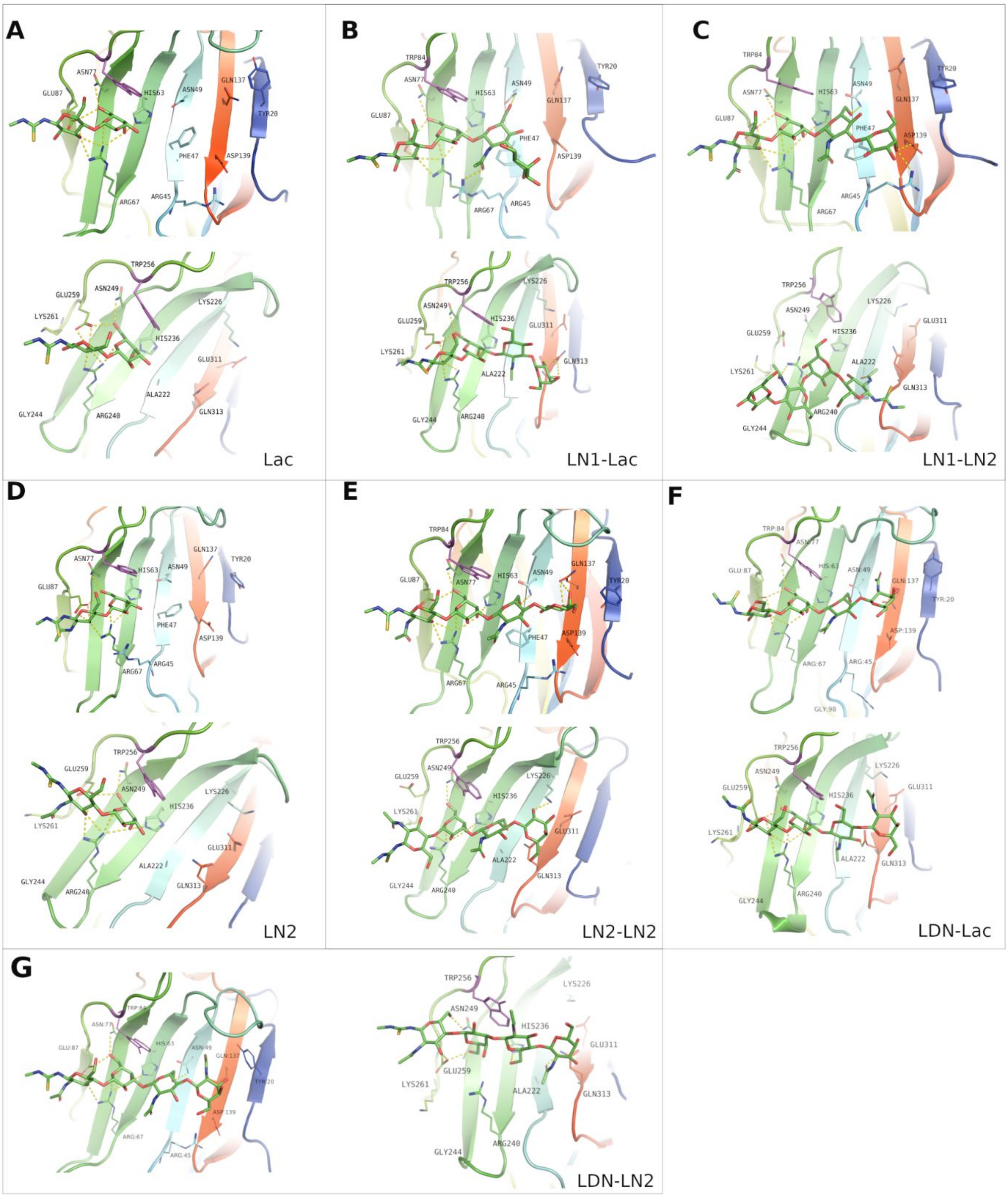

Figure S40. Ligands in the Gal-4 binding site after molecular dynamics simulation (representative snapshot from the stable period). Amino acid residues capable of forming hydrogen bond interactions with carbohydrates are shown and labeled, non-polar hydrogens are hidden. Trp residues forming stacking interactions are shown and colored in magenta. For Gal-4C with LN1-LN2 a snapshot after 100 ns of molecular dynamics simulation is used, for others from the stable period (Table S10). **A.** Lac, **B.** LN1-Lac, **C.** LN1-LN2 where *N*-acetylglucosamine is displaced from the D-site to another orientation above Ala222 and Glu313, **D.** LN2, **E.** LN2-LN2, **F.** LDN-Lac, **G.** LDN-LN2.

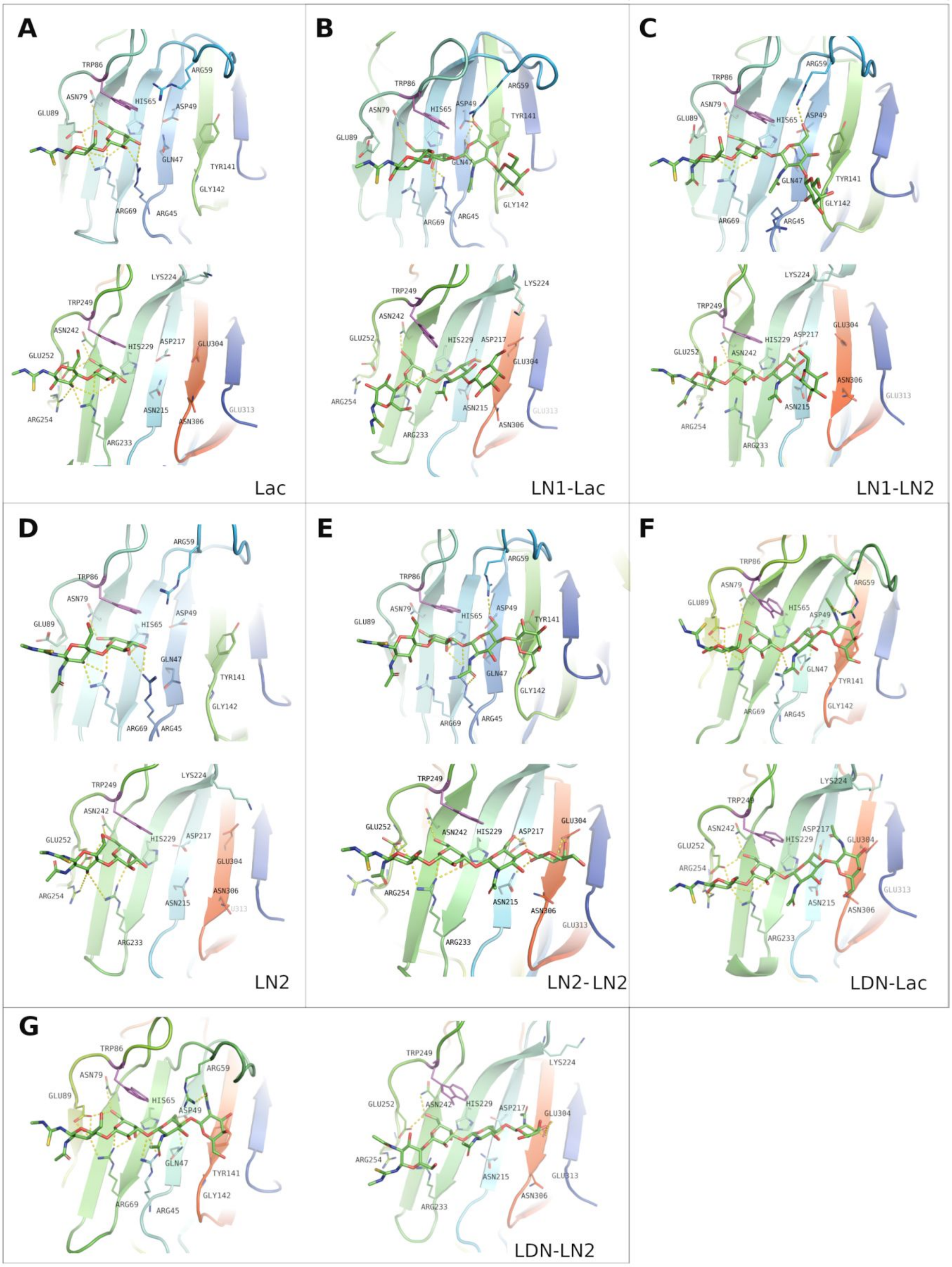

Figure S41. Ligands in the Gal-8 binding site after molecular dynamics simulation (representative snapshot from the stable period - Table S11). Amino acid residues capable of forming hydrogen bond interactions with carbohydrates are shown and labeled, non-polar hydrogens are hidden. Trp residues forming stacking interactions are shown and colored in magenta.

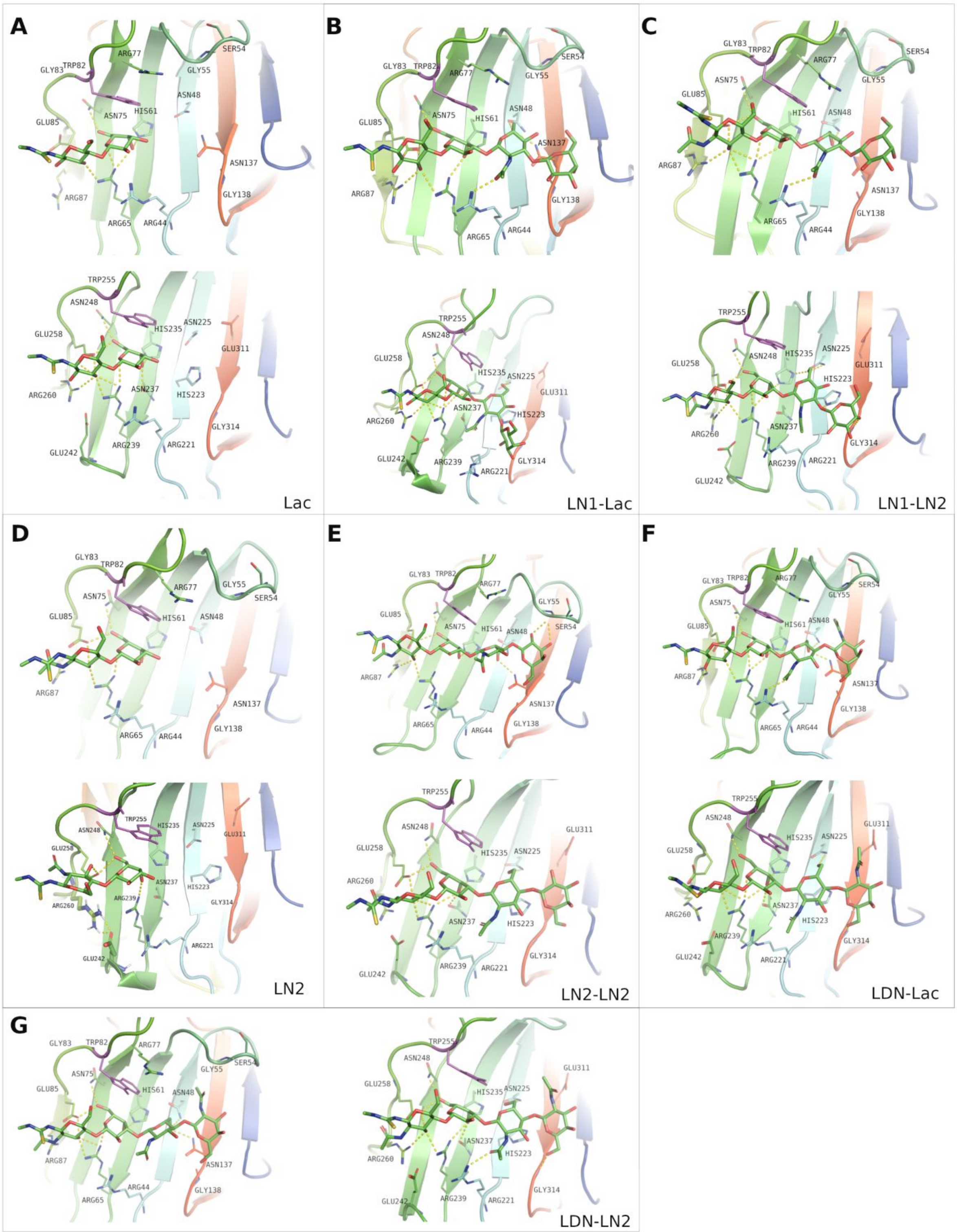

Figure S42. Ligands in the Gal-9 binding site after molecular dynamics simulation (representative snapshot from the stable period - Table S12). Amino acid residues capable of forming hydrogen bond interactions with carbohydrates are shown and labeled, non-polar hydrogens are hidden. Trp residues forming stacking interactions are shown and colored in magenta.

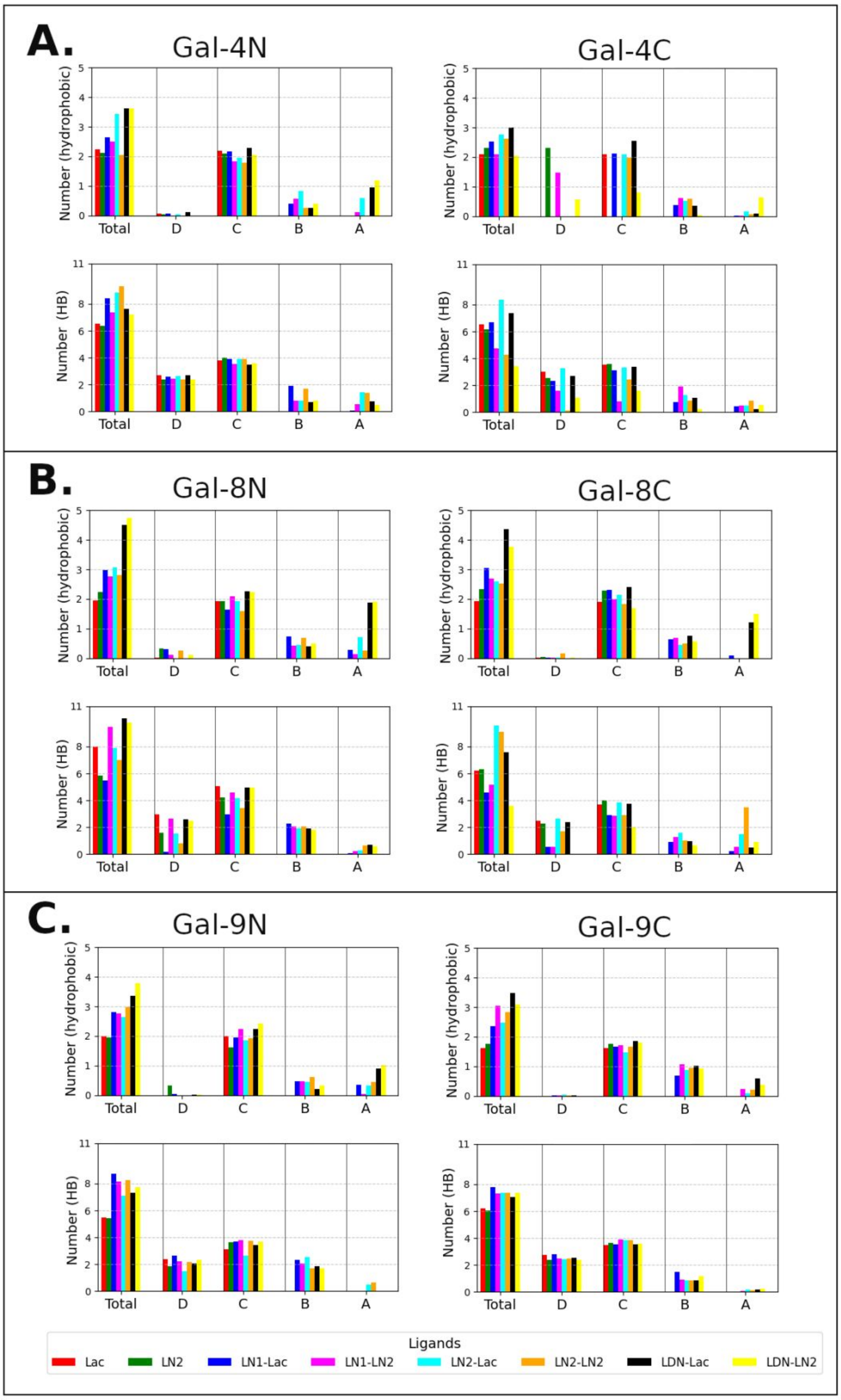

Figure S43. Interactions of ligands with galectins. **A.** Hydrogen bonds and hydrophobic interactions formed by Gal-4 during the stable period of molecular dynamics simulation. Analyzed time span from molecular dynamics simulation of Gal-4N was 60-80 ns for **Lac**, **LN1-Lac**, **LN2-Lac**; 80-100 ns for **LN1-LN2**, **LN2**, **LN2-LN2**. Analyzed time span from molecular dynamics simulation was 60-80 ns of Gal-4C for **Lac**, **LN1-Lac**; 80-100 ns for **LN1-LN2**, **LN2**, **LN2-Lac**, **LN2-LN2**. **B.** Hydrogen bonds and hydrophobic interactions formed by Gal-8

during the stable period of molecular dynamics simulation. Analyzed time span from molecular dynamics simulation for Gal-8N was 50-70 ns for **LN1-Lac**, **LN2**, **LN2-Lac**; 80-100 ns for **Lac**, **LN1-LN2**, **LN2-LN2**. Analyzed time span from molecular dynamics simulation for Gal-8C was 80-100 ns for **Lac**, **LN1-LN2**, **LN2-LN2**; 50-70 ns for **LN1-Lac**, **LN2**, **LN2-Lac**. **C.** Hydrogen bonds and hydrophobic interactions formed by Gal-9 during the stable period of molecular dynamics simulation. Analyzed time span from molecular dynamics simulation for Gal-9N was 80-100 ns for **Lac**, **LN1-Lac**, **LN2**, **LN2-Lac**, **LN2-LN2**; 55-65 ns for **LN1-LN2**. Analyzed time span from molecular dynamics simulation for Gal-9C was 80-100ns for **Lac**, **LN1-Lac**, **LN1-LN2**, **LN2**, **LN2-Lac**, **LN2-LN2**. The labeling of carbohydrate ligand units is shown in Figure S36.

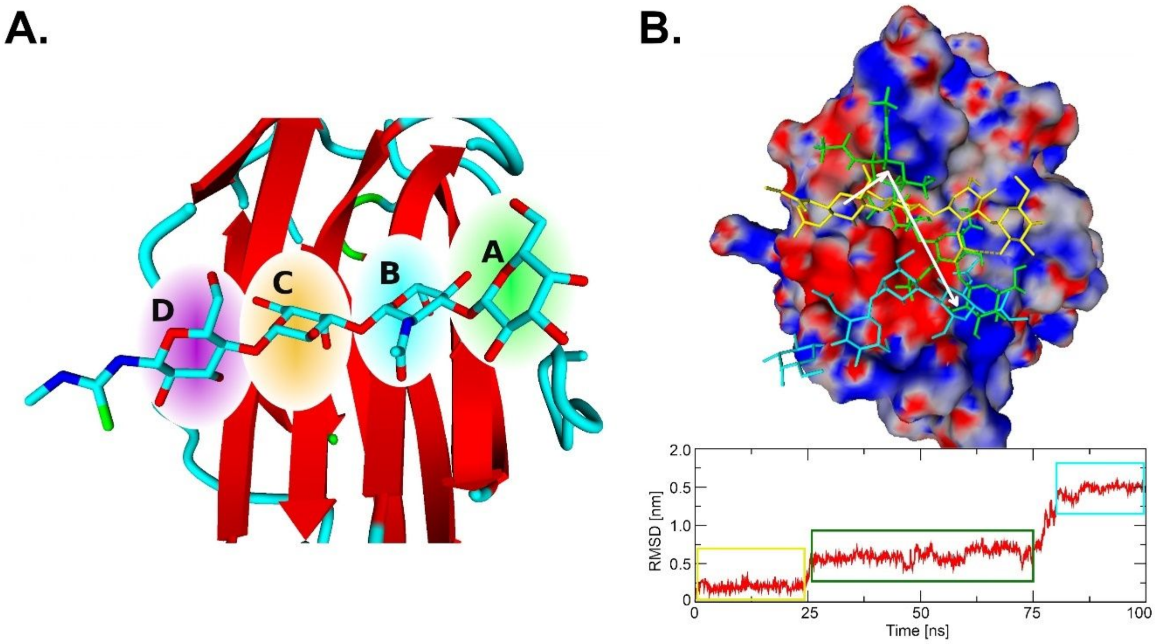

Figure S44. A. Labeling of galectin binding sites. **B.** Gal-4C with **LN1-LN2**. RMSD of the ligand (bottom panel) with corresponding positions of the ligands (upper panel) in the binding groove during molecular dynamics simulation is shown. Ligands are colored according to the rectangular selected regions of RMSD. The displacement of GlcNAc unit from the D-site during molecular dynamics simulation is shown by the white arrow in the upper panel: yellow – 0 ns snapshot, green – 30 ns snapshot, cyan – 100 ns snapshot.

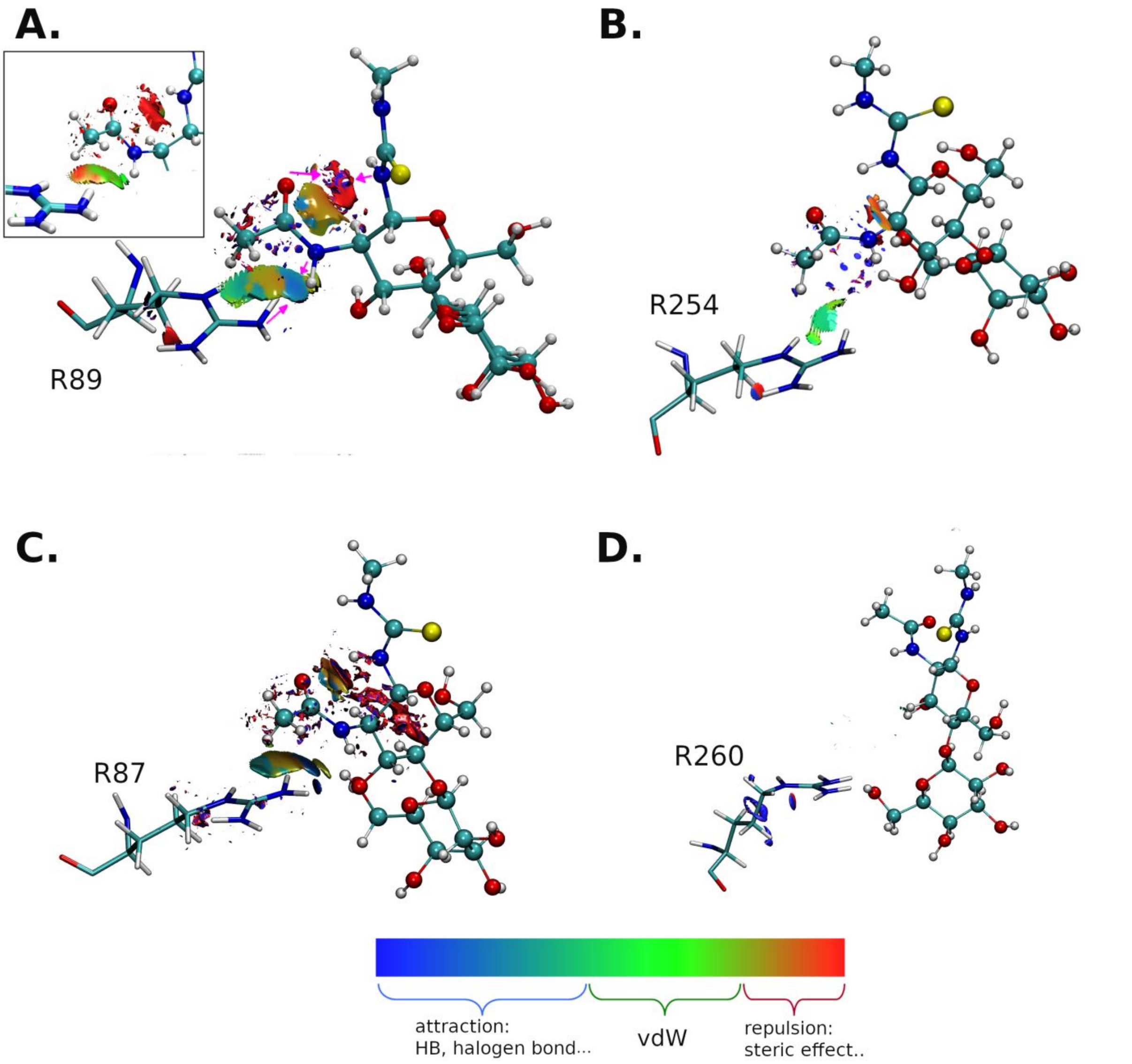

Figure S45. Analysis of a weak interactions between an arginine residue and **LN2** for Gal-4N (**A**); for Gal-8C (**B**), for Gal-9N (**C**), and Gal-9C (**D**) by averaged NCI method for multiple frames[22] with Multiwfn.[23] For NCI method we used snapshots from equlibrated molecular dynamics simulations (500 frames) with grid centering for intarcatino analysis of the guanidine group of arginine. Inset in panel **A** shows the Thermal Fluctuation Index for formed weak interactions (red – more flexible and unstable interaction; blue – more stable interactions). Arrows in panel **A** show atoms participating in more interactions.

10. References

1. Fischöder, T.; Wahl, C.; Zerhusen, C.; Elling, L. Repetitive Batch Mode Facilitates Enzymatic Synthesis of the Nucleotide Sugars UDP-Gal, UDP-GlcNAc, and UDP-GalNAc on a Multi-Gram Scale. *Biotechnol. J.* **2019**, *14*. https://doi.org/10.1002/BIOT.201800386.
2. Sauerzapfe, B.; Namdjou, D. J.; Schumacher, T.; Linden, N.; Křenek, K.; Křen, V.; Elling, L. Characterization of Recombinant Fusion Constructs of Human β1,4-Galactosyltransferase 1 and the Lipase Pre-Propeptide from *Staphylococcus Hyicus. J. Mol. Catal. B Enzym.* **2008**, *50*, 128–140. https://doi.org/10.1016/J.MOLCATB.2007.09.009.
3. Sauerzapfe, B.; Křenek, K.; Schmiedel, J.; Wakarchuk, W. W.; Pelantová, H.; Křen, V.; Elling, L. Chemo-Enzymatic Synthesis of Poly-*N*-Acetyllactosamine (Poly-LacNAc) Structures and Their Characterization for CGL2-Galectin-Mediated Binding of ECM Glycoproteins to Biomaterial Surfaces. *Glycoconjug. J.* **2009**, *26*, 141–159. https://doi.org/10.1007/s10719-008-9172-2.
4. Kupper, C. E.; Rosencrantz, R. R.; Henßen, B.; Pelantová, H.; Thönes, S.; Drozdová, A.; Kren, V.; Elling, L. Chemo-Enzymatic Modification of Poly-*N*-Acetyllactosamine (LacNAc) Oligomers and *N,N'*-Diacetyllactosamine (LacDiNAc) Based on Galactose Oxidase Treatment. *Beilstein J. Org. Chem.* **2012**, *8*, 712–725. https://doi.org/10.3762/bjoc.8.80.
5. Henze, M.; You, D. J.; Kamerke, C.; Hoffmann, N.; Angkawidjaja, C.; Ernst, S.; Pietruszka, J.; Kanaya, S.; Elling, L. Rational Design of a Glycosynthase by the Crystal Structure of β-Galactosidase from Bacillus Circulans (BgaC) and Its Use for the Synthesis of *N*-Acetyllactosamine Type 1 Glycan Structures. *J. Biotechnol.* **2014**, *191*, 78–85. https://doi.org/10.1016/J.JBIOTEC.2014.07.003.
6. Bradford, M. M. A Rapid and Sensitive Method for the Quantitation of Microgram Quantities of Protein Utilizing the Principle of Protein-Dye Binding. *Anal. Biochem.* **1976**, *72*, 248–254. https://doi.org/10.1016/0003-2697(76)90527-3.
7. Hovorková, M.; Kulik, N.; Konvalinková, D.; Petrásková, L.; Křen, V.; Bojarová, P. Mutagenesis of Catalytic Nucleophile of β-Galactosidase Retains Residual Hydrolytic Activity and Affords a Transgalactosidase. *ChemCatChem* **2021**, *13*, 4532–4542. https://doi.org/10.1002/CCTC.202101107.
8. Vašíček, T.; Spiwok, V.; Červený, J.; Petrásková, L.; Bumba, L.; Vrbata, D.; Pelantová, H.; Křen, V.; Bojarová, P. Regioselective 3-O-Substitution of Unprotected Thiodigalactosides: Direct Route to Galectin Inhibitors. *Chem. Eur. J.* **2020**, *26*, 9620–9631. https://doi.org/10.1002/chem.202002084.
9. Konvalinková, D.; Dolníček, F.; Hovorková, M.; Červený, J.; Kundrát, O.; Pelantová, H.; Petrásková, L.; Cvačka, J.; Faizulina, M.; Varghese, B.; Kovaříček, P.; Křen, V.; Lhoták, P.; Bojarová, P. Glycocalix[4]Arenes and Their Affinity to a Library of Galectins: The Linker Matters. *Org. Biomol. Chem.* **2023**, *21*, 1294–1302. https://doi.org/10.1039/d2ob02235d.
10. Müllerová, M.; Hovorková, M.; Závodná, T.; Červenková Šťastná, L.; Krupková, A.; Hamala, V.; Nováková, K.; Topinka, J.; Bojarová, P.; Strašák, T. Lactose-Functionalized Carbosilane Glycodendrimers Are Highly Potent Multivalent Ligands

for Galectin-9 Binding: Increased Glycan Affinity to Galectins Correlates with Aggregation Behavior. *Biomacromolecules* **2023**, *24*, 4705–4717. https://doi.org/10.1021/ACS.BIOMAC.3C00426.

11. Slámová, K.; Červený, J.; Mészáros, Z.; Friede, T.; Vrbata, D.; Křen, V.; Bojarová, P. Oligosaccharide Ligands of Galectin-4 and Its Subunits: Multivalency Scores Highly. *Molecules* **2023**, *28*, 4039. https://doi.org/10.3390/molecules28104039.
12. Tavares, M. R.; Bláhová, M.; Sedláková, L.; Elling, L.; Pelantová, H.; Konefał, R.; Etrych, T.; Křen, V.; Bojarová, P.; Chytil, P. High-Affinity *N*-(2-Hydroxypropyl)Methacrylamide Copolymers with Tailored *N*-Acetyllactosamine Presentation Discriminate between Galectins. *Biomacromolecules* **2020**, *21*, 641–652. https://doi.org/10.1021/acs.biomac.9b01370.
13. Laaf, D.; Bojarová, P.; Pelantová, H.; Křen, V.; Elling, L. Tailored Multivalent Neo-Glycoproteins: Synthesis, Evaluation, and Application of a Library of Galectin-3-Binding Glycan Ligands. *Bioconjug. Chem.* **2017**, *28*, 2832–2840. https://doi.org/10.1021/acs.bioconjchem.7b00520.
14. Bumba, L.; Laaf, D.; Spiwok, V.; Elling, L.; Křen, V.; Bojarová, P. Poly-*N*-Acetyllactosamine Neo-Glycoproteins as Nanomolar Ligands of Human Galectin-3: Binding Kinetics and Modeling. *Int. J. Mol. Sci.* **2018**, *19*, 372. https://doi.org/10.3390/ijms19020372.
15. Hirabayashi, J.; Kasai, K. I. Effect of Amino Acid Substitution by Site-Directed Mutagenesis on the Carbohydrate Recognition and Stability of Human 14-KDa β-Galactoside-Binding Lectin. *J. Biol. Chem.* **1991**, *266*, 23648–23653. https://doi.org/10.1016/s0021-9258(18)54333-7.
16. Bidon-Wagner, N.; Le Pennec, J. P. Human Galectin-8 Isoforms and Cancer. *Glycoconj. J.* **2002**, *19*, 557–563. https://doi.org/10.1023/B:GLYC.0000014086.38343.98.
17. Itoh, A.; Fukata, Y.; Miyanaka, H.; Nonaka, Y.; Ogawa, T.; Nakamura, T.; Nishi, N. Optimization of the Inter-Domain Structure of Galectin-9 for Recombinant Production. *Glycobiology* **2013**, *23*, 920–925. https://doi.org/10.1093/glycob/cwt023.
18. Hovorková, M.; Červený, J.; Bumba, L.; Pelantová, H.; Cvačka, J.; Křen, V.; Renaudet, O.; Goyard, D.; Bojarová, P. Advanced High-Affinity Glycoconjugate Ligands of Galectins. *Bioorg. Chem.* **2023**, *131*, 106279. https://doi.org/10.1016/j.bioorg.2022.106279.
19. Vrbata, D.; Červený, J.; Kulik, N.; Hovorková, M.; Balogová, S.; Vlachová, M.; Pelantová, H.; Křen, V.; Bojarová, P. Glycomimetic Inhibitors of Tandem-Repeat Galectins: Simple and Efficient. *Bioorg. Chem.* **2024**, *145*, 107231. https://doi.org/10.1016/j.bioorg.2024.107231.
20. Quintana, J. I.; Massaro, M.; Cagnoni, A. J.; Nuñez-Franco, R.; Delgado, S.; Jiménez-Osés, G.; Mariño, K. V.; Rabinovich, G. A.; Jiménez-Barbero, J.; Ardá, A. Different Roles of the Heterodimer Architecture of Galectin-4 in Selective Recognition of Oligosaccharides and Lipopolysaccharides Having ABH Antigens. *J. Biol. Chem.* **2024**, *300*, 107577. https://doi.org/10.1016/J.JBC.2024.107577.
21. Hudson, K. L.; Bartlett, G. J.; Diehl, R. C.; Agirre, J.; Gallagher, T.; Kiessling, L. L.; Woolfson, D. N. Carbohydrate-Aromatic Interactions in Proteins. *J. Am. Chem. Soc.* **2015**, *137*, 15152–15160. https://doi.org/10.1021/jacs.5b08424.

22. Cao, S.; Wang, J.; Ding, Y.; Sun, M.; Ma, F. Visualization of Weak Interactions between Quantum Dot and Graphene in Hybrid Materials. *Sci. Rep.* **2017**, *7*, 1–9. https://doi.org/10.1038/s41598-017-00542-9.

23. Lu, T.; Chen, F. Multiwfn: A Multifunctional Wavefunction Analyzer. *J. Comput. Chem.* **2012**, *33*, 580–592. https://doi.org/10.1002/JCC.22885.
